# Sitagliptin Reduces Endothelial Dysfunction and Apoptosis Induced by High-Fat Diet and Palmitate in Thoracic Aortas and Endothelial Cells via ROS-ER Stress-CHOP Pathway

**DOI:** 10.3389/fphar.2021.670389

**Published:** 2021-08-31

**Authors:** Qiongqiong Cao, Dongmei Xu, Yong Chen, Yueming Long, Fang Dai, Li Gui, Yunxia Lu

**Affiliations:** ^1^ Department of Biochemistry and Molecular Biology, Hefei, China; ^2^ Hefei Lifeon Pharmaceutical Co. Ltd., Hefei, China; ^3^ Department of Endocrinology, The First Affiliated Hospital of Anhui Medical University, Hefei, China; ^4^ The Comprehensive Laboratory, School of Basic Medical Science, Anhui Medical University, Hefei, China

**Keywords:** sitagliptin, high-fat diet, palmitate, endoplasmic reticulum stress, thoracic aorta, human umbilical vein endothelial cells, ROS

## Abstract

Macrovascular disease is tightly associated with obesity-induced metabolic syndrome. Sitagliptin (SIT), an orally stable selective inhibitor of Dipeptidyl peptidase-4 (DPP-4), has protective effects on endothelium. However, the mechanisms enabling SIT to exhibit resistance to diet-induced obesity (DIO) related with reactive oxygen species (ROS) and endoplasmic reticulum (ER) stress in the aorta and endothelial cells have not been reported yet. Therefore, the present study was conducted to determine if SIT exerts protective role in the thoracic aortas isolated from the high-fat diet (HFD)-treated rats and palmitate (PA)-treated endothelial cells by alleviating ROS and ER stress. Male Sprague Dawley rats were randomly divided into standard chow diet (SCD), HFD and HFD plus sitagliptin administration (HFD + SIT) groups. The rats of latter two groups were given HFD fodder for 12 weeks, then the HFD + SIT rats were treated with SIT (10 mg/kg/d) by intragastric administration for another 8 weeks. The body mass, vascular tension, serum oxidative stress indices and inflammatory parameters, pathological changes, protein expression of endothelial nitric oxide synthase (eNOS), the genes associated with ER stress and apoptosis in the thoracic aorta were measured. Furthermore, cell proliferation, ROS and the protein expression associated with ER stress (especially CHOP) and apoptosis were assessed in human umbilical vein endothelial cells (HUVECs) incubated with SIT and PA. Compared to the SCD rats, the HFD rats had higher serum lipid levels, decreased vascular tension, increased inflammation, oxidative and ER stress, and apoptosis of endothelial cells. PA promoted ROS generation, ER stress and apoptosis, inhibited cell proliferation in HUVECs. SIT treatment obviously ameliorated apoptosis via alleviating ROS and ER stress in the thoracic aortas isolated from HFD-fed rats and PA-treated HUVECs. The results suggest that SIT improved endothelial function via promoting cell proliferation and alleviating ROS-ER stress-CHOP pathway both *in vivo* and *in vitro*.

## Introduction

Metabolic syndrome (MS), a cluster of metabolic abnormalities associated with insulin resistance and abdominal obesity, is related to increased risks of type 2 diabetes mellitus (T2DM) and cardiovascular disease (CVD) (Eva et al., 2011). Obesity, as a worldwide health problem, has attracted academic attention increasingly. High-fat diet (HFD)-induced obesity significantly increases the risk for MS. Macrovascular disease is one of the most common determiners of morbidity and mortality in obesity-induced MS (Koumaras et al., 2013). However, the specific mechanisms in the development of macrovascular lesions have not been completely elucidated until now.

The endothelium plays a crucial role in maintaining vascular integrity and function. Endothelial dysfunction (ED), characterized by impaired nitric oxide (NO) release and increased endothelin-1 (ET-1) release, is fundamental in the early stage of CVD. Chronic exposure to high fat or high free saturated fatty acid drives inflammation, oxidative and endoplasmic reticulum (ER) stress in the endothelium (Lu et al., 2013). Endothelial cells (ECs) are distributed in the innermost layer of the endothelium, so damage to ECs is commonly thought to be the initial step of ED.

Dipeptidyl peptidase-4 (DPP4) is a ubiquitous enzyme and is detectable in numerous tissues including endothelium, which degrades incretins such as glucagon-like peptide-1 (GLP-1) and causes diabetes in humans. Inhibition of DPP-4 is a novel therapeutic option to raise the concentration of active GLP-1 and extend its activity to improve hyperglycemia by stimulating glucose-induced pancreatic insulin secretion while inhibiting glucagon production (Mulvihill and Drucker., 2014).

Alternatively, the role of DPP-4 in diabetes is not restricted to its GLP-1 degrading properties. DPP4 can also interact with proinflammatory signaling pathways in endothelium and causes ED. Mice lacking DPP4 are protected against obesity and insulin resistance (Conarello et al., 2003). Emerging evidence suggests that DPP-4 inhibitors exert the protective effects on the vascular endothelium (Aroor et al., 2014; Xu et al., 2017).

Mason et al. (2011) demonstrated that administration of obese Zucker rats with saxagliptin, one of several DPP-4 inhibitors, for 8 weeks, increased production of Ca^2+^ ionophore-induced NO and reduced the production of peroxynitrite in the aorta and glomerular endothelial cells. Saxagliptin could also exert both antioxidant and anti-inflammatory effects by reducing the number of plasma sCD40 cells (Mason et al., 2011). Des-fluoro-sitagliptin, one analogue of sitagliptin (SIT), enhanced endothelium-dependent vasodilatation (EDV) responses to acetylcholine (ACh) in arterial rings of mouse (Matsubara et al., 2012). Vildagliptin, also one of DPP-4 inhibitors, improved endothelial function by promoting forearm blood flow during ACh infusion in T2DM patients (Van Poppel et al., 2011). All these studies collectively support the concept that DPP-4 inhibitors have direct vascular protective effects independent of their glucose-lowering actions.

SIT, one orally stable effective and selective DPP-4 inhibitor (one tablet a day for T2DM), has been manifested effective control on blood sugar, as well as on mass and function of the islets, and no significant side effects (Aston-Mourney et al., 2013). Lu et al. observed that SIT therapy for 28 days reduces the thickness of the arteries, expression/activities of MMP-2 and MMP-9, vascular infiltration of the macrophages and apoptosis in normal glycemic ApoE^-/-^ mice infused with angiotensin II (Ang II), which indicated that SIT improves vascular remodeling by reducing inflammation in blood vessels (Lu HY. et al., 2015). The anti-inflammatory, anti-oxidative, and anti-apoptotic properties of SIT were also documented in the other literatures (Nader, 2014; Hu et al., 2017).

Recently, one research reported that SIT prevents activation of the pro-apoptotic calcium-dependent enzyme, calpain, induced by cytokine and endoplasmic reticulum (ER) stress, and partly suppress beta cell death in INS1E cells and human primary islets (Clark et al., 2017). SIT therapy can also effectively reduce fatty acid intake, decrease expression of VLDL receptor and triglyceride (TG) content in liver by attenuating MCD diet-induced hepatic inflammation, ER stress, and hepatic injury (Jung et al., 2014). However, the mechanisms enabling SIT to exhibit resistance to diet-induced obesity (DIO) related with ER stress in aorta and ECs have not been reported.

We hypothesize that SIT administration can enhance EDV responses to ACh and improve ED by alleviating oxidant, ER stress, and apoptosis in ECs induced by HFD and high concentration of saturated fatty acid. Palmitate (PA) is reported to be the main saturated fatty acid in the regular HFD; hence it was used to incubate with human umbilical vein endothelial cells (HUVECs) to unravel the molecular mechanisms of SIT’s vascular protection *in vitro*.

## Materials and Methods

### Experimental Animals and Protocols

Eight-week-old male Sprague Dawley (SD) rats of clean grade were obtained from the Experimental Animal Center of Anhui Medical University. All experimental procedures were approved by the Ethics Committee of Anhui Medical University. The rats were housed in plastic cages, allowed free access to fodder and water under normal light and dark cycle. After being acclimated for 1 week, the rats were randomly divided into three groups: standard chow diet (SCD), high-fat diet (HFD) and SIT administration group (HFD + SIT, Merck Sharp and Dohme, Australia). The rats in the HFD and HFD + SIT groups were fed a HFD (composed of 55% normal chow, 12% lard, 10% hen eggs, 8% milk powder, 5% cane sugar, 5% peanut, 3% sesame oil, and 2% common salt) for 12 weeks, then the HFD + SIT group were treated with SIT (10 mg/kg/d) via gavage for another 8 weeks. Body mass of the three groups were measured once a week from the 13th weeks of HFD feeding until the end of the animal experiment.

### Serological Analysis

After the rats were fasted and anesthetized, blood was obtained and the serum was separated to analyze levels of TG, total cholesterol (TC), high-density lipoprotein-cholesterol (HDL-C) and low-density lipoprotein-cholesterol (LDL-C) in an automatic biochemical analyzer (Roche, cobas 8,000, Germany). Serum interleukin 1β (IL1β) levels were measured with the radioimmunoassay kit (Beijing North Institute of Biological Technology, China). Serum free fatty acid (FFA, A042-1), Total Antioxidant Capacity (T-AOC, A015-1), superoxide dismutase (SOD, A001-1), Malondialdehyde (MDA, A003-1), and NO (A012-1) levels were analyzed with the commercial kits from Nanjing Jiancheng Bioengineering Institute (China). Serum ET-1(CSB-E06979r) and TNFα (CSB-E11987r) levels were analyzed with ELISA kits (CUSABIO, China).

### Isometric Tension Measurement of Aortic Rings

After blood was collected, intact thoracic aortas from every group were rapidly dissected and placed in ice-cold Krebs-Henseleit solution (118 mmol/L NaCl, 4.7 mmol/L KCl, 2.5 mmol/L CaCl_2_, 1.2 mmol/L KH_2_PO_4_, 1.2 mmol/L MgSO_4_·7H_2_O, 25.2 mmol/L NaHCO_3_, and 11.1 mmol/L glucose) for EDV analysis as before (Lu Y. et al., 2015). After the thoracic aorta was cut into ring segments, they were placed in a Biological Function Experimental System (BL-420E, TECHMAN, China), and passive tension of 1 g was applied to obtain the optimal resting tension. After an equilibrium period of 45 min, we used a high-K^+^ solution (58 mmol/L NaCl, 64.7 mmol/L KCl, 2.5 mmol/L CaCl_2_, 1.2 mmol/L KH_2_PO_4_, 1.2 mmol/L MgSO_4_·7H_2_O, 25.2 mmol/L NaHCO_3_, and 11.1 mmol/L glucose) to contract the vessel rings for 3 times. After washout, the rings were challenged with 10^–6^ mol/L Phenylephrine hydrochloride (PE, Aladdin, China) followed by cumulative concentrations of ACh (Sigma, United States) from 10^−8^ mol/L to 10^−4^ mol/L. The recorded EDV curves were plotted as percentages of the vasoconstriction values induced by PE.

### Pathological Examinations in Thoracic Aorta

Intact thoracic aortas from every group were either frozen in −80°C or fixed for analyzing the pathological or molecular changes. Oil-red O staining was used to analyze changes of distribution of neutral lipid in the frozen thoracic aorta according to the literature (Mahmoud et al., 2017). The fresh thoracic aorta was fixed in 10% formalin, embedded in paraffin, and cut into thickness of 5 μm. Then the sections were analyzed with HE staining, Masson trichrome staining (for collagen fibers and extracellular matrix) (Lee et al., 2012), immunohistochemical staining of CD68 expression (for macrophage-like cells) and Terminal Deoxynucleotidyl Transferase-Mediated dUTP-X Nick End Labeling (TUNEL) assay with the *In situ* Cell Death kit (Roche, 11684795910), respectively. The stained figures were photographed with Nikon Eclipse 80i fluorescence microscope (Japan). Quantification of CD68 and TUNEL positive staining were analyzed by Image J Software for three representative images from each group.

### RNA Isolation and Semi-Quantitative Reverse Transcription-PCR

RNAiso Plus reagent (Takara, 9,108) was used to extract total RNA from the frozen thoracic aorta. 2 μg of the total RNA was reverse transcribed into cDNA using PrimeScript™ 1^st^ Strand cDNA synthesis kit (Takara, 6,110). Then the cDNAs were subjected to sqRT-PCR analysis. Carnitine palmitoyl transferase 1b (CPT1b) and acetyl-CoA carboxylase (ACC) participated in fatty acid metabolism, meanwhile C/EBP homologous protein (CHOP) were selected as effector of activation of ER stress. The cycling condition was: 95°C 30 s, 55°C 10 s, 72°C 10 s for total 32 cycles in TC-512 (TECHNE, Britain), then the PCR products were analyzed on a 3% agarose gel. The mRNA levels of target genes were normalized by being compared with GAPDH expression. Each experiment was repeated three times and quantified by Image J software. Primers were designed and synthesized by Shanghai Sangon Biotechnology Company (China) and their sequences were as following:

CPT1b

Sense 5′-CCA​AAC​ATC​ACT​GCC​CAA​GC-3′

Antisense 5′-CGA​CTC​CAT​GCG​GAA​ATA​GG--3′

ACC

Sense 5′-AAC​CAG​CAC​TCC​GGA​TTC-3′

Antisense 5′-AGG​CCA​AAC​CAT​CCT​GTA​A-3′

CHOP

Sense 5′-TGG​AAG​CCT​GGT​ATG​AGG​ATC​TG-3′

Antisense 5′-GAG​GTG​CTT​GTG​ACC​TCT​GCT​G-3′

GAPDH

Sense 5′-GGC​ACA​GTC​AAG​GCT​GAG​AAT​G-3′

Antisense 5′-ATG​GTG​GTG​AAG​ACG​CCA​GTA-3′

### Cell Culture and Colorimetric MTT Assay

HUVECs were obtained from the American Type Culture Collection (ATCC, Manassas, United States) and cultured in F12K medium (Boster, Wuhan, China) supplemented with 10% FBS, 100 U/ml penicillin-streptomycin in a humidified incubator at 37°C and 5% CO_2_. PA (Sigma, United States) was coupled to fatty acid-free BSA in the ratio of 2:1 (PA: albumin) before cell experiment, the concentration of PA in the cell experiment was referred as 0.5 mmol/L according to the previous literature (Lu Y. et al., 2015). A modified colorimetric MTT assay was used to choose suitable SIT concentrations (Solarbio, China, 20, 40, 80, 160, 320 μmol/L) to eliminate toxicity of PA. Briefly, the logarithmic cells were prepared into single cell suspension and counted with a cell counter to adjust the number of the cell suspension. Afterwards, the cells were divided into CON, PA and different PA + SIT groups (5 parallels for each group). 1 × 10^4^ cells were inoculated into each well and incubated for 24 h, synchronized for 12 h by serum starvation, then treated with SIT or PA for 24 h. After 20 µL MTT was added into the medium for 4 h, the medium was discarded and 150 µL DMSO was added into each well to dissolve formazan in a microplate oscillator for 20 min. The absorbance values were measured at 490 nm with a multimode reader, and the cell survival rates were calculated and compared between different groups. Finally, two dosages of SIT (40 and 160 μmol/L) were selected as the low dosage (SIT-L) and the high dosage (SIT-H) respectively in the subsequent cell experiment.

### Intracellular ROS Assay in HUVECs

Changes in intracellular ROS levels were determined by measuring the oxidative conversion of cell permeable 2’,7’-dichlorofluorescein diacetate (DCFH-DA) to fluorescent dichlorofluorescein (DCF) using ROS detection kit (Beyotime, S0033, China). Tunicamycin (TM) and 4-phenyl butyric acid (PBA) were selected as positive and negative controls of ER stress *in vitro*. The cells were allowed to grow into near confluency, synchronized for 12 h by serum starvation and divided into eight groups: NC (reagents control), Rosup (positive control), CON (no palmitate), PA, PA + SIT-L, PA + SIT-H, PA + PBA (PA+10 mmol/L PBA), TM (1 μg/ml) for 24 h respectively. The cells were washed with D-Hank’s and incubated with DCFH-DA at 37°C for 20 min. Then DCF fluorescence were photographed by Nikon Eclipse 80i fluorescence microscope (Japan) at an excitation wavelength of 488 nm and at an emission wavelength of 525 nm. Finally, quantification of the images was analyzed by Image J Software for three representative images from each group.

### Cell Cycle Assay in HUVECs

The cells were divided into six groups: CON (no palmitate), PA, PA + SIT-L, PA + SIT-H, PA + PBA, or TM for 24 h, respectively, then cells were harvested by trypsin, centrifuged at 1,000×g for 5 min, resuspended in ice-cold PBS, and fixed in 70% ethanol at 4°C for 18 h. The fixed cells were washed again using ice-cold PBS and incubated with 500 μl propidium iodide containing 0.05% RNase A for 30 min at room temperature in the dark. Finally, a cell cycle distribution profile was detected on BD FACS Verse flow cytometer (United States) at the excitation wavelength of 488 nm. The cell cycle assay kit was from Beyotime Institute of Biotechnology (China). The percentages of cells in G_0_/G_1_, S, and G_2_/M phases were analyzed with Flowjo 7.6 software. The experiment was repeated three times.

### Immunofluorescence Assay of CHOP in HUVECs

CHOP is the typical apoptosis marker in ER stress, its location and expression in the PA/SIT-treated HUVECs were observed using immunofluorescence assay. The cells were divided into six groups: CON (no palmitate), PA, PA + SIT-L, PA + SIT-H, PA + PBA, or TM for 24 h, respectively, HUVECs were fixed in 4% paraformaldehyde solution for 15 min. After the nonspecific binding sites were blocked with 1% BSA, the fixed cells were incubated with CHOP mouse monoclonal antibody (L63F7, #2895, CST, 1:1,000) overnight at 4°C, followed by incubation with goat anti-mouse FITC-conjugated secondary antibody (Beijing Zhongshan Golden Bridge, China) and DAPI, covered with antifade mounting medium. The edges of the coverslip were sealed with nail polish. Ultimately the stained cells were photographed with Nikon Eclipse 80i fluorescence microscope (Japan). Quantification of the images was analyzed by Image J Software for three representative images from each group.

### Western Blotting

Total protein was extracted from the frozen thoracic aorta of rats and HUVECs with RIPA lysis buffer containing protease inhibitor cocktail. The protein concentrations were determined by the Bradford protein assay kit. Equal amounts of protein lysates were separated by SDS-PAGE and transferred to polyvinylidene difluoride membranes followed by a block with 5% skimmed milk at 4°C for 1.5 h. Subsequently, the membranes were incubated with primary antibodies against eNOS (AF0096, Affinity, 1:1,000) and p-eNOS (AF3247,Affinity, 1:1,000), c-jun N-terminal kinase (JNK BS1544, Bioworld, 1:500) and p-JNK (BS4322, Bioworld, 1:500), B-cell lymphoma-2 (Bcl-2, BA0412, Boster, 1:800), BCL2-Associated X (Bax, BA0315-2, Boster, 1:800), inositol-requiring enzyme 1α (IRE1α, bs-8680R, Bioss, 1:1,000), glucose regulated protein 78 (GRP78, ab21685, Abcam, 1:1,000), CHOP, respectively. *β*-actin (M02014-5, Boster, 1:1,500) was used as the internal reference. Antibody binding was detected with enhanced chemiluminescent agent and quantified with Image J software. Each experiment was repeated three times.

### Statistical Analysis

All data are expressed as mean ± SEM values. SPSS software 23.0 (IBM Corporation, United States) was selected to analyze statistical significance of data. Unpaired 2-tailed Student’s t-tests were used to compare between two experimental groups as well as one-way analysis of variance (ANOVA) followed by Bonferroni’s *post hoc* test were employed to assess significant differences among multiple groups. A two-tailed *p*-value of less than 0.05 was considered statistically significant.

## Results

### SIT Reduced Body Weight and Improved Vascular Tension of the HFD-Fed Obese Rats

**FIGURE 1:**
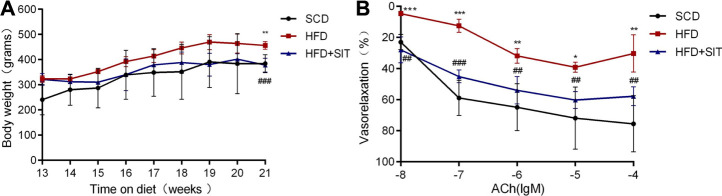
**Influence of Sitagliptin on body weight and vasodilatation of thoracic aorta from the HFD-fed rats. (A)** body weight, values were expressed as mean ± SEM. *n* = 5 in each group; **(B)** endothelium-dependent vasodilatation of thoracic aortas. Values were expressed as mean ± SEM. *n* = 4 in each group. SCD: standard chow diet; HFD: high-fat diet; HFD + SIT: HFD and sitagliptin therapy group. *p < 0.05,**p < 0.01,***p < 0.001 vs. SCD group; ^##^ *p* < 0.01, ^###^ *p* < 0.001 vs. HFD group.

At the beginning of the experiment, all male rats have similar body weight. 12 weeks of HFD fodder caused more body mass in HFD and HFD + SIT groups, while 8 weeks of SIT administration (10 mg/kg/d) remarkably reduced body mass as compared with HFD group (Figure 1A). In the vascular tension experiment, EDV reaction to ACh were evidently diminished in the aortic rings isolated from the HFD-fed rats. Meanwhile, EDV reaction to ACh were improved in the aortic rings isolated from the HFD + SIT group (Figure 1B).

### SIT Improved Serum Lipid, Inflammation, and Oxidative Stress of the HFD-Fed Obese Rats

**FIGURE 2:**
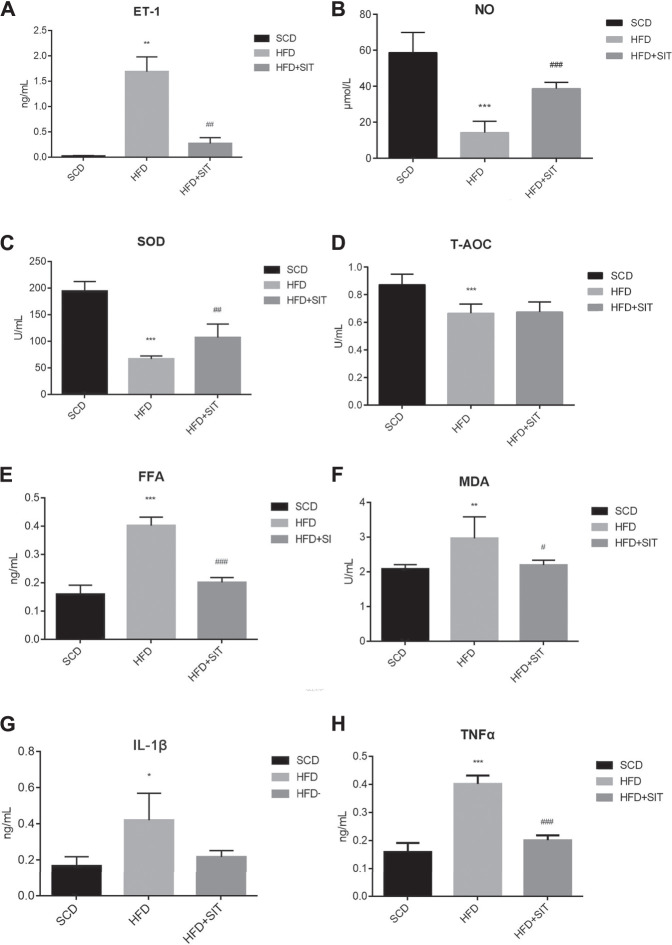
**Influence of sitagliptin on serum indices in the HFD-fed rats. (A)** serum ET-1 levels, *n* = 3 in each group; **(B)** serum NO levels, *n* = 6 in each group; **(C)** serum SOD levels, *n* = 6 in each group; **(D)** serum T-AOC levels, *n* = 6 in each group; **(E)** serum FFA levels, *n* = 6 in each group; **(F)** serum MDA levels, *n* = 6 in each group; **(G)** serum IL-1β levels, *n* = 3 in each group; **(H)** serum TNFα levels, *n* = 3 in each group. SCD: standard chow diet; HFD: high-fat diet; HFD + SIT: HFD and sitagliptin therapy group. All values displayed are mean ± SEM.*p < 0.05, **p < 0.01,***p < 0.001 vs. SCD group; ^#^ *p* < 0.05, ^##^ *p* < 0.01, ^###^ *p* < 0.001 vs. HFD group.

Serum TG, TC, and LDL-C levels were remarkably increased in the HFD group, and the HDL-C levels were significantly decreased in the HFD group. SIT administration in HFD + SIT group reversed all above changes (Supplementary Table S1). When compared with the SCD group, the serum levels of ET-1, FFA, MDA, IL-1β, and TNFα in the HFD group were elevated significantly accompanied with the reduction of serum levels of T-AOC, SOD and NO. Compared to the HFD group, the levels of serum ET-1, FFA, MDA, and TNFα in HFD + SIT group were decreased remarkably, but the levels of serum IL-1β between the two groups showed no significant difference. At the same time, SIT treatment significantly elevated the serum SOD and NO levels, while the serum T-AOC levels had no significant difference between HFD and HFD + SIT groups (Figures 2A–H).

### SIT Improved HFD-Induced Pathological Changes, Lipid Deposition and Apoptosis of Endothelial Cells in the Thoracic Aorta

**FIGURE 3:**
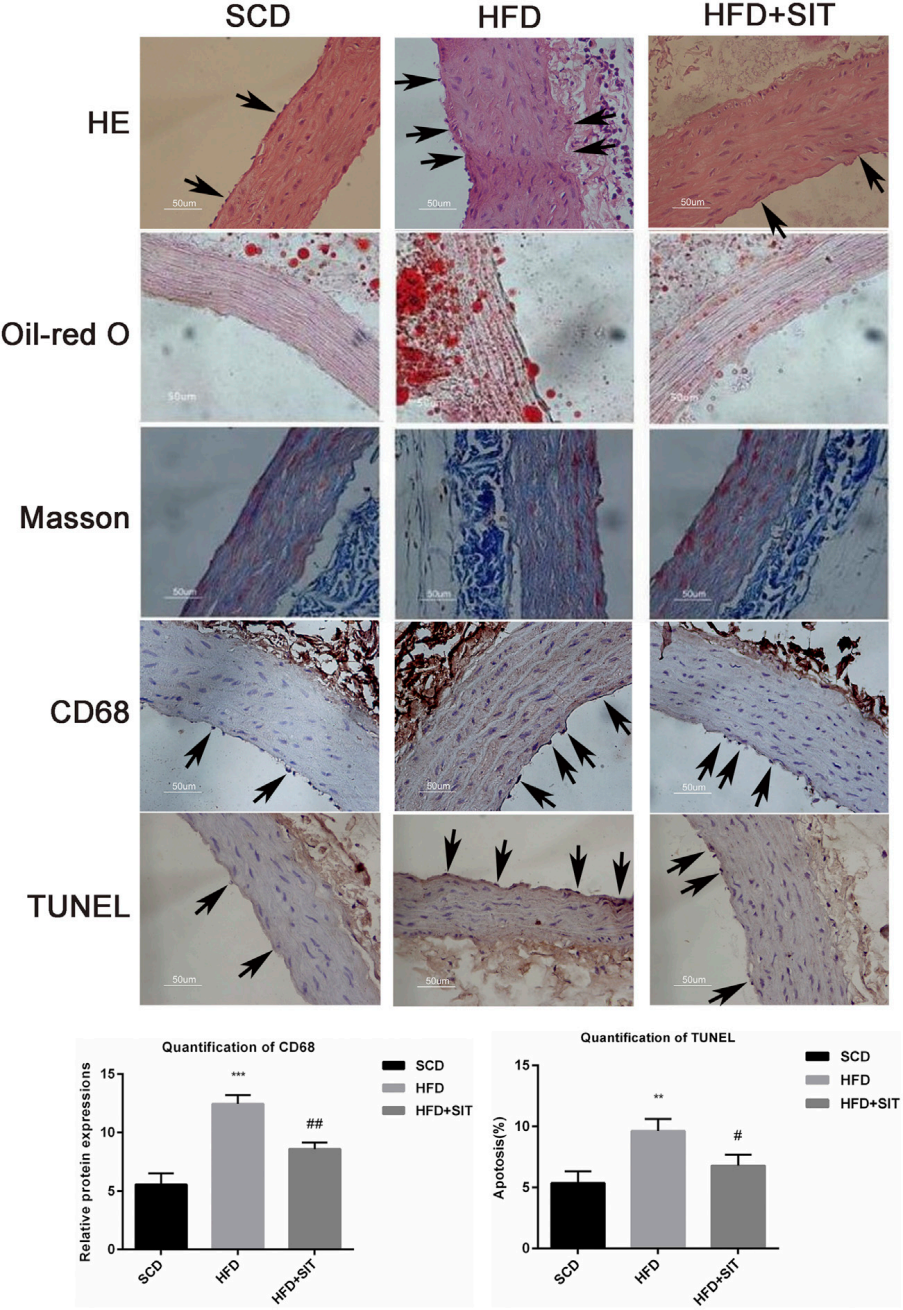
**Effects of sitagliptin on the pathological changes in the thoracic aorta from the HFD-fed rats.** Pathological changes in the thoracic aorta were evaluated with HE staining; Oil-red O staining; Masson trichrome staining; immunohistochemical staining of CD68 and TUNEL staining respectively. Representative photographs were displayed here. scale bars = 50 μm (×400). SCD: standard chow diet; HFD: high-fat diet; HFD + SIT: HFD and SIT therapy group. Typical changes are marked with black arrows in the images of HE, CD68 and TUNEL staining. Quantification of CD68 and TUNEL staining were also shown, values displayed are mean ± SEM from three representative images.**p < 0.01,***p < 0001 vs. SCD group; ^#^ *p* < 0.05,^##^ *p* < 0.01 vs. HFD group.

HE staining showed pathological changes, including irregular thickening of the thoracic aortic wall as well as disorders and fractures of elastic fibres in the HFD-fed rats (indicated by arrows in Figure 3). Oil red-O staining indicated that more lipids were deposited in the vascular walls of the HFD group (Figure 3), which was consistent with elevated serum levels of TG, TC, and LDL-C (Supplementary Table S1). Masson trichrome staining indicated the reduction of elastic fibers and the increase of collagen fibers in the thoracic aorta of HFD-fed rats. Immunohistochemistry revealed that CD68 positive cells (representative as macrophage-like cells) increased abnormally in the thoracic aorta from the HFD group. The TUNEL assay indicated that the increased apoptotic nuclei were distributed mainly in aortic endothelial cells of the HFD group (the apoptotic endothelial cells were indicated by the arrows). Compared to the HFD rats, HFD + SIT rats displayed the improvements of pathological changes, lipid deposition, macrophage accumulation, and apoptosis of the aortic endothelial cells, Quantification of CD68 and TUNEL staining supported the above results (Figure 3).

### Effect of SIT on Gene Expression Associated With TG Metabolism, ER Stress and Apoptosis in the Thoracic Aorta

**FIGURE 4:**
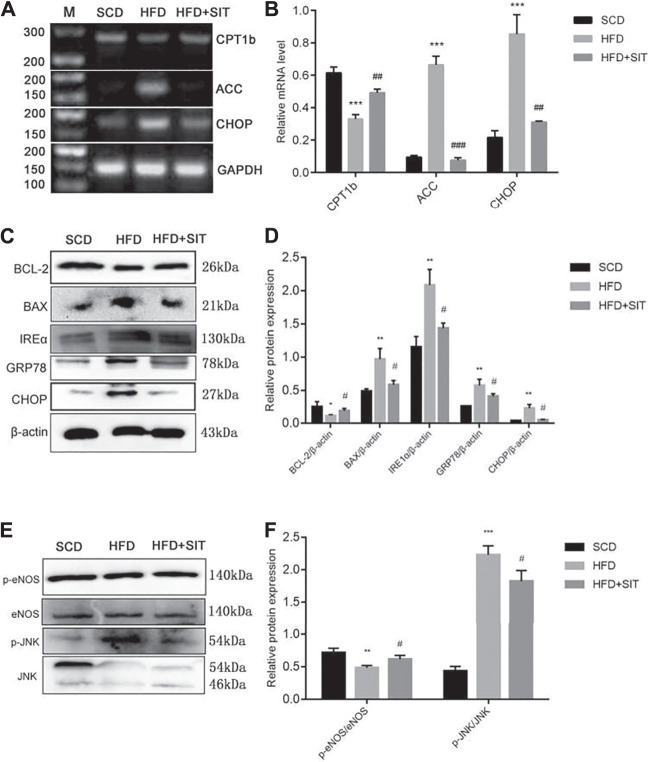
**Effects of Sitagliptin on gene expression in the thoracic aortas from the HFD-fed rats. (A)** sqRT-PCR results of mRNA expression of CPT1b, ACC and CHOP; **(B)** Quantification analysis of A; **(C)** Western blotting results of BCL-2, BAX, IRE1α, GRP78, and CHOP; **(D)** Quantification analysis of C; **(E)** Western blotting results of p-eNOS, eNOS, p-JNK, JNK; **(F)** Quantification analysis of E. All values displayed are means ± SEM of three independent experiments. *p < 0.05, **p < 0.01,***p < 0001 vs. SCD group; ^#^ *p* < 0.05,^##^ *p* < 0.01 vs. HFD group.

Compared to the SCD group, the HFD group displayed decreased CPT1b and increased ACC mRNA expression, while SIT treatment significantly reversed ACC and CPT1B mRNA expression in the aorta from the HFD + SIT group (Figures 4A,B). HFD also induced ER stress indicated by increasing CHOP mRNA expression, whereas SIT administration eased HFD-induced CHOP expression in thoracic aorta. Protein expression of IRE1α, CHOP, and GRP78 also increased in the HFD group. Meanwhile, SIT therapy mitigated ER stress by decreasing IRE1α, CHOP, and GRP78 expression (Figures 4C,D). Moreover, we analyzed p-eNOS/eNOS expression and discovered that the ratio of p-eNOS/eNOS decreased obviously in the HFD group, meanwhile, SIT administration significantly increased the ratio of p-eNOS/eNOS (Figures 4E,F). Change of p-JNK/JNK expression was exhibited as the same as protein expression of IRE1α, GRP78, and CHOP, which suggested that JNK was also the downstream molecule of ER stress and activated by HFD fodder, while SIT administration reversed p-JNK expression (Figures 4E,F).

Western blot analysis also showed that the protein expression of BAX increased along with decreased protein expression of BCL-2 in the thoracic aorta of the HFD-fed rats, while SIT alleviated HFD-induced apoptosis by reversing the protein expresssions of BCL-2 and BAX (Figures 4C,D), which was consistent with the TUNEL staining results in Figure 3.

### Effect of SIT on Intracellular ROS Levels and Cell Cycles in PA-Treated HUVECs

PA is known for inducing oxidative stress (Mahmoud et al., 2017). SIT administration alleviated oxidative stress via decreasing serum MDA levels and increasing serum SOD levels in the HFD-fed rats (Figures 2C,F), so the effect of SIT on intracellular ROS production in PA-induced HUVECs was investigated.

**FIGURE 5:**
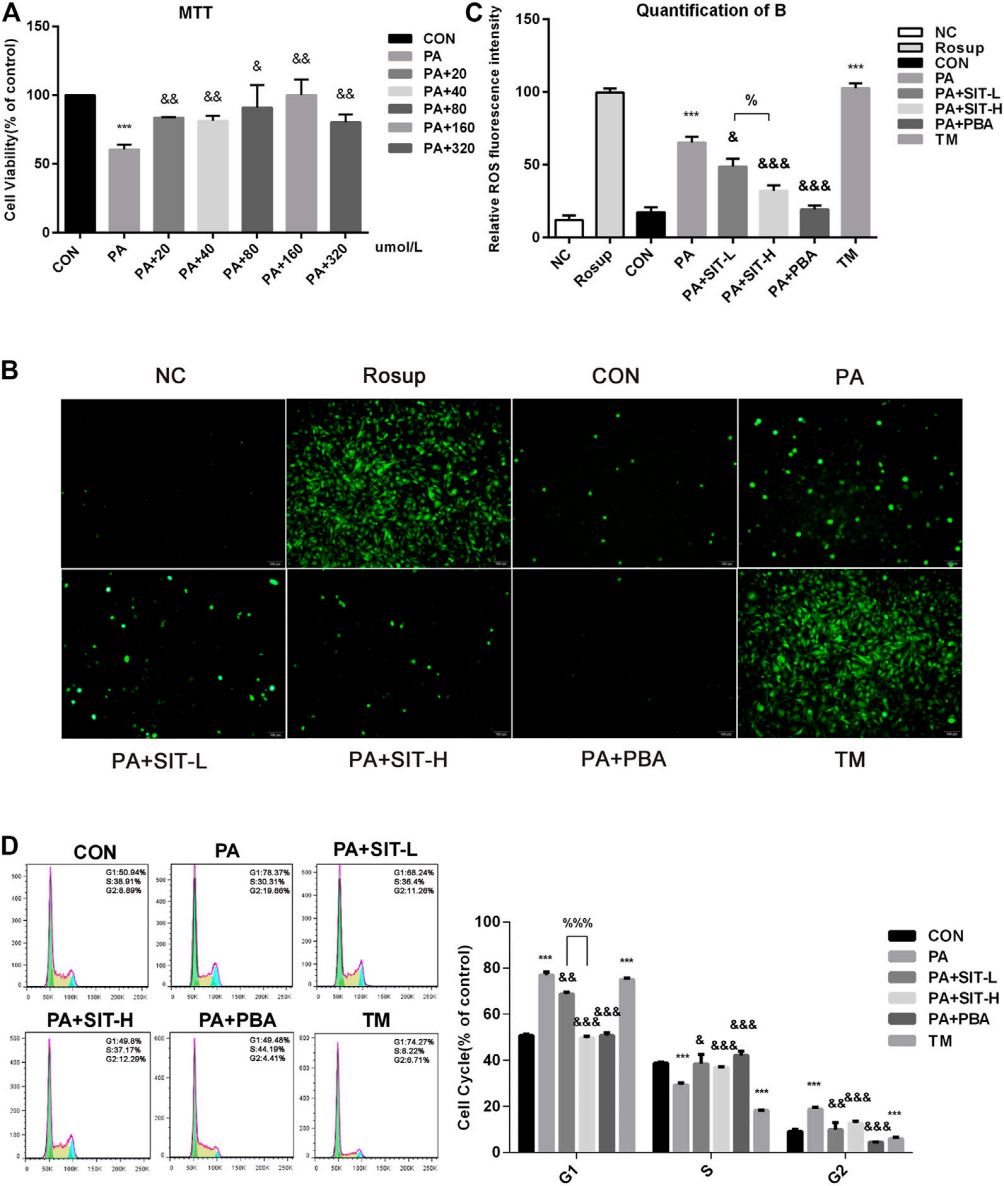
**Effects of Sitagliptin on ROS level and cell cycles in PA-treated HUVECs. (A)** MTT assay: HUVECs were treated with CON (no PA medium), PA (0.5 mmol/L PA), PA + 20 (PA + 20 μmol/L SIT), PA + 40 (PA + 40 μmol/L SIT), PA + 80 (PA + 80 μmol/L SIT), PA + 160 (PA + 160 μmol/L SIT), and PA + 320 (PA + 320 μmol/L SIT) for 24 h respectively; **(B)** Assay of ROS level in HUVECs. HUVECs were treated with NC, Rousp, CON (no PA medium), PA, PA + SIT-L (PA + 40 μmol/L SIT); PA + SIT-H (PA + 160 μmol/L SIT); PA + PBA (PA + 10 mmol/L PBA) and TM (1 μg/ml) for 24 h respectively; **(C)** Quantification of B, values displayed are mean ± SEM from three representative images; **(D)** Cell proliferation of HUVECs by flow cytometry: HUVECs were treated with CON, PA, PA + SIT-L, PA + SIT-H, PA + PBA and TM for 24 h respectively, values displayed are means ± SEM of three independent experiments. ***p < 0.001 vs. CON group; ^&&^ *p* < 0.01, ^&&&^ *p* < 0.001 vs. PA group; ^%^ *p* < 0.05, ^%%%^ *p* < 0.001 vs. SIT-L group.

Essentially, two different concentrations of SIT (40 and 160 μmol/L) were selected to represent as the SIT-L and SIT-H groups, according to the results of MTT colorimetric assay (Figure 5A). The results indicated that ROS generation increased obviously after PA incubation, whereas SIT administration decreased the ROS levels in a concentration-dependent manner as compared with the separate PA incubation, while TM treatment increased obviously ROS levels. Quantification of ROS confirmed the above conclusion (Figures 5B,C). Cell cycle experiment showed that PA or TM treatment promoted HUVECs to stagnate in G1 and G2 phases and prevented them from entering the S phase, whereas SIT and PBA administration reversed the cell cycle in PA-treated HUVECs (Figure 5D), which suggested that some unknown molecules in ER stress were potentially related to the control of cell cycle.

### Effect of SIT on Intracellular CHOP Distribution and Expression in PA-Treated HUVECs

**FIGURE 6:**
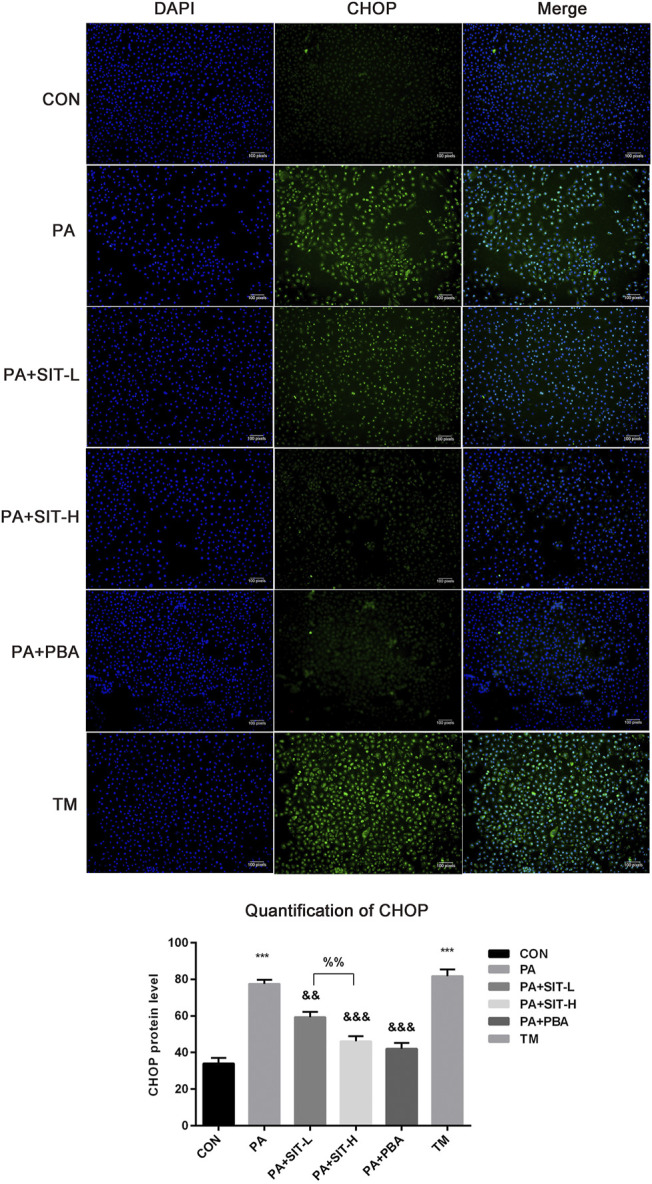
**Immunofluorescence assay of CHOP expression and distribution in PA + SIT-treated HUVECs.** HUVECs were incubated with CON, PA, PA + SIT-L, PA + SIT-H, PA + PBA and TM for 24 h, respectively. Immunostaining for CHOP (green) and DAPI (blue), merged figures for both images. All values displayed are means ± SEM of three representative images.***p < 0.001 vs. CON group; ^&&^ *p* < 0.01, ^&&&^ *p* < 0.001 vs. PA group; ^%%^ *p* < 0.01 vs. SIT-L group.

CHOP is a typical proapoptotic transcription factor in ER stress (Lee et al., 2012), so immunofluorescence assay was used to visually detect the expression and distribution of CHOP in HUVECs, with DAPI labeling the nucleus. As shown in Figure 6, CHOP was expressed and mainly localized in the nucleus in PA or TM treatment group, whereas SIT administration decreased the protein expression of CHOP in PA-treated HUVECs and there was significant difference between the SIT-L and SIT-H groups, which suggested a concentration-dependent protective effect. Quantification of ROS confirmed the above results.

### Effect of SIT on Gene Protein Expression in PA-Treated HUVECs

**FIGURE 7:**
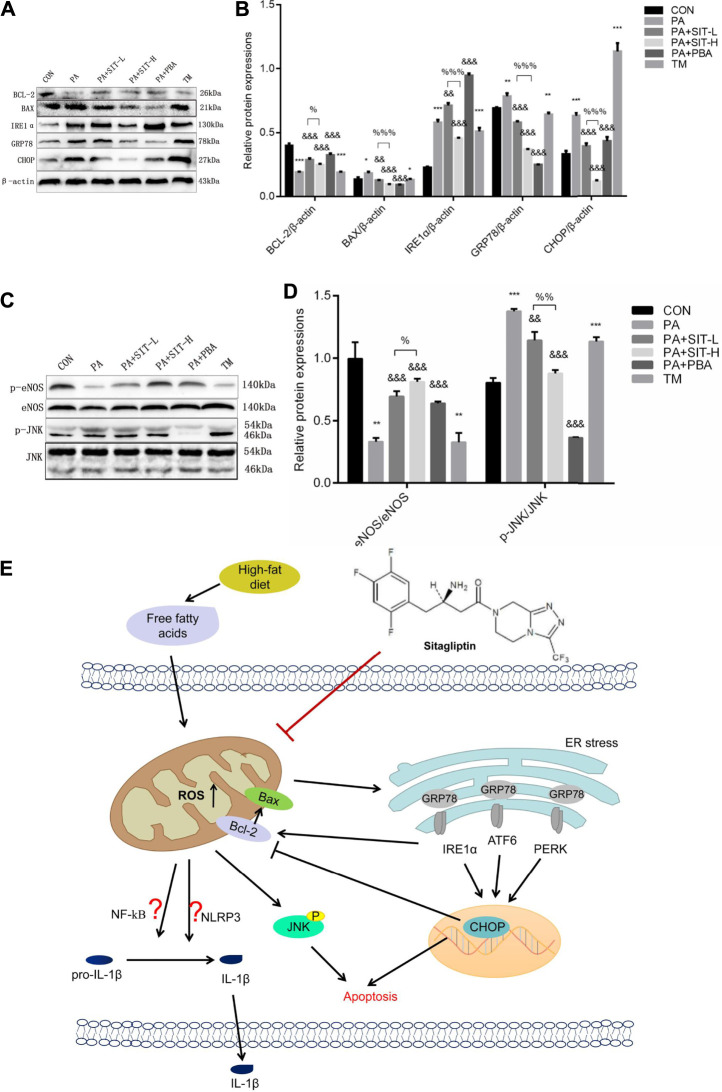
**Effects of sitagliptin on protein expression in PA-treated HUVECs and summary of the main findings in this study. (A)** Western blotting results of BCL-2, BAX, IRE1α, GRP78, and CHOP; **(B)** Quantification analysis of A; **(C)** Western blotting results of p-eNOS, eNOS, p-JNK and JNK; **(D)** Quantification analysis of C. All values displayed are means ± SEM of three independent experiments. *p < 0.05, **p < 0.01, ***p < 0.001 vs. CON group; ^&&^ *p* < 0.01, ^&&&^ *p* < 0.001 vs. PA group; ^%^ *p* < 0.05, ^%%^ *p* < 0.01 vs. SIT-L group. **(E)** Proposed mechanism of sitagliptin attenuating HFD/PA-induced endothelial dysfunction and apoptosis possibly by ROS-ER stress-CHOP pathway.

The effects of SIT on protein expression in PA-treated HUVECs were analyzed by Western blot. The protein expressions of BAX, IRE1α, GRP78, and CHOP were increased while the protein expression of BCL-2 was decreased in PA or TM-treated HUVECs. Whereas SIT or PBA reversed the above protein expressions and SIT incubation showed a concentration-dependent effect (Figures 7A,B). PA or TM treatment also inhibited the protein expression of p-eNOS and promoted the protein expression of p-JNK, whereas SIT or PBA incubation reversed the protein expressions of p-eNOS and p-JNK, similarly SIT treatment showed a concentration-dependent effect (Figures 7C,D).

## Discussion

In this study, we reported for the first time that SIT, as the first approved DPP4 inhibitor by the FDA to treat T2DM and MS, attenuated HFD/PA-induced ED and endothelial injuries in the thoracic aorta and HUVECs. Furthermore, our study indicated that SIT had multiple effects, such as hypolipidemic, anti-oxidation, anti-inflammation, anti-apoptosis, and promoting cell proliferation by ameliorating ROS-ER stress-CHOP pathway *in vivo* and *in vitro* (summarized in Figure 7E).

Firstly, SIT therapy showed obvious lipid-lowering effect by decreasing body weight, serum lipid levels and lipid deposition on the vascular wall, which potentially related to promoting fatty acid oxidation (CPT1b) and inhibiting TG synthesis (ACC) in the thoracic aorta. In addition to glycemic control, some other DPP-4 inhibitors also showed beneficial effects on lipid metabolism in basic and clinic research. Anagliptin exhibited a lipid-lowering effect in a hyperlipidemic animal model (male LDLR^-/-^ mice), the mechanism was related to downregulation of expression and activities of hepatic sterol regulatory element-binding protein in HepG2 cells (Yano et al., 2017). In one large cohort research, including 5,861 patients who received SIT treatment, the patients lost 1.1+/−5.39 kg on average (Horton et al., 2010), which supported our animal results.

Secondly, SIT therapy showed anti-oxidative effect in the HFD/PA-induced thoracic aorta and HUVECs. Our animal experiment showed that chronic HFD fodder for 20 weeks promoted lipid peroxidation (MDA) and reduced SOD and T-AOC levels in the serum, whereas SIT treatment reversed their changes. Mechanistically, SIT decreased ROS levels in a dose-dependent manner in PA-treated HUVECs. Linagliptin, a powerful DPP-4 inhibitor, exhibited more antioxidative capacity than SIT in white adipose tissue and liver (Zhuge et al., 2016).

Thirdly, SIT therapy showed anti-inflammatory effect in HFD/PA-induced thoracic aorta and HUVECs. TNFα and IL-1β are the main obesity-induced proinflammatory cytokine and chemokine, respectively. Our results indicated that SIT administration decreased serum IL-1β and TNFα levels, reduced macrophage infiltration in the thoracic aorta (CD68), and lessened p-JNK expression in HFD/PA-induced thoracic aorta and HUVECs. Recent research reported that SIT also ameliorated hypoxia/reoxygenation (H/R)-induced injuries by decreasing expression of IL-6, IL-8, and TNFα in cardiac microvascular ECs (Fan et al., 2019). SIT inhibited release of TNFα, IL-6 and IL-8 induced by LPS, therefore, it exerts anti-inflammatory effects in human lung microvascular ECs (Kawasaki et al., 2018), which was consistent with our results. Another research reported that SIT remarkably alleviated endothelial function and the inflammatory state in the patients with CAD plus uncontrolled DM, which strongly implied that SIT is beneficial to the cardiovascular system in DM patients (Matsubara et al., 2013).

Similarly, vildagliptin was able to limit inflammation by suppression of the NF-κB signaling pathway and proinflammatory agents such as TNF-α, IL-1β, and IL-8 (Wiciński et al., 2020). In another study, researchers investigated the molecular mechanism of vildagliptin in the protection of FFA-induced endothelial dysfunction. They demonstrated that vildagliptin suppressed FFA-induced expression of proteins of the NLRP3 inflammasome complex, including NLRP3, ASC, p20, and HMGB-1 (Qi et al., 2019).

Fourthly, SIT therapy showed anti-apoptotic effect (including TUNEL staining, Bcl-2, and BAX protein expression) in the HFD-induced aorta and in PA-treated HUVECs. SIT also significantly decreased CHOP mRNA and protein expression, which serves as an apoptotic modulator, meaning hyperactivation of ER stress. Until now, there has been limited reports on impact of SIT on ER stress (Bachor et al., 2015; Yuzbasioglu et al., 2018; Kizilay et al., 2021).

Finally, our results demonstrated that SIT significantly decreased expressions of IRE1α, GRP78, and CHOP in the aorta from the hyperlipidemic rats and in PA-treated HUVECs. SIT was effective in reducing apoptosis by decreasing the expression of caspase 3, caspase 12, GRP78, CHOP in the ER stress pathway (Kizilay et al., 2021).

SIT also effectively alleviated MCD diet-induced hepatic inflammation, ER stress, and liver injury, as evidenced by reduced proinflammatory cytokine levels, ER stress and apoptosis (Jung et al., 2014), which was similar to our results. In a recent study, vildagliptin reduced the stenosis of injured carotid arteries in db/db mice, and this protective effect was achieved by blocking the IRE-1 pathway and the expression of phospho-IKKα/β in vascular smooth muscle cells, so the mechanism was related to inhibition of ER stress/NF-κB pathway (Ji et al., 2019). Whether SIT is related with NF-κB or NLRP3 signaling pathway in HFD/PA-treated endothelial cells is unknown for now and needs further investigation.

Hyperglycemia, hyperinsulinemia, and dyslipidemia are the main factors involved in the pathogenesis of endothelial dysfunction. Compared with metformin and dapagliflozin, DPP-4 inhibitors have a neutral effect on endothelial function (Tentolouris et al., 2020). However, our results strongly suggested that, administration of SIT ameliorated HFD/PA-induced ED by improving EDV response, elevating serum NO level and reducing serum ET-1 level, accompanied with increased p-eNOS/NOS expression. Wang’s group also reported that SIT treatment can alleviate the endothelium-dependent relaxation and improve the endothelial injury of Zucker Diabetic fatty rats (Wang et al., 2018). Another research reported that SIT (50 mg/kg/d) caused a significant decrease in infarct size and DPP-4 activity, and an obvious increase in GLP-1 and NOS activity, expression of e-NOS (Al-Awar et al., 2018). Saxagliptin, in combination with metformin, can help improve endothelial dysfunction in early diabetes before macrovascular complications appear, and this effect was potentially related to upregulation in CD34^+^ endothelial progenitor cells for antioxidant SOD1 (Dore et al., 2018). The results of the above three reports all supported our results.

Remarkably, SIT treatment reduced a substantial amount of BrdU labeling in two different regions of the subventricular zone, thus protected proliferation of neural progenitor cells in diabetic mice (Bachor et al., 2015). Our findings also suggested that SIT administration in two doses promoted HUVECs to enter S phase, holding them back to stop at G1 and G2 phase, thus, promoted cell proliferation, but it exhibited no obvious dose-dependent effect. Combined with the results of SIT on ER stress, some unknown molecules in ER stress were potentially related to the control of cell cycle, which needs further investigation.

Recently, there has been one report about SIT genotoxic effects on human lymphocytes. SIT was used in the concentration range of 31.25–1,000 μg/ml, which was equivalently as 76.78–2,460 μmol/L. The results indicated that high concentration of SIT had genotoxic effects, indicated by increased mean comet tail intensity and tail moment in the human lymphocytes *in vitro* (Yuzbasioglu et al., 2018). Our SIT doses were of the concentrations of 40 and 160 μ mol/L therefore, the results mentioned above testified that SIT had no genotoxic effects on HUVECs in our experiment, which means that our results of cell research are credible.

The limitations of the current study are: 1) the levels of GLP-1 in serum and DDP-4 activity in the endothelium and HUVEC were not measured; 2) whether the effect of SIT on alleviating ER stress is through ATF6 or PERK pathway needs further investigation; 3) whether anti-inflammatory effects of SIT are related to NF-κB or NLRP3 signaling pathway needs further investigation; 4) whether PA and SIT treatment influence vascular endothelium repair or endothelial progenitor cell function is unknown and needs further investigation.

In conclusion, SIT can alleviate HFD/PA-induced endothelial dysfunction and apoptosis via ROS-ER stress-CHOP pathway in the endothelial cells. SIT has the potential as an agent to improve hyperlipidemia as well as lowering blood glucose.

## Data Availability

The raw data supporting the conclusions of this article will be made available by the authors, without undue reservation, to any qualified researcher.

## Glossary

ACC: acetyl-CoA carboxylase
ACh: acetylcholine
Ang II: angiotensin II
BAX: BCL2-Associated X
BCL-2: B-cell lymphoma-2
CHOP: C/EBP homologous protein;
CPT1b: Carnitine palmitoyl transferase 1b
CVD: cardiovascular disease
DIO: diet-induced obesity
DPP: -4Dipeptidyl peptidase-4
ED: Endothelial dysfunction
EDV: endothelium-dependent vasodilatation
eNOS: endothelial nitrc oxide synthase
ER: endoplasmic reticulum
ET-1: endothelin-1
FFA: free fatty acid
GLP-1: glucagon-like peptide-1
GRP78: glucose regulated protein 78
HDL-C: high density lipoprotein-cholesterol
HFD: high-fat diet
HFD + SIT: HFD plus sitagliptin administration
HUVECs: human umbilical vein endothelial cells
IL-1β: interleukin 1β
IRE1α: inositol-requiring enzyme 1alpha
JNK: c-jun N-terminal kinase
LDL-C: low density lipoprotein-cholesterol
MDA: Malondialdehyde
MS: Metabolic syndrome
NO: nitric oxide
PA: palmitate
PBA: 4-phenyl butyric acid
PE: phenylephrine hydrochloride
ROS: reactive oxygen species
SOD: superoxide dismutase
SCD: standard chow diet
SD: Sprague Dawley
SIT: sitagliptin
sqRT-PCR: semi-quantitative reverse transcription-PCR
T-AOC: Total Antioxidant Capacity
TC: total cholesterol
TG: triglyceride
TM: tunicamycin
TUNEL: Terminal Deoxynucleotidyl Transferase-Mediated dUTP-X Nick End Labeling
T2DM: type 2 diabetes mellitus
