# Supplementary material for: Sitagliptin Reduces Endothelial Dysfunction and Apoptosis Induced by High-Fat Diet and Palmitate in Thoracic Aortas and Endothelial Cells via ROS-ER Stress-CHOP Pathway
Source: Front Pharmacol. 2021 Aug 31;12:670389. doi: 10.3389/fphar.2021.670389 (PMC8438525; doi:10.3389/fphar.2021.670389)
Supplement: Supplementary file 1 [file Table1.DOCX]

**Table S1 Effect of SIT therapy on serum lipid levels in the HFD-fed male rats**

| **Group** | **TG**  **(mmol•L^-1^)** | **TC**  **(mmol•L^-1^)** | **HDL-C**  **(mmol•L^-1^)** | **LDL-C**  **(mmol•L^-1^)** |
| --- | --- | --- | --- | --- |
| SCD | 0.39±0.14 | 1±0.36 | 1.09±0.21 | 0.33±0.07 |
| HFD | 0.77±0.12^*^ | 1.44±0.28^*^ | 0.54±0.08^**^ | 0.68±0.14^*^ |
| HFD+SIT | 0.48±0.28^##^ | 1.04±0.27^#^ | 1.11±0.25^##^ | 0.42±0.09**^#^** |

*Data were expressed as mean±SEM (n=6). ^*^ P<0.05，^**^ P <0.01 vs. SCD group; ^#^ P<0.05，****^##^****P <0.01 vs. HFD group.*
