# Supplementary figures and images for: Sitagliptin Reduces Endothelial Dysfunction and Apoptosis Induced by High-Fat Diet and Palmitate in Thoracic Aortas and Endothelial Cells via ROS-ER Stress-CHOP Pathway
Source: Front Pharmacol. 2021 Aug 31;12:670389. doi: 10.3389/fphar.2021.670389 (PMC8438525; doi:10.3389/fphar.2021.670389)

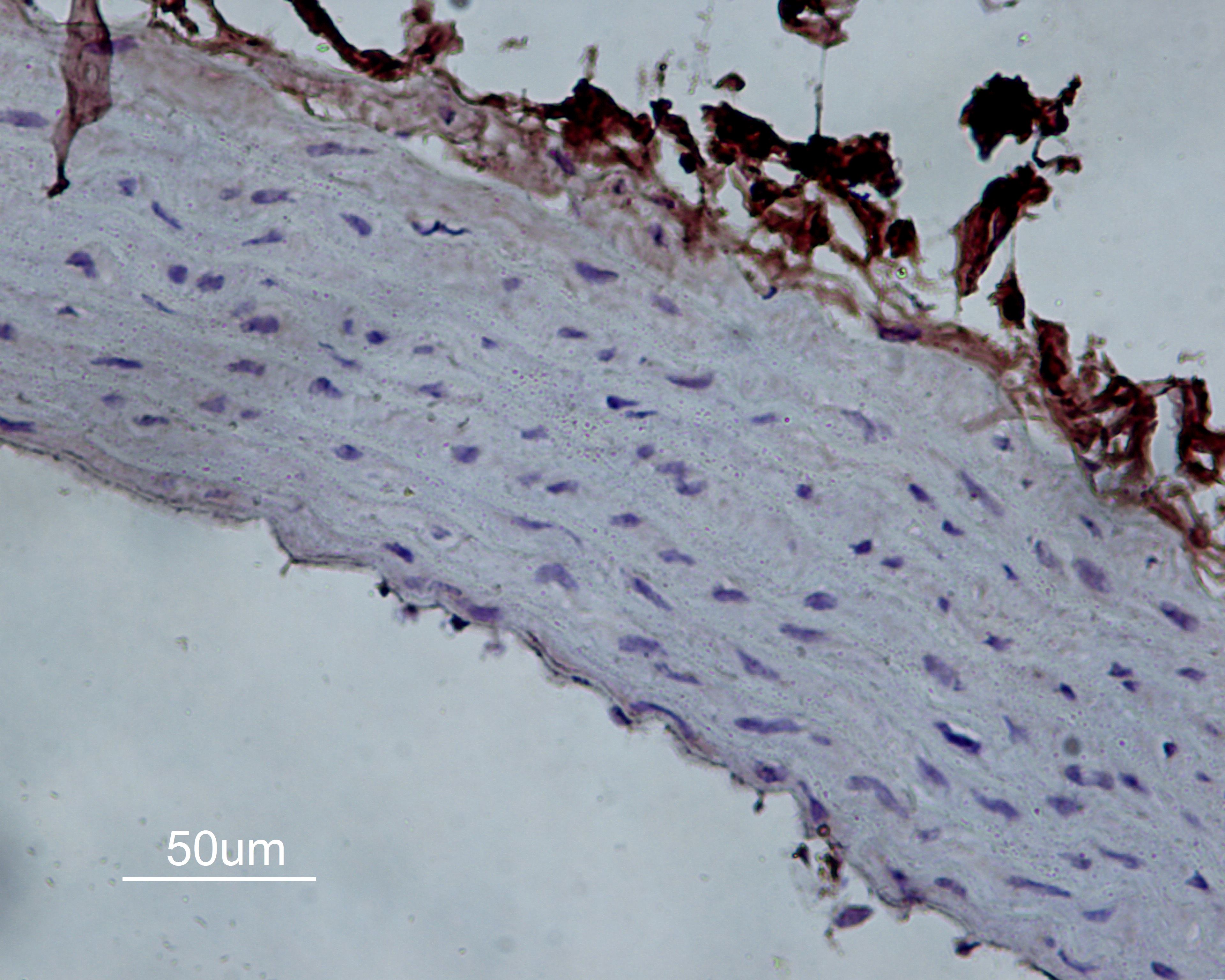

Supplement: Supplementary file 2 [file DataSheet1.zip › Animal experiment-RAW DATA/Fig 3 Original Figures/CD68 staining/HFD+SIT.jpg]

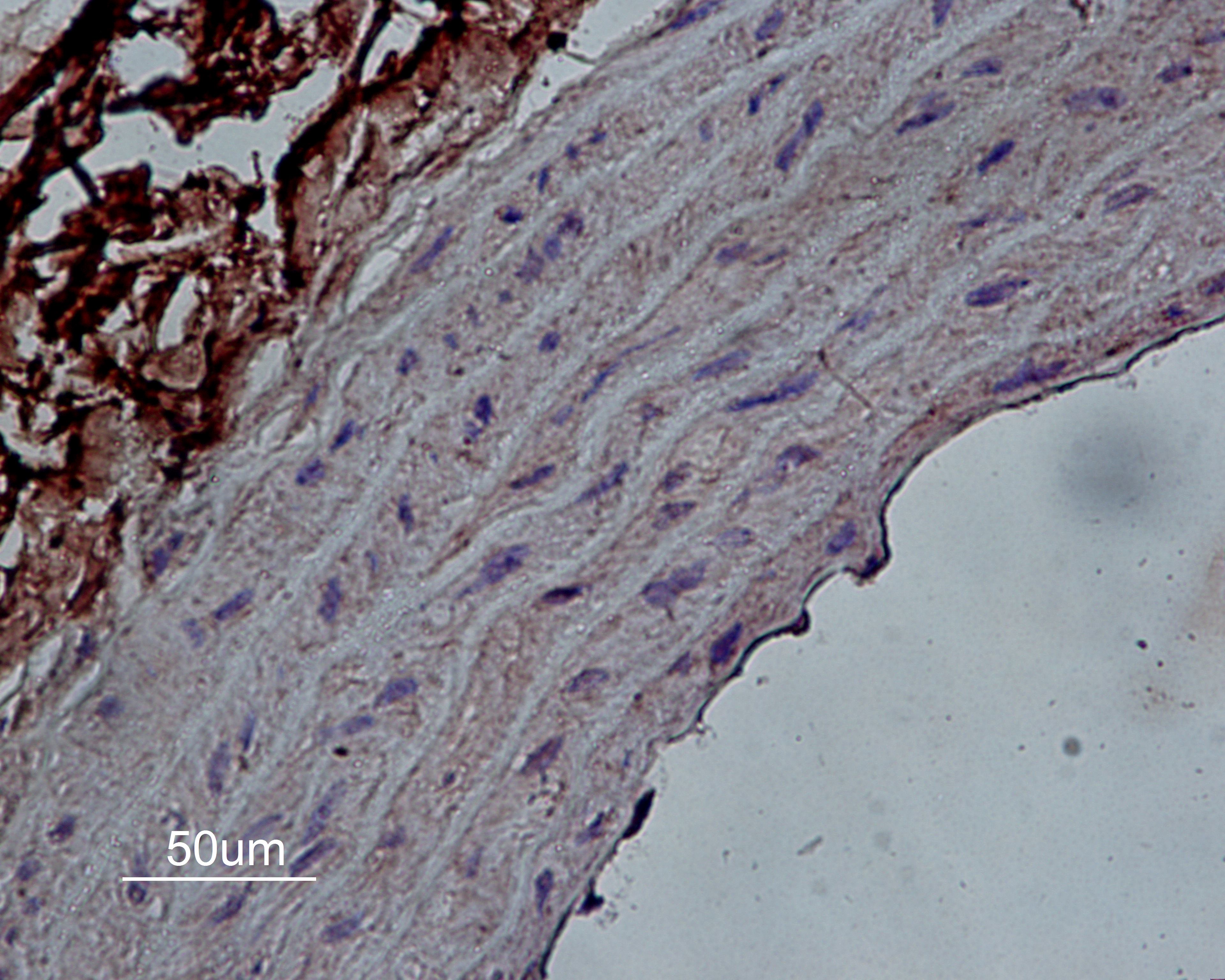

Supplement: Supplementary file 2 [file DataSheet1.zip › Animal experiment-RAW DATA/Fig 3 Original Figures/CD68 staining/HFD.jpg]

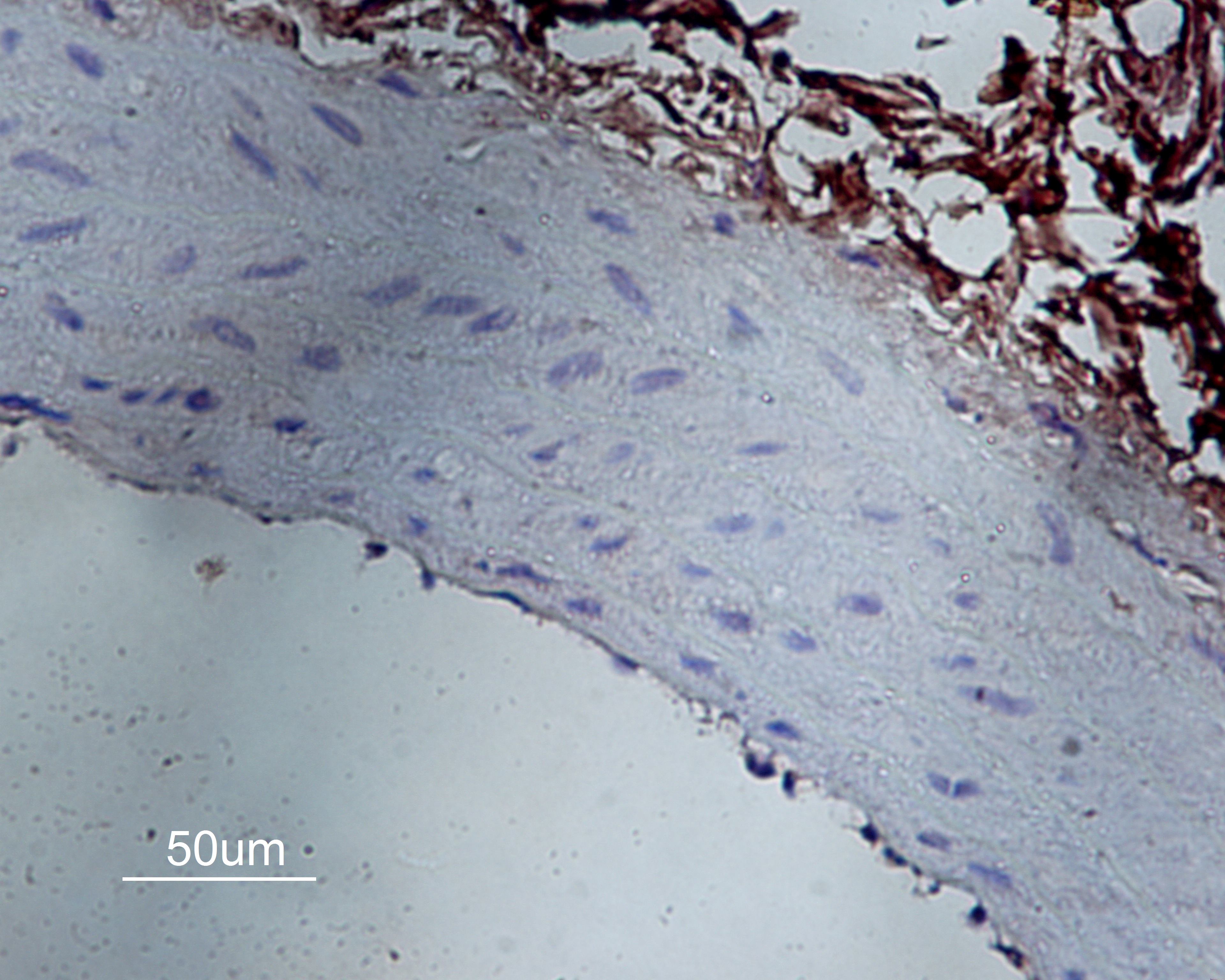

Supplement: Supplementary file 2 [file DataSheet1.zip › Animal experiment-RAW DATA/Fig 3 Original Figures/CD68 staining/SCD.jpg]

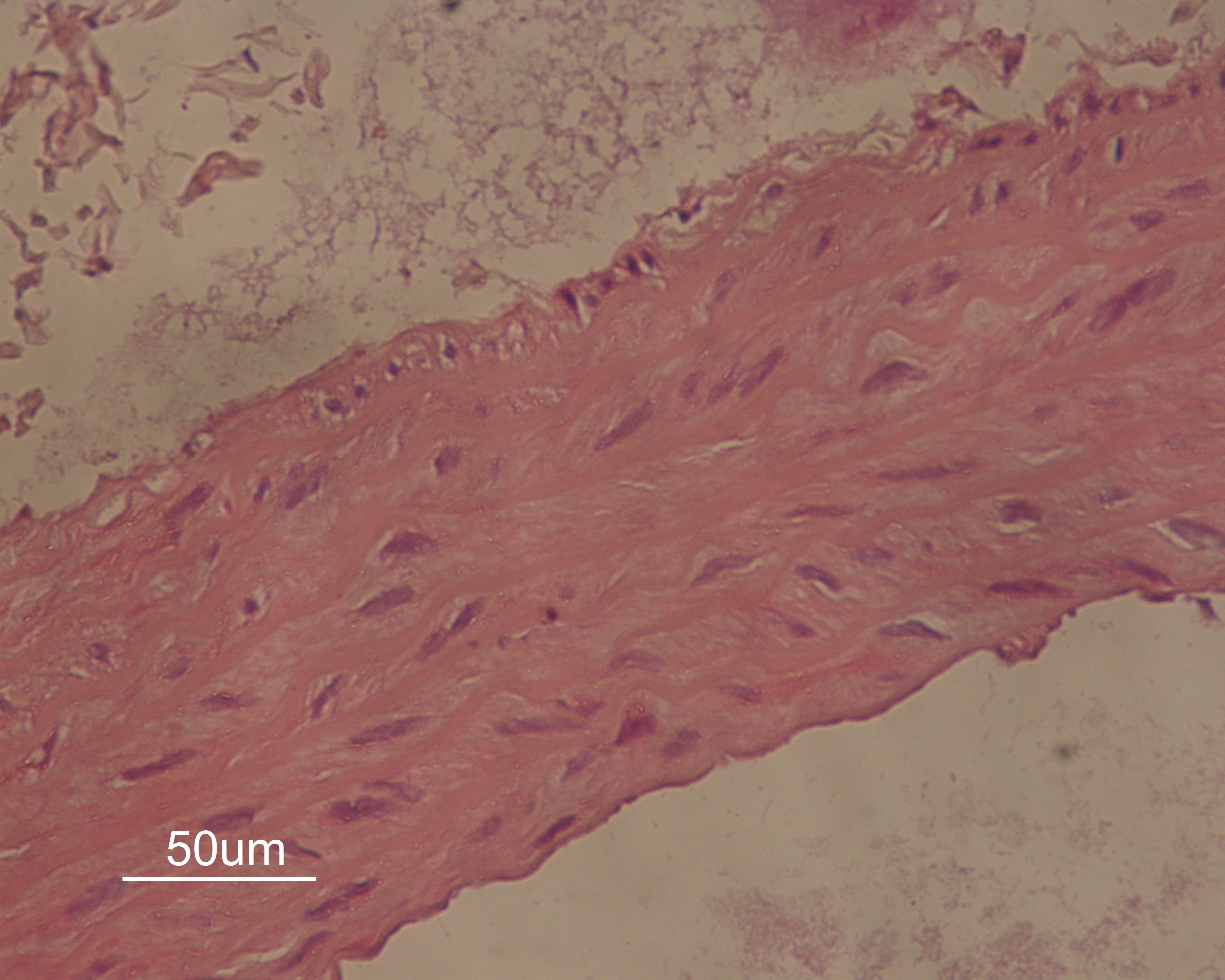

Supplement: Supplementary file 2 [file DataSheet1.zip › Animal experiment-RAW DATA/Fig 3 Original Figures/HE staining/HFD+SIT.jpg]

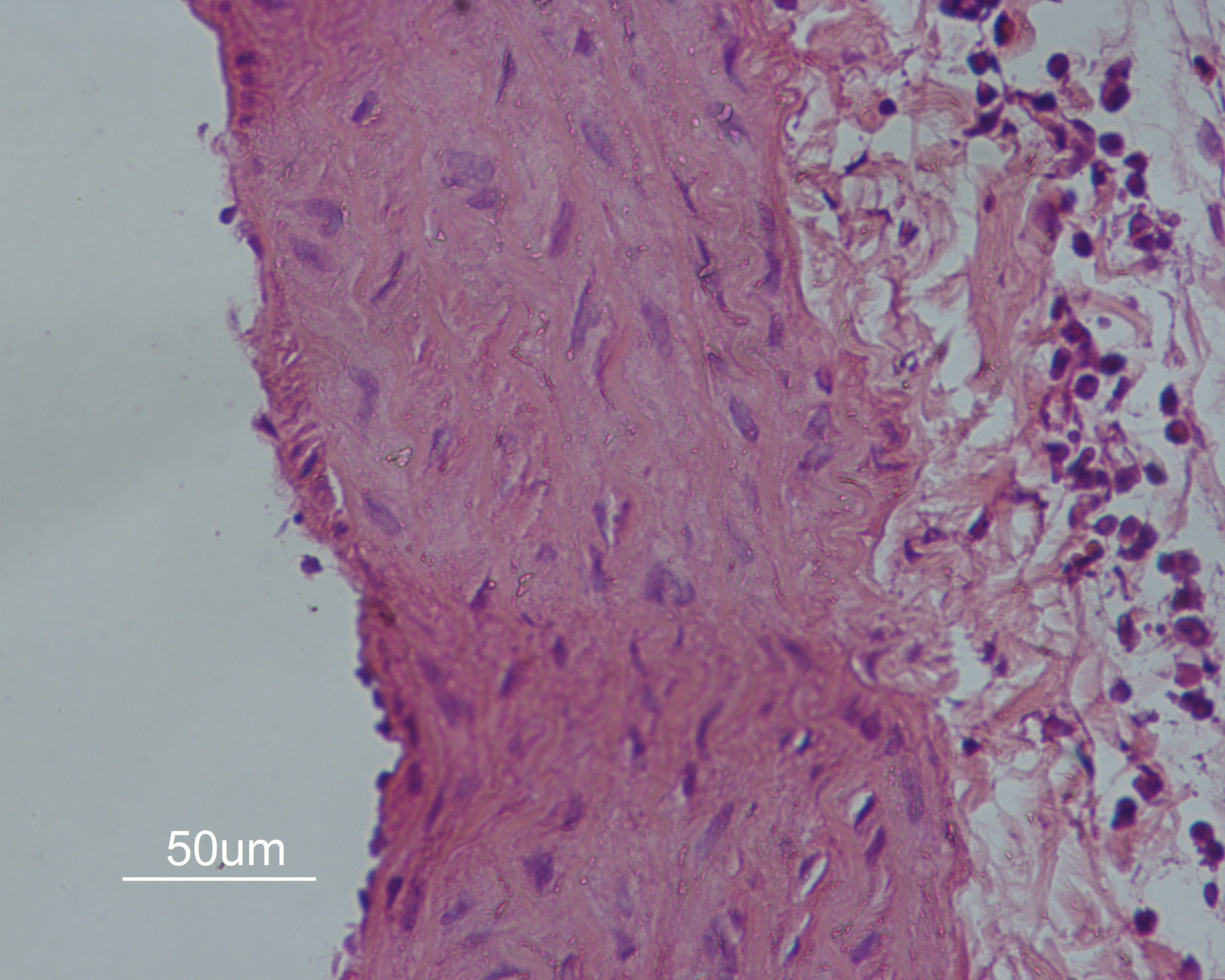

Supplement: Supplementary file 2 [file DataSheet1.zip › Animal experiment-RAW DATA/Fig 3 Original Figures/HE staining/HFD.jpg]

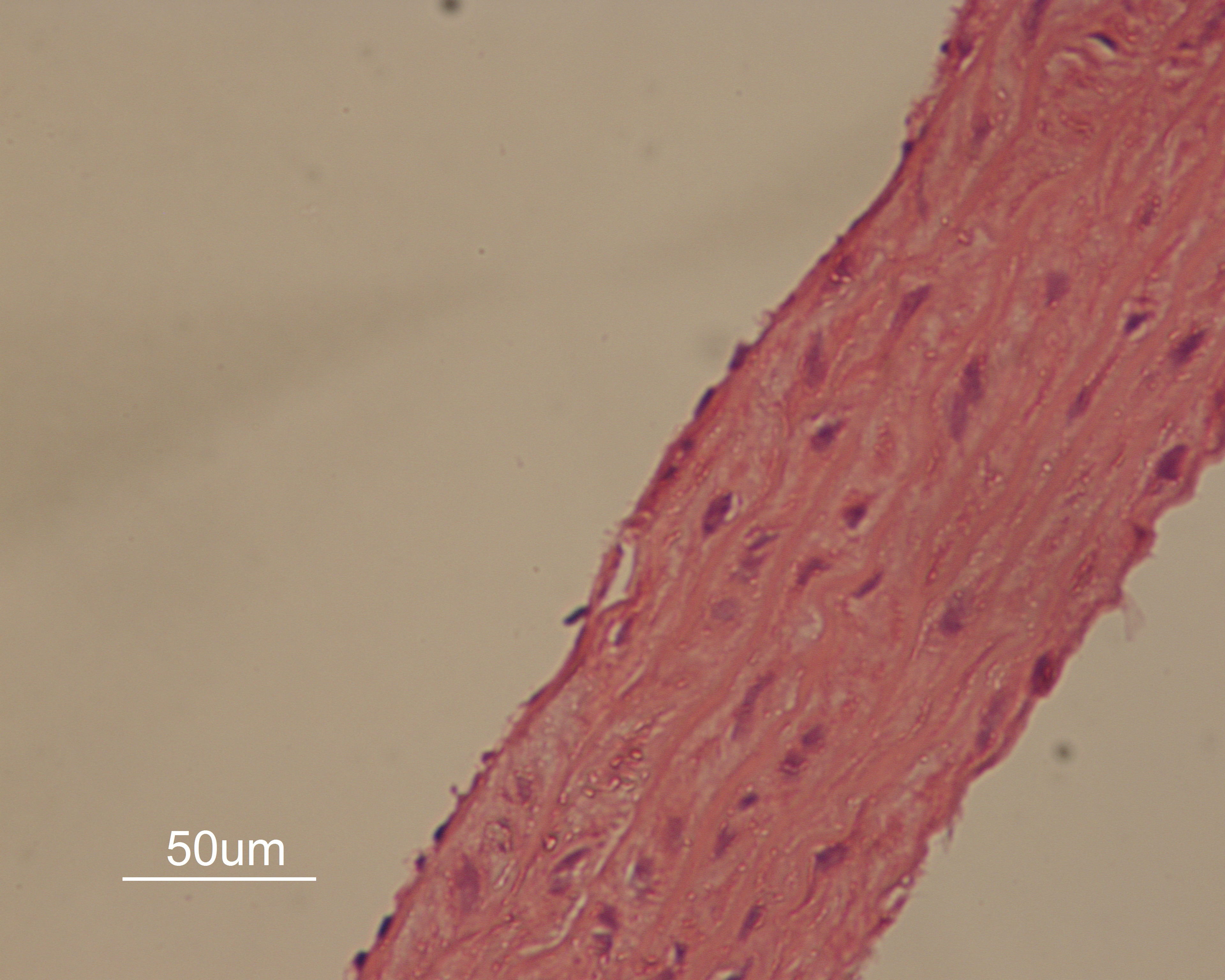

Supplement: Supplementary file 2 [file DataSheet1.zip › Animal experiment-RAW DATA/Fig 3 Original Figures/HE staining/SCD.jpg]

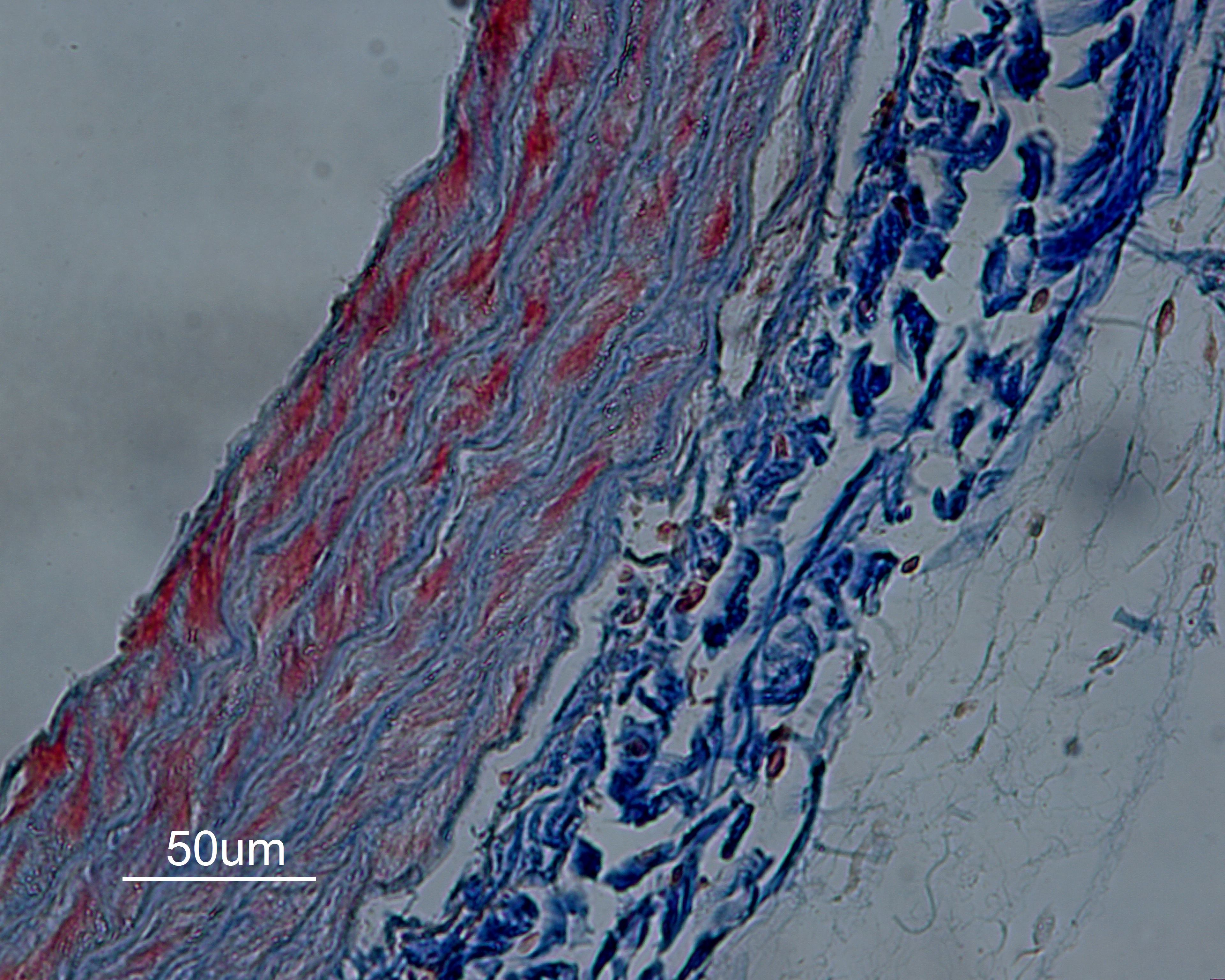

Supplement: Supplementary file 2 [file DataSheet1.zip › Animal experiment-RAW DATA/Fig 3 Original Figures/Masson staining/HFD+SIT.jpg]

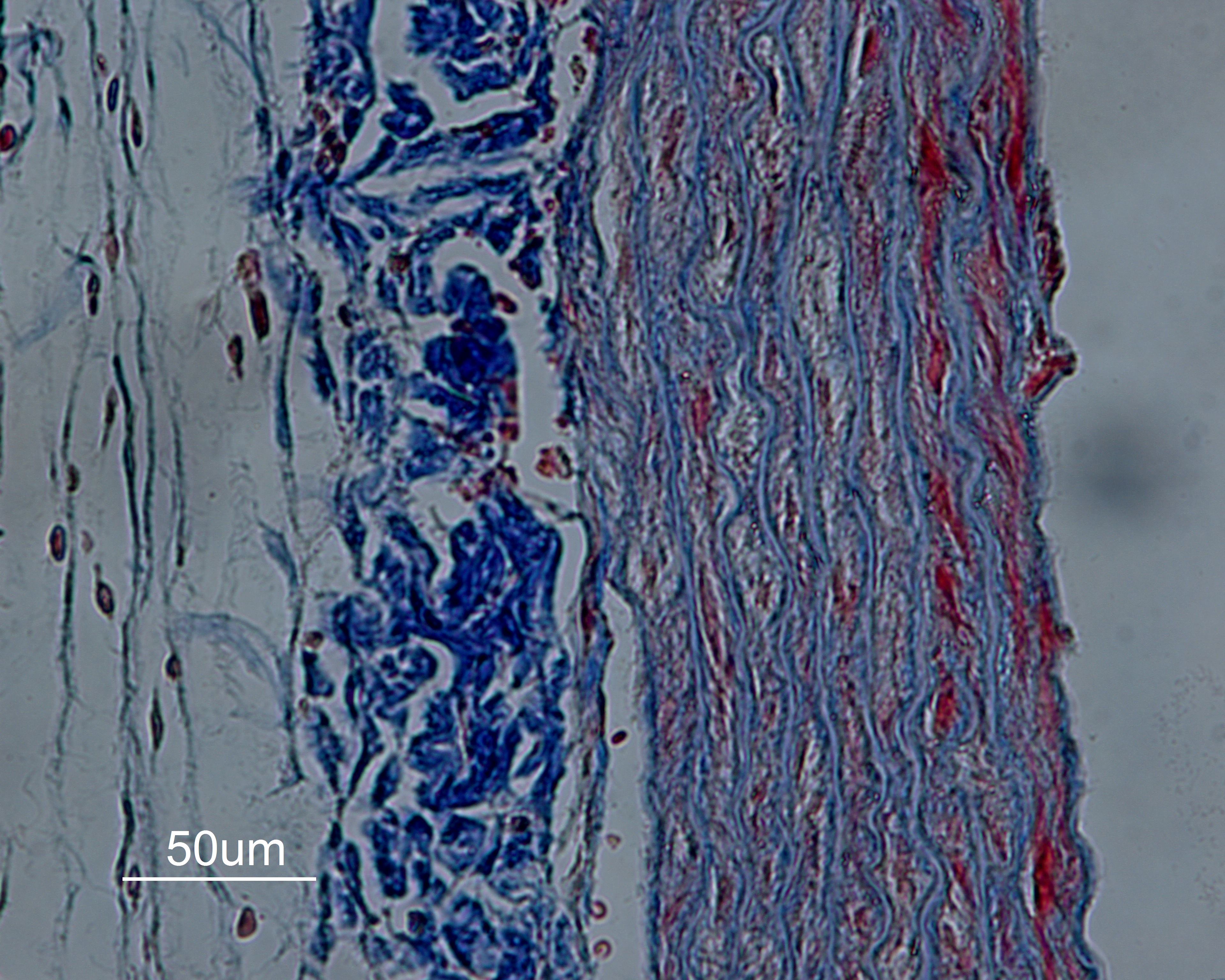

Supplement: Supplementary file 2 [file DataSheet1.zip › Animal experiment-RAW DATA/Fig 3 Original Figures/Masson staining/HFD.jpg]

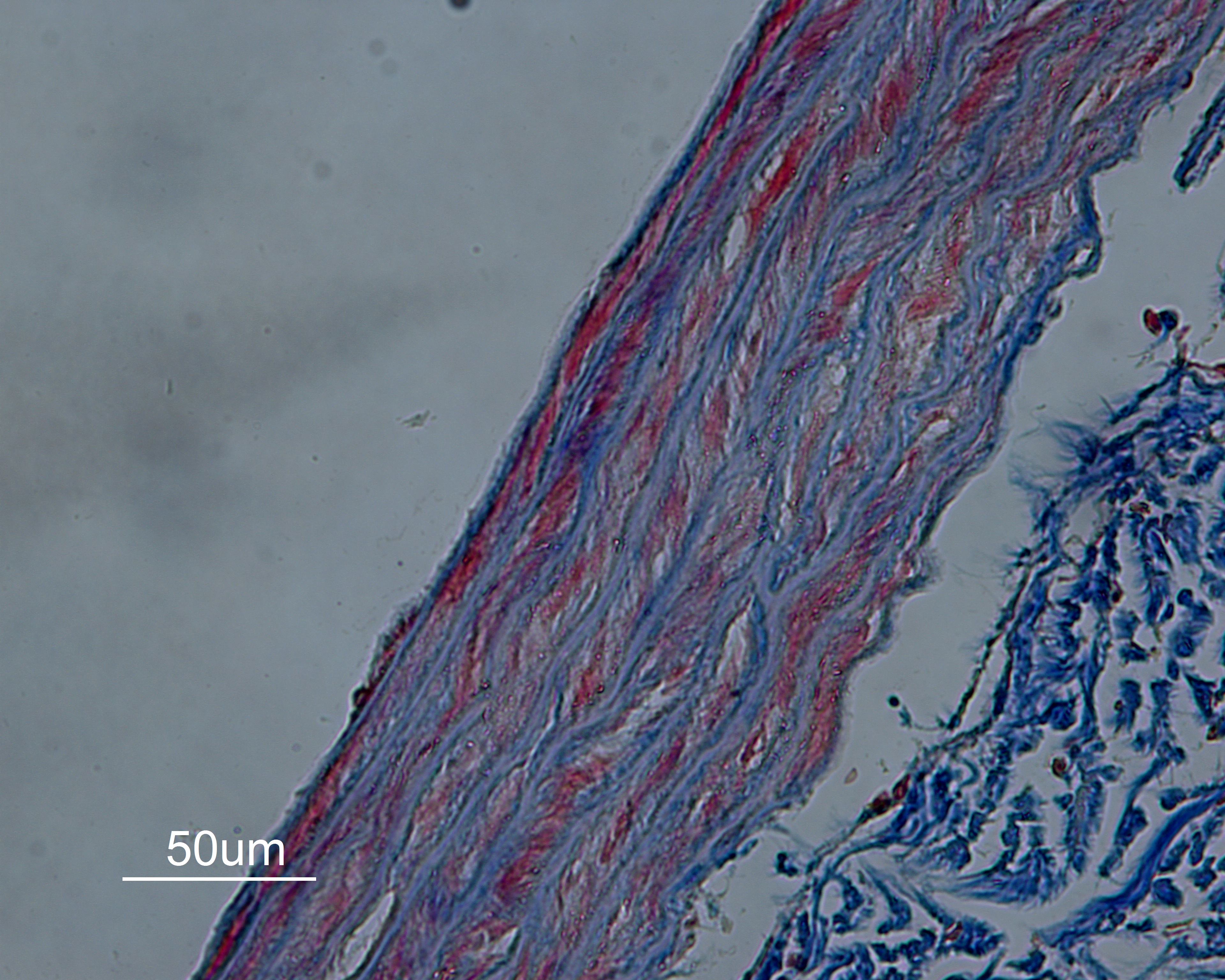

Supplement: Supplementary file 2 [file DataSheet1.zip › Animal experiment-RAW DATA/Fig 3 Original Figures/Masson staining/SCD.jpg]

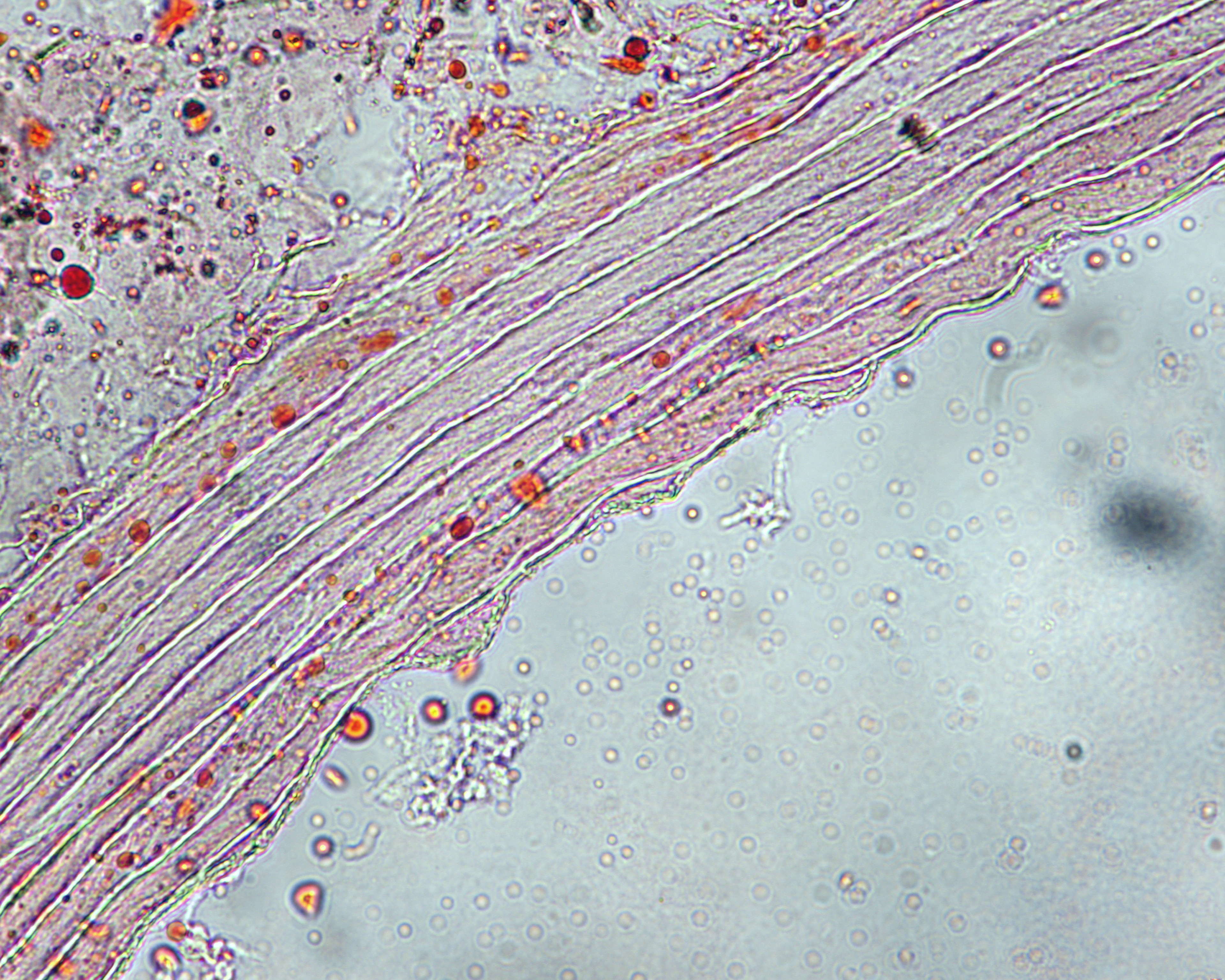

Supplement: Supplementary file 2 [file DataSheet1.zip › Animal experiment-RAW DATA/Fig 3 Original Figures/Oil Red O staining/HFD+SIT.jpg]

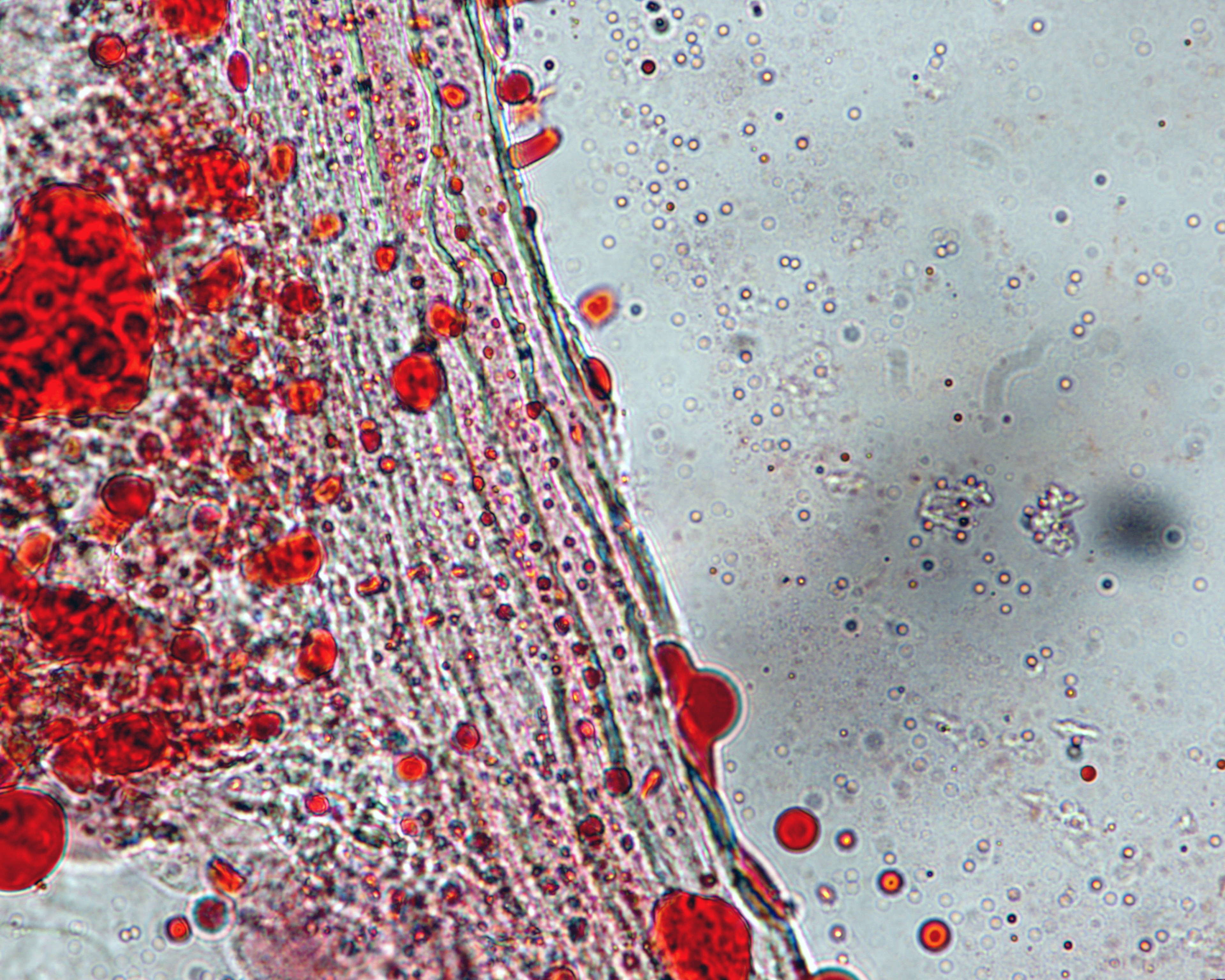

Supplement: Supplementary file 2 [file DataSheet1.zip › Animal experiment-RAW DATA/Fig 3 Original Figures/Oil Red O staining/HFD.jpg]

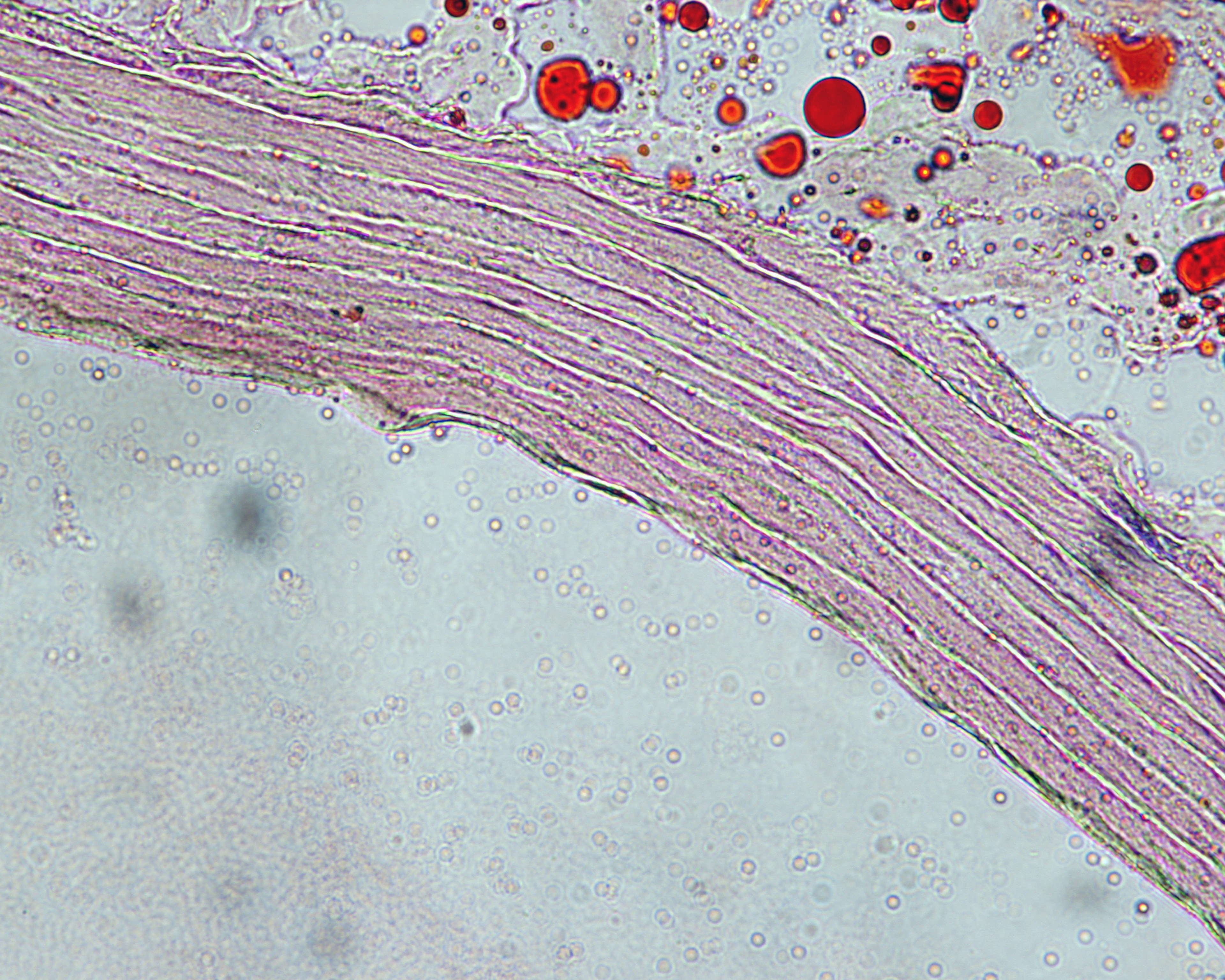

Supplement: Supplementary file 2 [file DataSheet1.zip › Animal experiment-RAW DATA/Fig 3 Original Figures/Oil Red O staining/SCD.jpg]

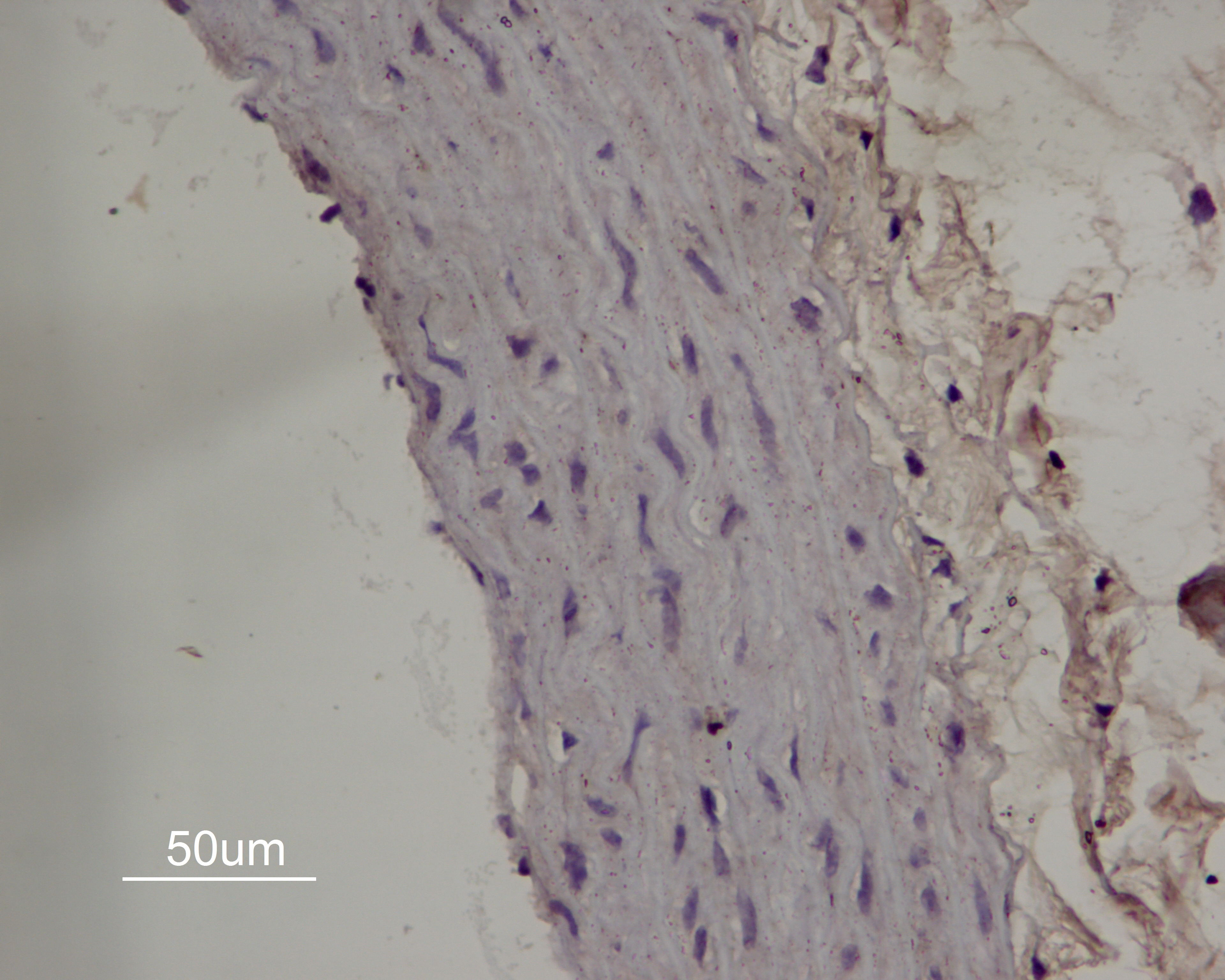

Supplement: Supplementary file 2 [file DataSheet1.zip › Animal experiment-RAW DATA/Fig 3 Original Figures/TUNEL staining/HFD+SIT.jpg]

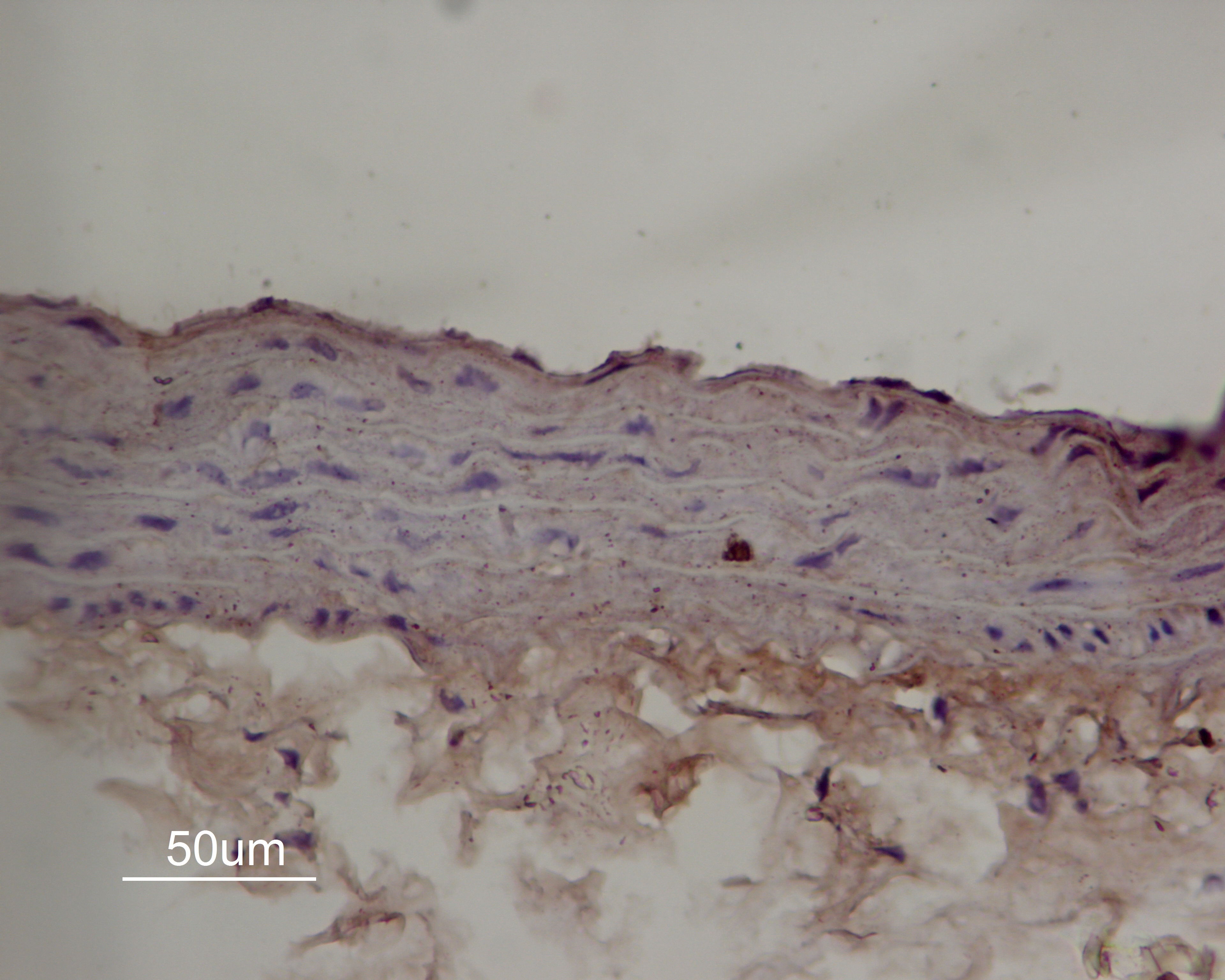

Supplement: Supplementary file 2 [file DataSheet1.zip › Animal experiment-RAW DATA/Fig 3 Original Figures/TUNEL staining/HFD.jpg]

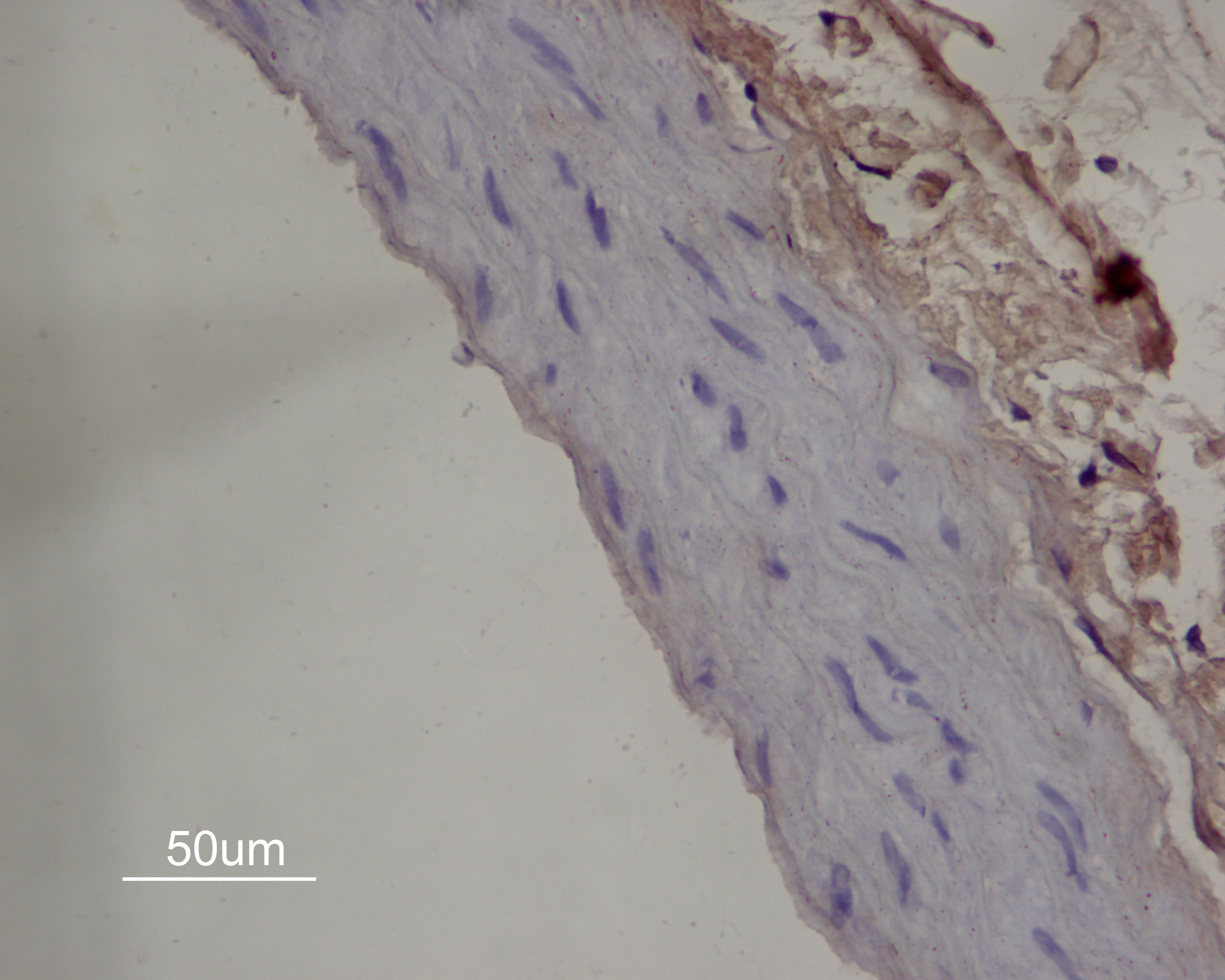

Supplement: Supplementary file 2 [file DataSheet1.zip › Animal experiment-RAW DATA/Fig 3 Original Figures/TUNEL staining/SCD.jpg]

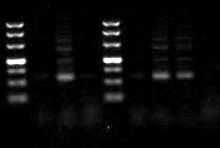

Supplement: Supplementary file 2 [file DataSheet1.zip › Animal experiment-RAW DATA/Fig 4 Original Figures/PCR/ACC+XBP1.jpg]

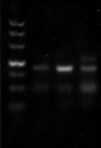

Supplement: Supplementary file 2 [file DataSheet1.zip › Animal experiment-RAW DATA/Fig 4 Original Figures/PCR/CHOP-the top strips in Aorta.tif]

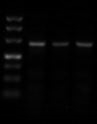

Supplement: Supplementary file 2 [file DataSheet1.zip › Animal experiment-RAW DATA/Fig 4 Original Figures/PCR/CPT1b in Aorta.tif]

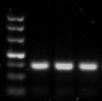

Supplement: Supplementary file 2 [file DataSheet1.zip › Animal experiment-RAW DATA/Fig 4 Original Figures/PCR/GAPDH in Aorta.tif]

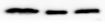

Supplement: Supplementary file 2 [file DataSheet1.zip › Animal experiment-RAW DATA/Fig 4 Original Figures/Western Blot/Fig4C-BCL-2.JPG]

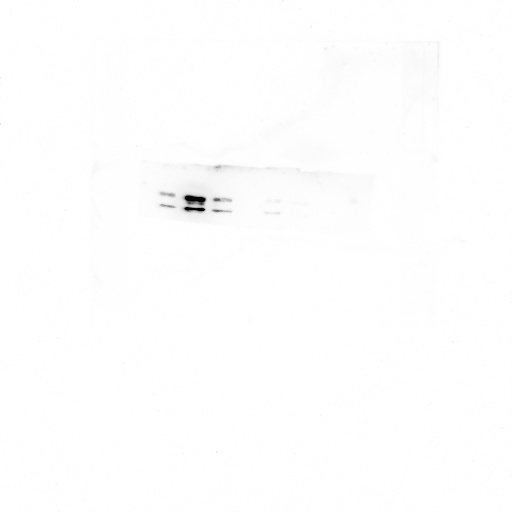

Supplement: Supplementary file 2 [file DataSheet1.zip › Animal experiment-RAW DATA/Fig 4 Original Figures/Western Blot/Fig4C-CHOP.jpg]

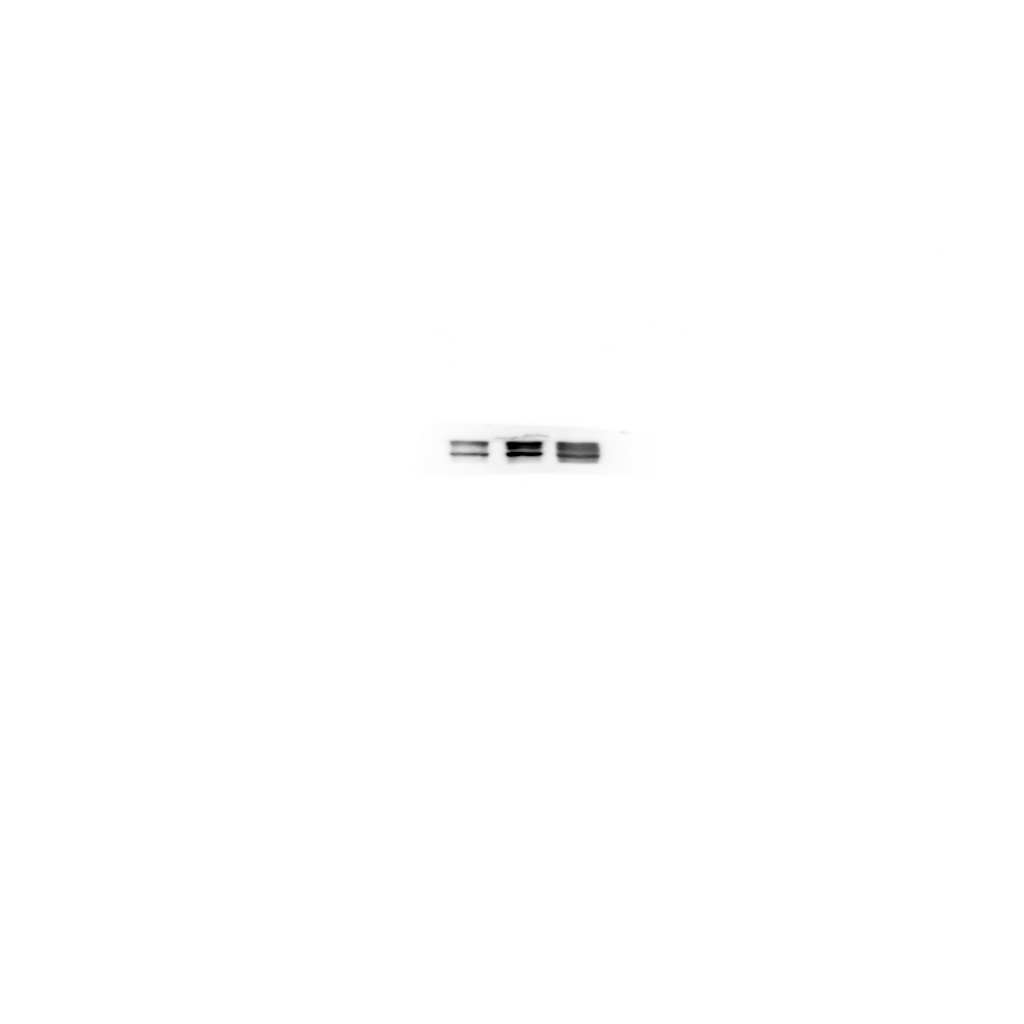

Supplement: Supplementary file 2 [file DataSheet1.zip › Animal experiment-RAW DATA/Fig 4 Original Figures/Western Blot/Fig4C-GRP78.jpg]

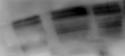

Supplement: Supplementary file 2 [file DataSheet1.zip › Animal experiment-RAW DATA/Fig 4 Original Figures/Western Blot/Fig4C-IREa┴.JPG]

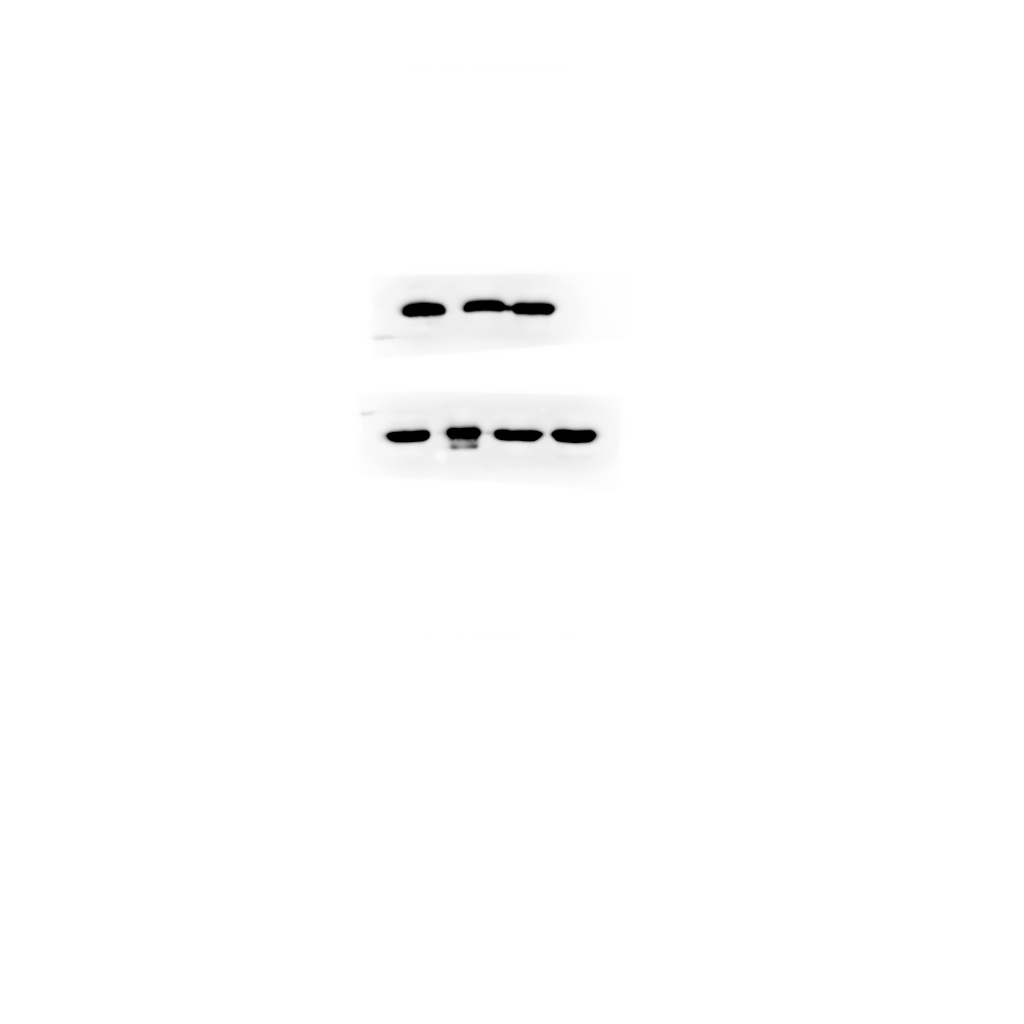

Supplement: Supplementary file 2 [file DataSheet1.zip › Animal experiment-RAW DATA/Fig 4 Original Figures/Western Blot/Fig4C-actin-upper.jpg]

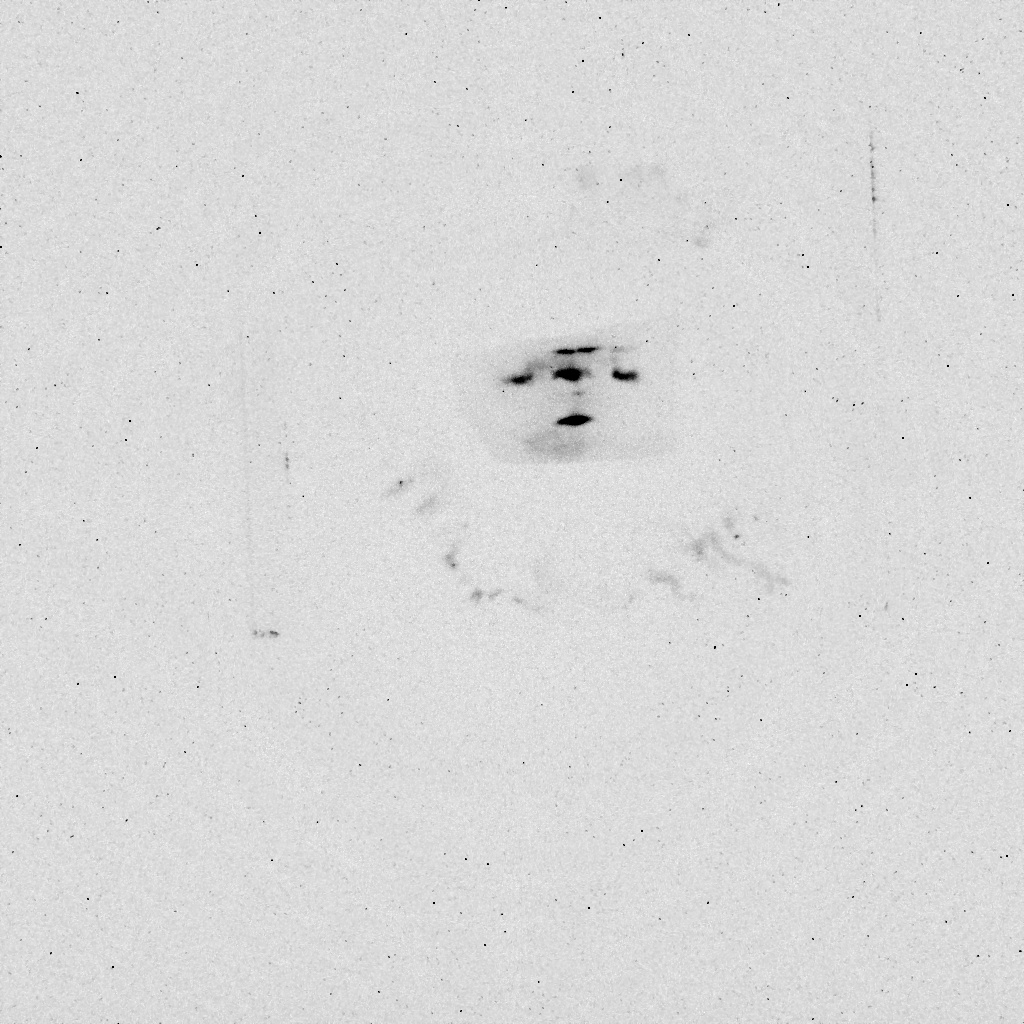

Supplement: Supplementary file 2 [file DataSheet1.zip › Animal experiment-RAW DATA/Fig 4 Original Figures/Western Blot/Fig4C-bax.jpg]

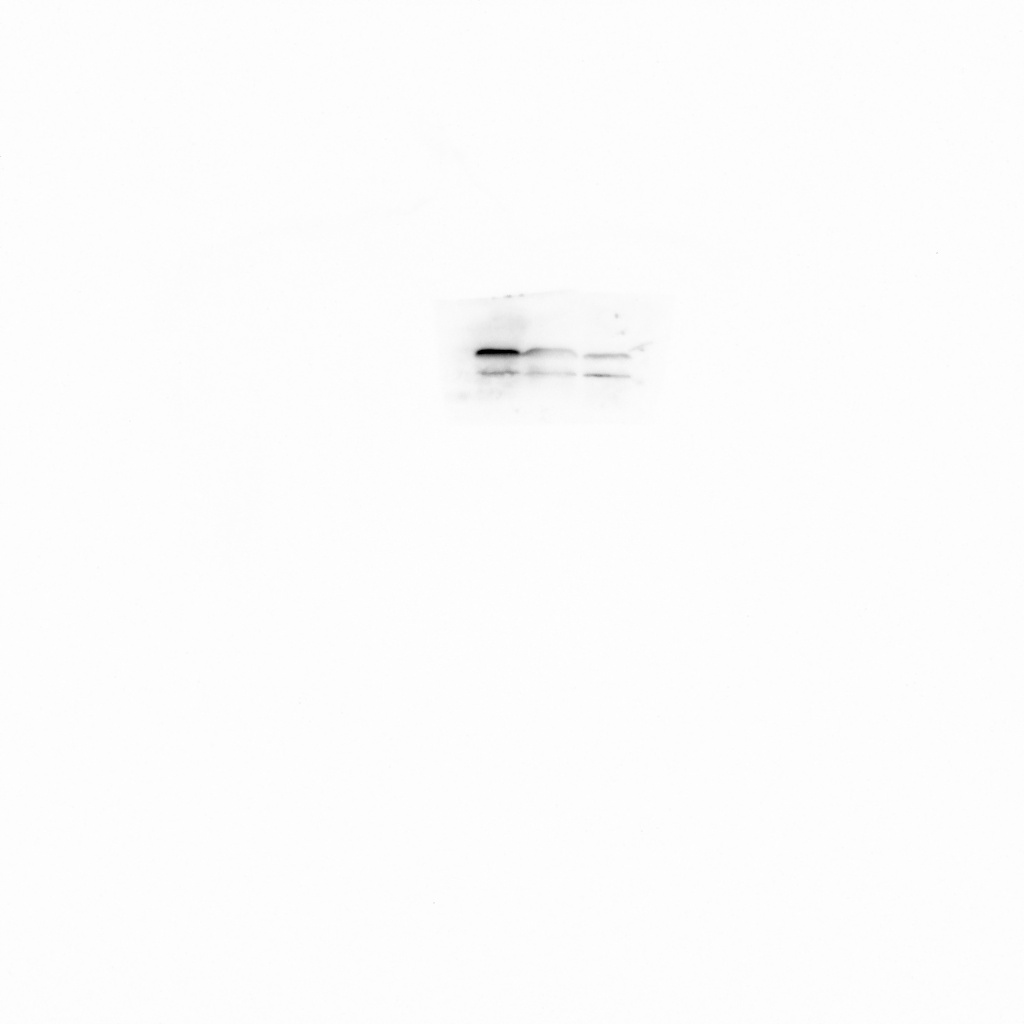

Supplement: Supplementary file 2 [file DataSheet1.zip › Animal experiment-RAW DATA/Fig 4 Original Figures/Western Blot/Fig4E-JNK.jpg]

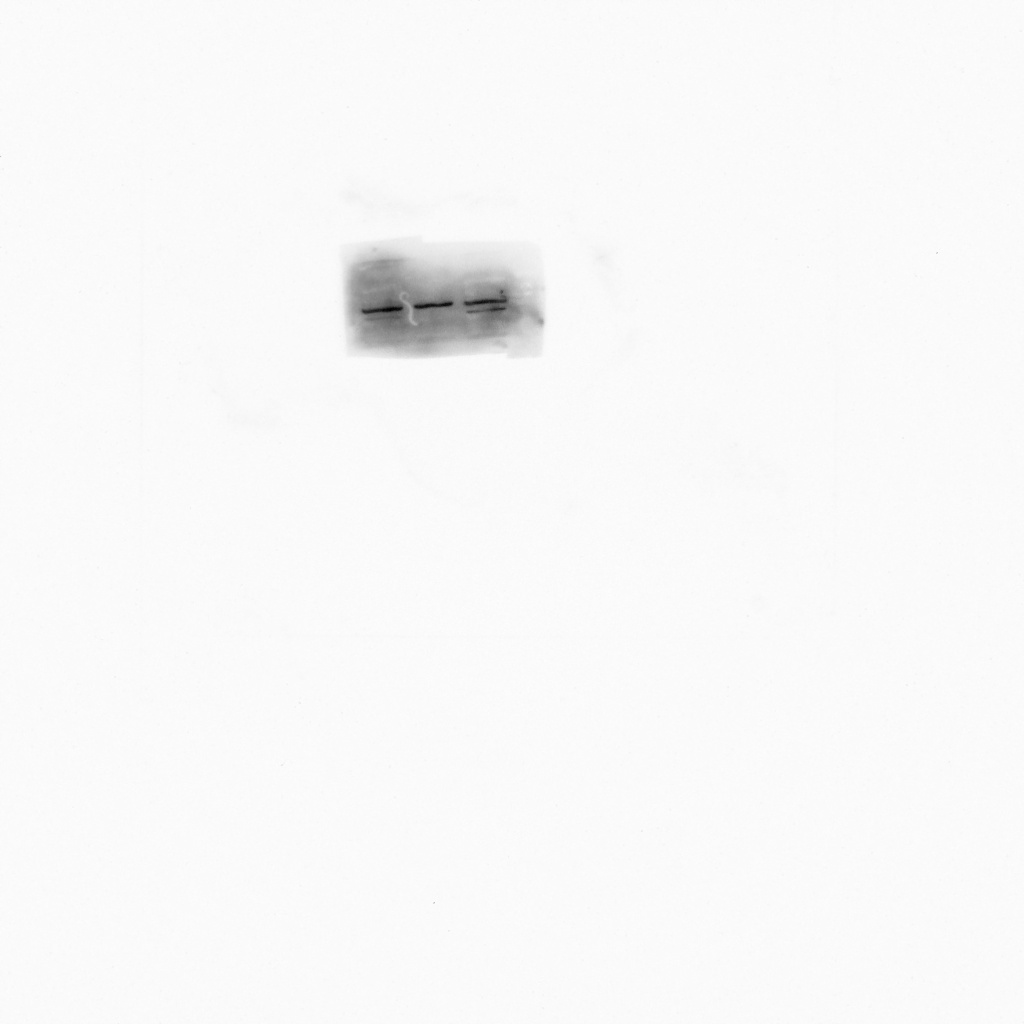

Supplement: Supplementary file 2 [file DataSheet1.zip › Animal experiment-RAW DATA/Fig 4 Original Figures/Western Blot/Fig4E-eNOS.jpg]

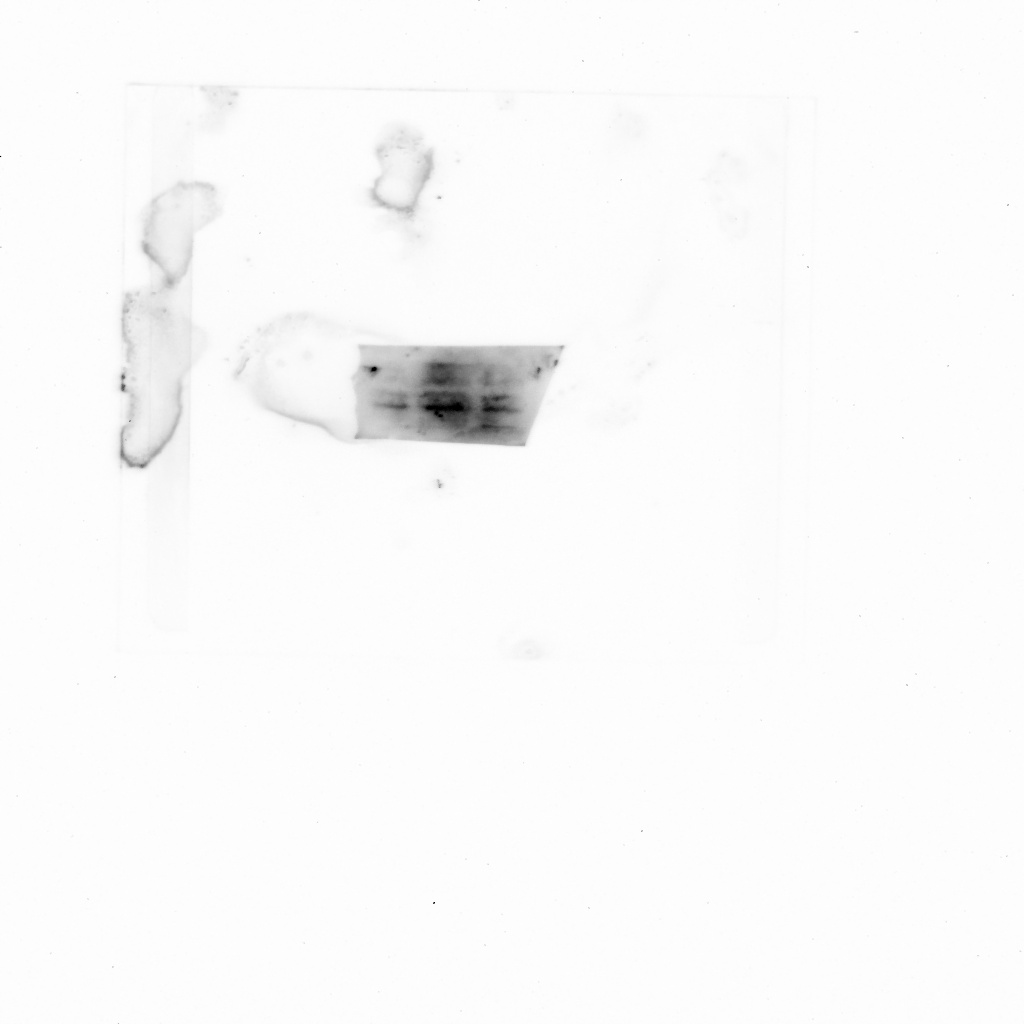

Supplement: Supplementary file 2 [file DataSheet1.zip › Animal experiment-RAW DATA/Fig 4 Original Figures/Western Blot/Fig4E-p-JNK.jpg]

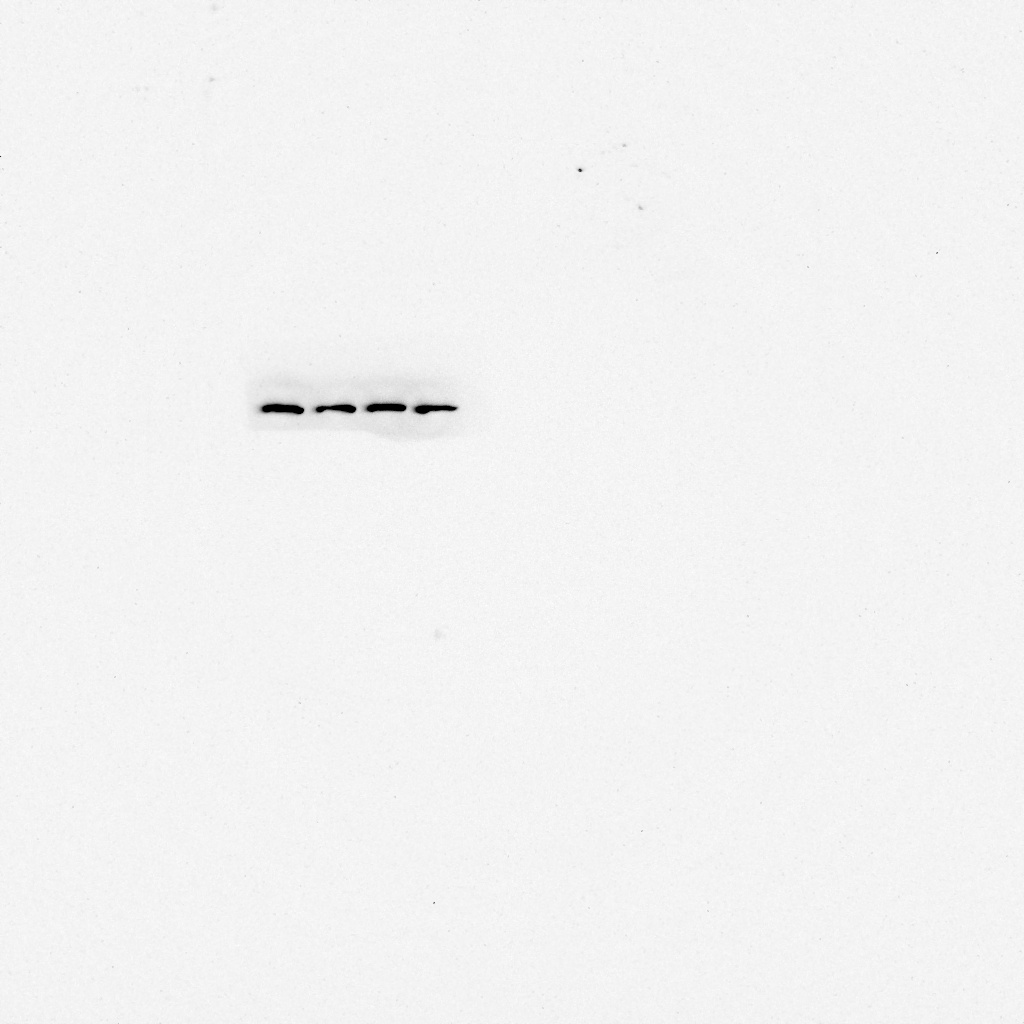

Supplement: Supplementary file 2 [file DataSheet1.zip › Animal experiment-RAW DATA/Fig 4 Original Figures/Western Blot/Fig4E-p-eNOS the left 3 bands.jpg]

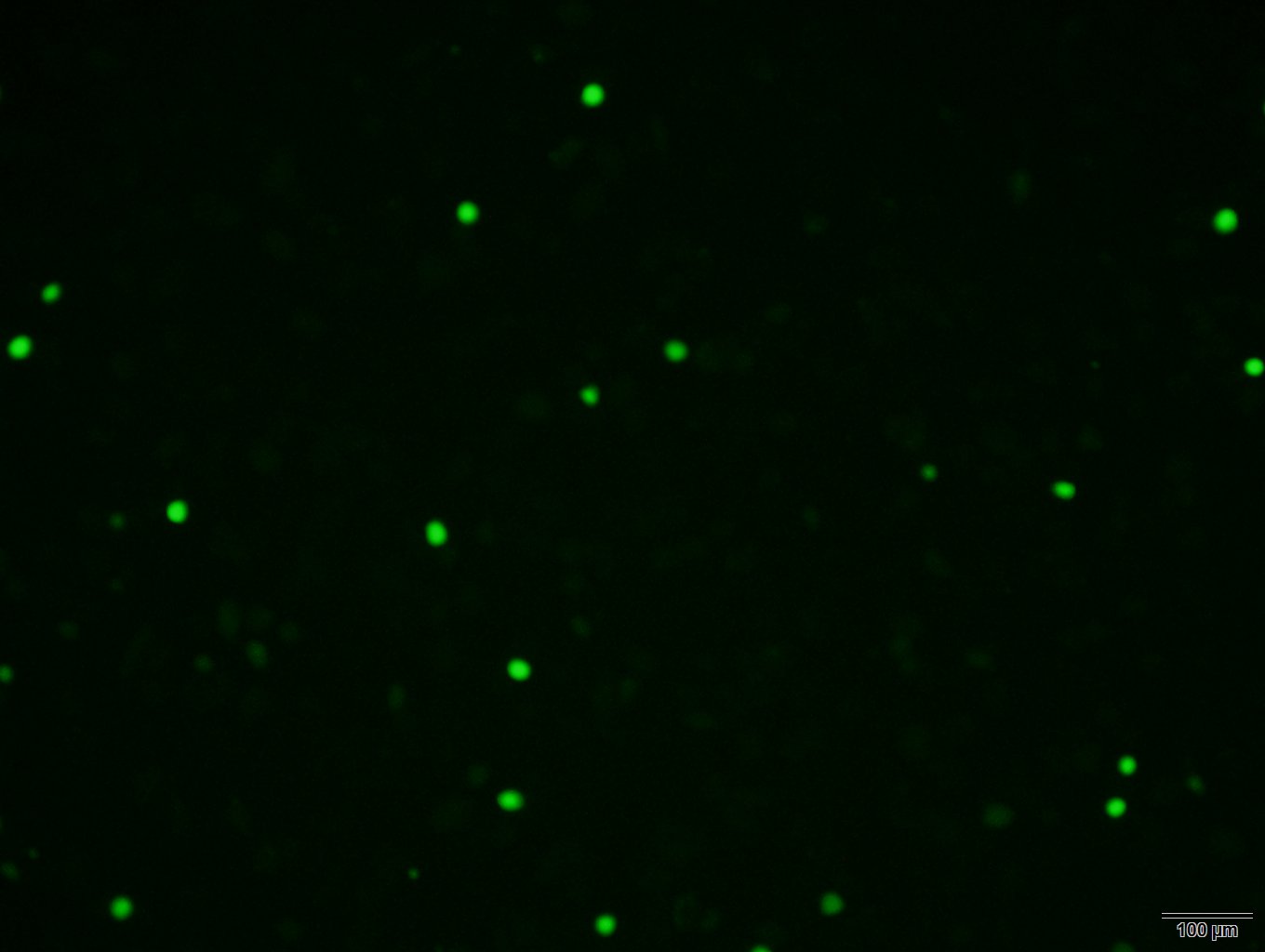

Supplement: Supplementary file 3 [file DataSheet2.ZIP › Cell experiment-RAW DATA/Fig 5/Fig5B-ROS-Original Figures/CON-.jpg]

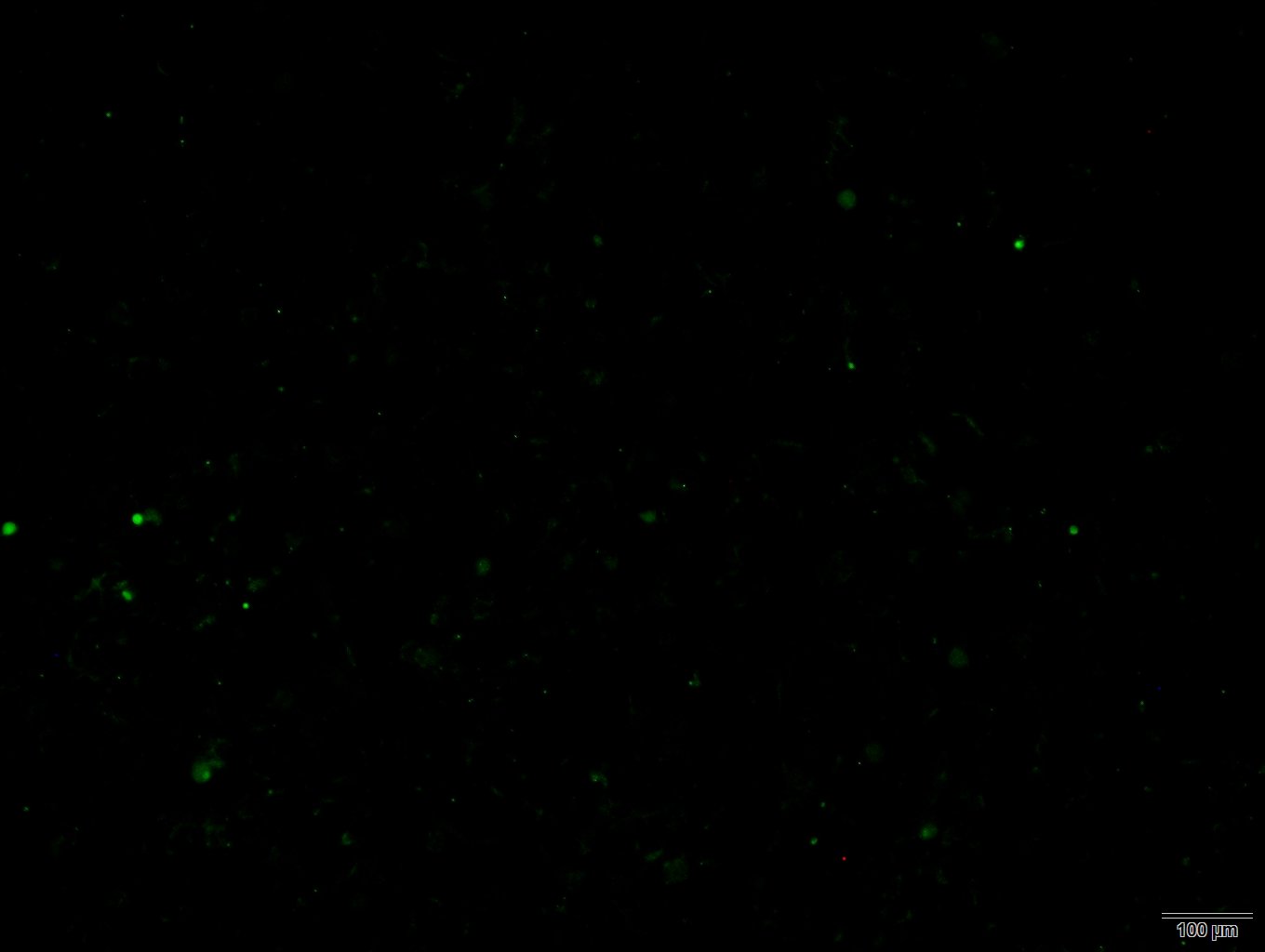

Supplement: Supplementary file 3 [file DataSheet2.ZIP › Cell experiment-RAW DATA/Fig 5/Fig5B-ROS-Original Figures/NC.jpg]

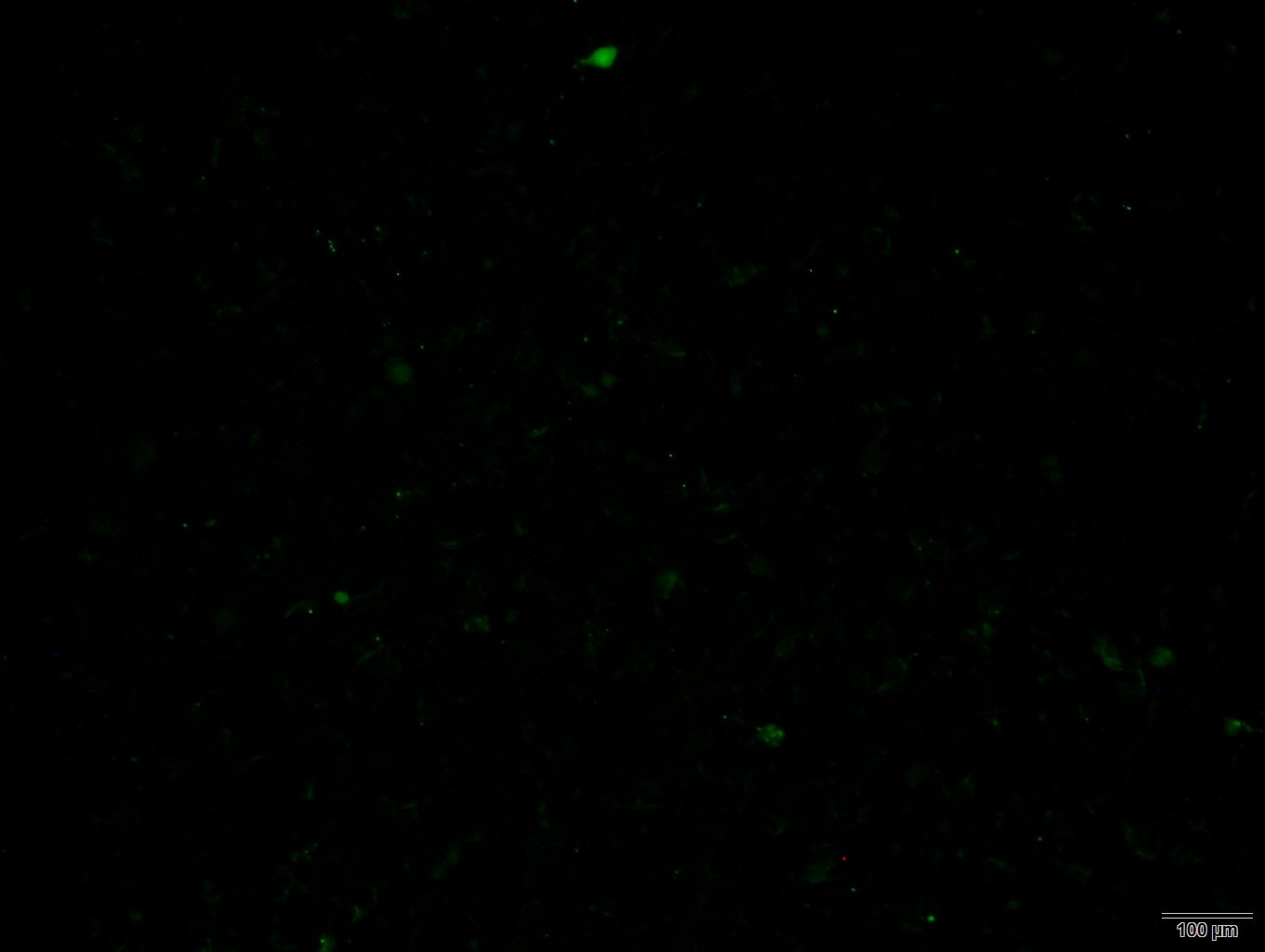

Supplement: Supplementary file 3 [file DataSheet2.ZIP › Cell experiment-RAW DATA/Fig 5/Fig5B-ROS-Original Figures/PA+PBA.jpg]

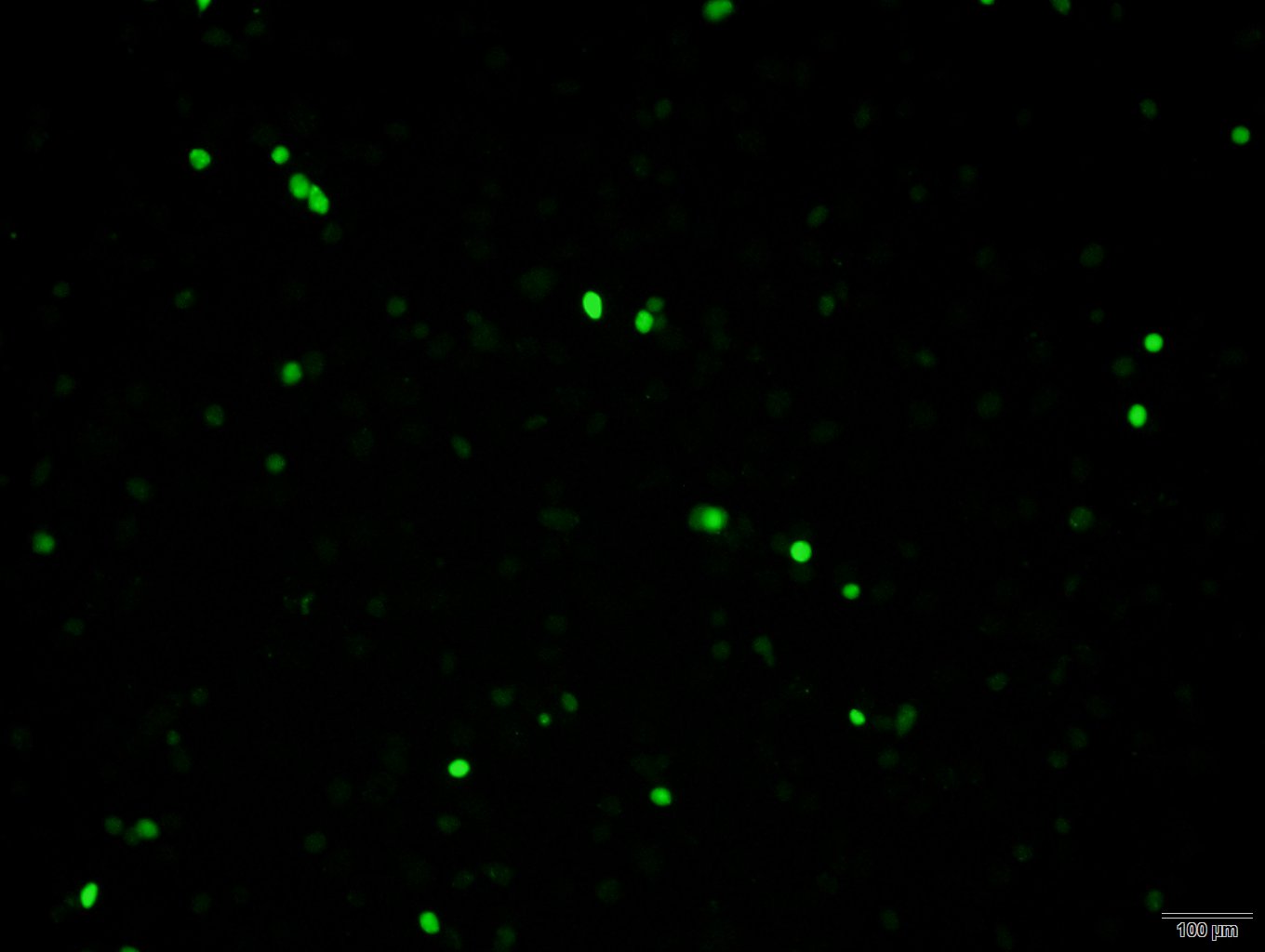

Supplement: Supplementary file 3 [file DataSheet2.ZIP › Cell experiment-RAW DATA/Fig 5/Fig5B-ROS-Original Figures/PA+SIT-H.jpg]

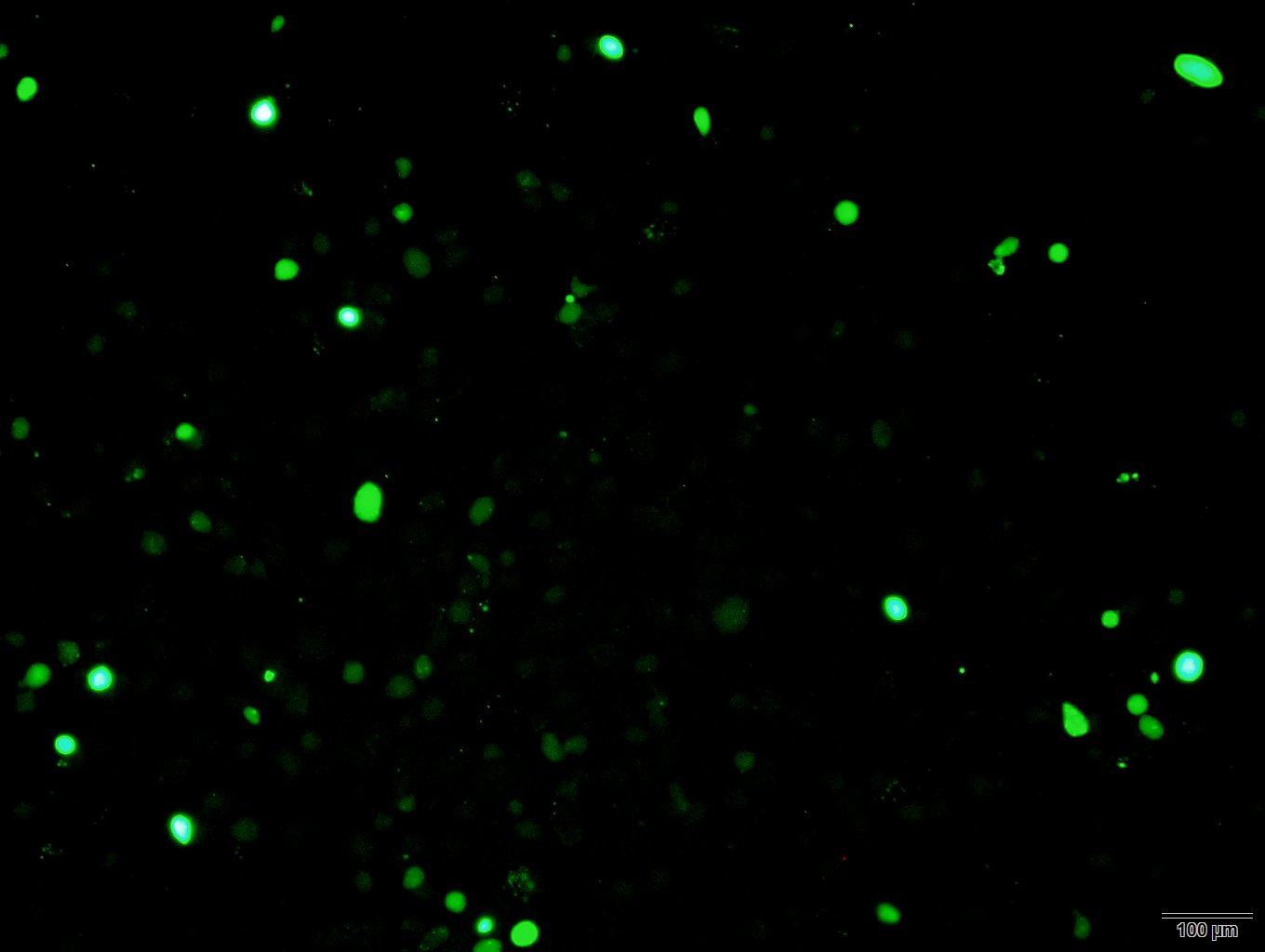

Supplement: Supplementary file 3 [file DataSheet2.ZIP › Cell experiment-RAW DATA/Fig 5/Fig5B-ROS-Original Figures/PA+SIT-L.jpg]

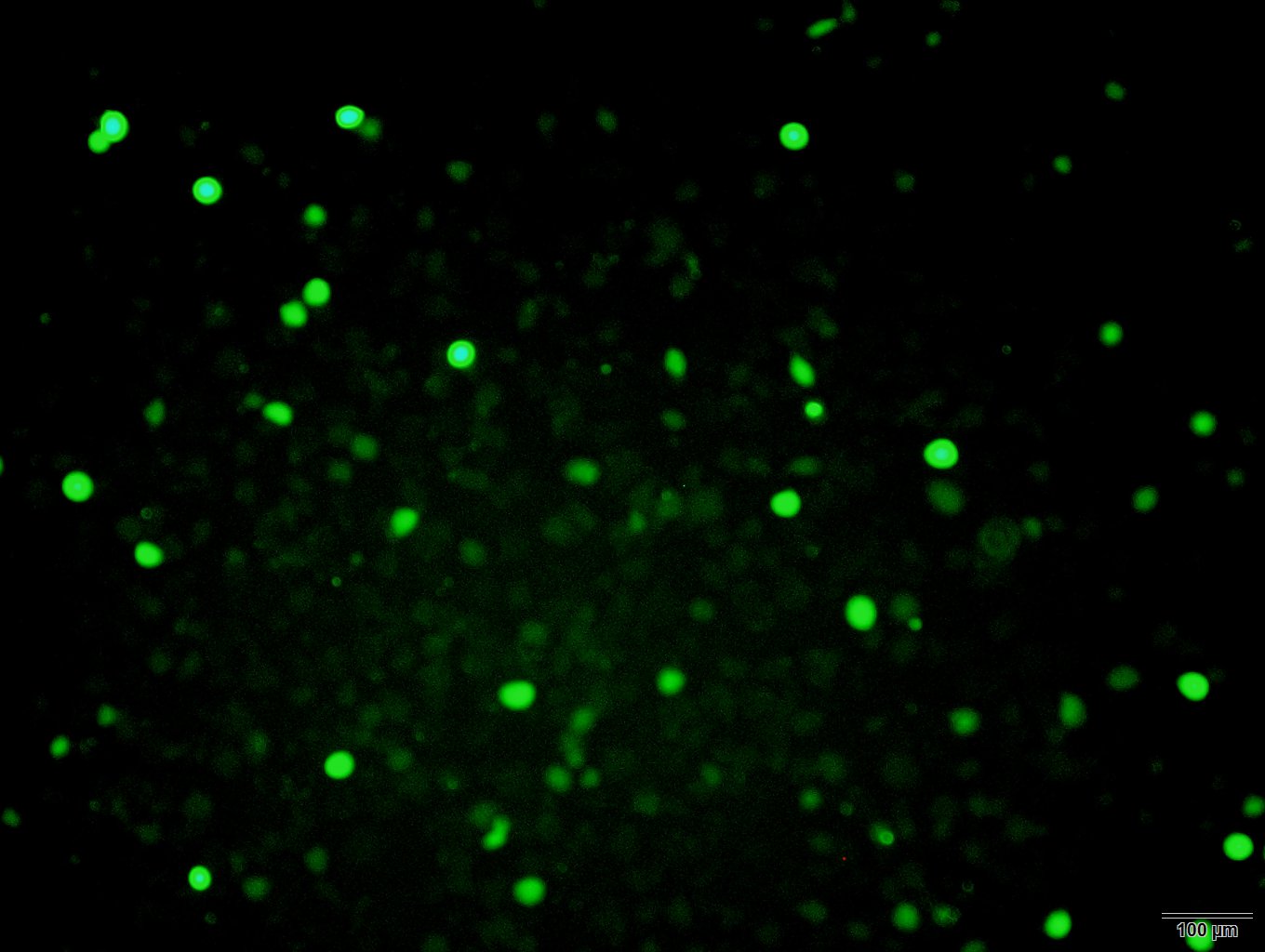

Supplement: Supplementary file 3 [file DataSheet2.ZIP › Cell experiment-RAW DATA/Fig 5/Fig5B-ROS-Original Figures/PA.jpg]

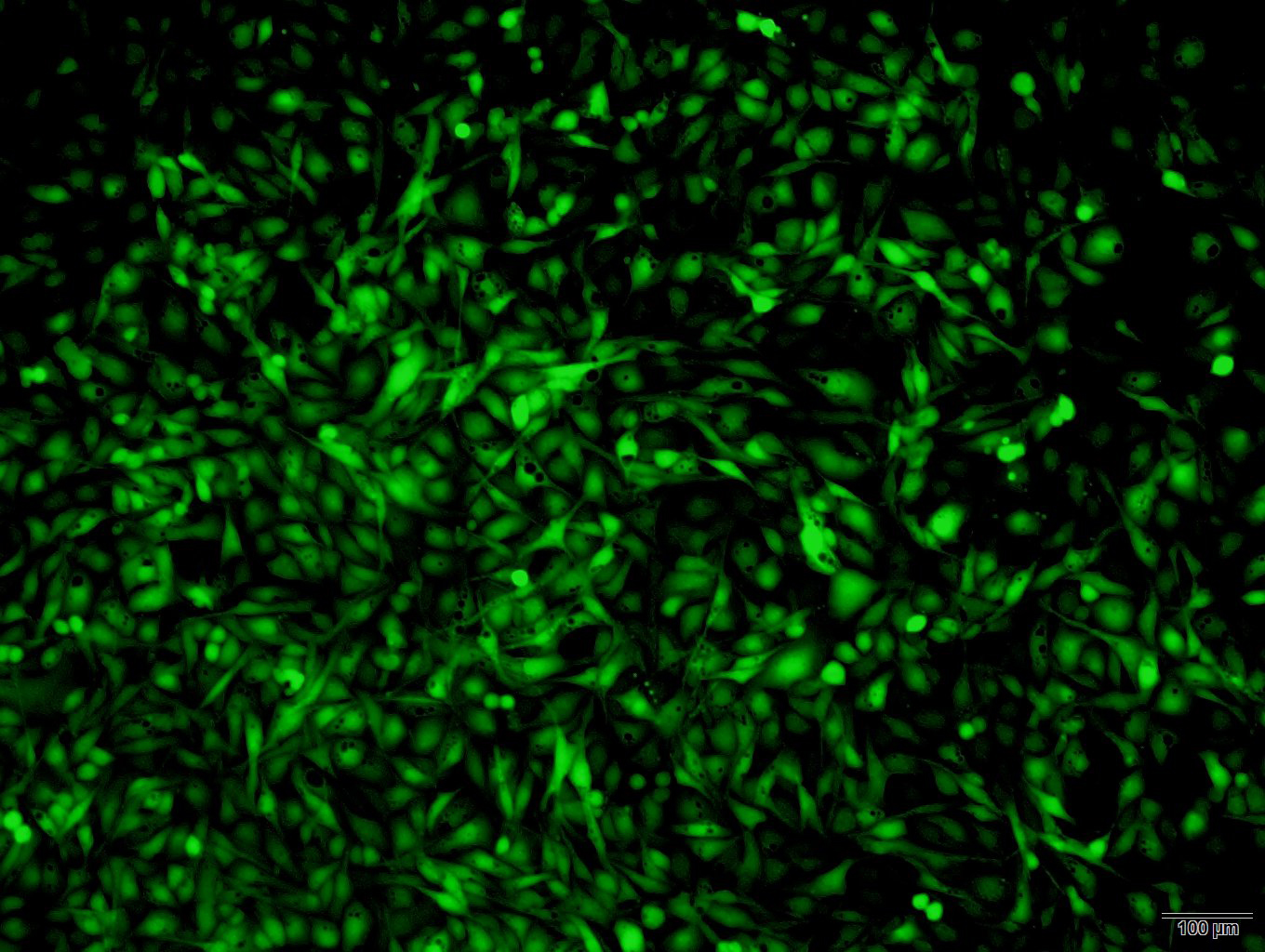

Supplement: Supplementary file 3 [file DataSheet2.ZIP › Cell experiment-RAW DATA/Fig 5/Fig5B-ROS-Original Figures/Rosup.jpg]

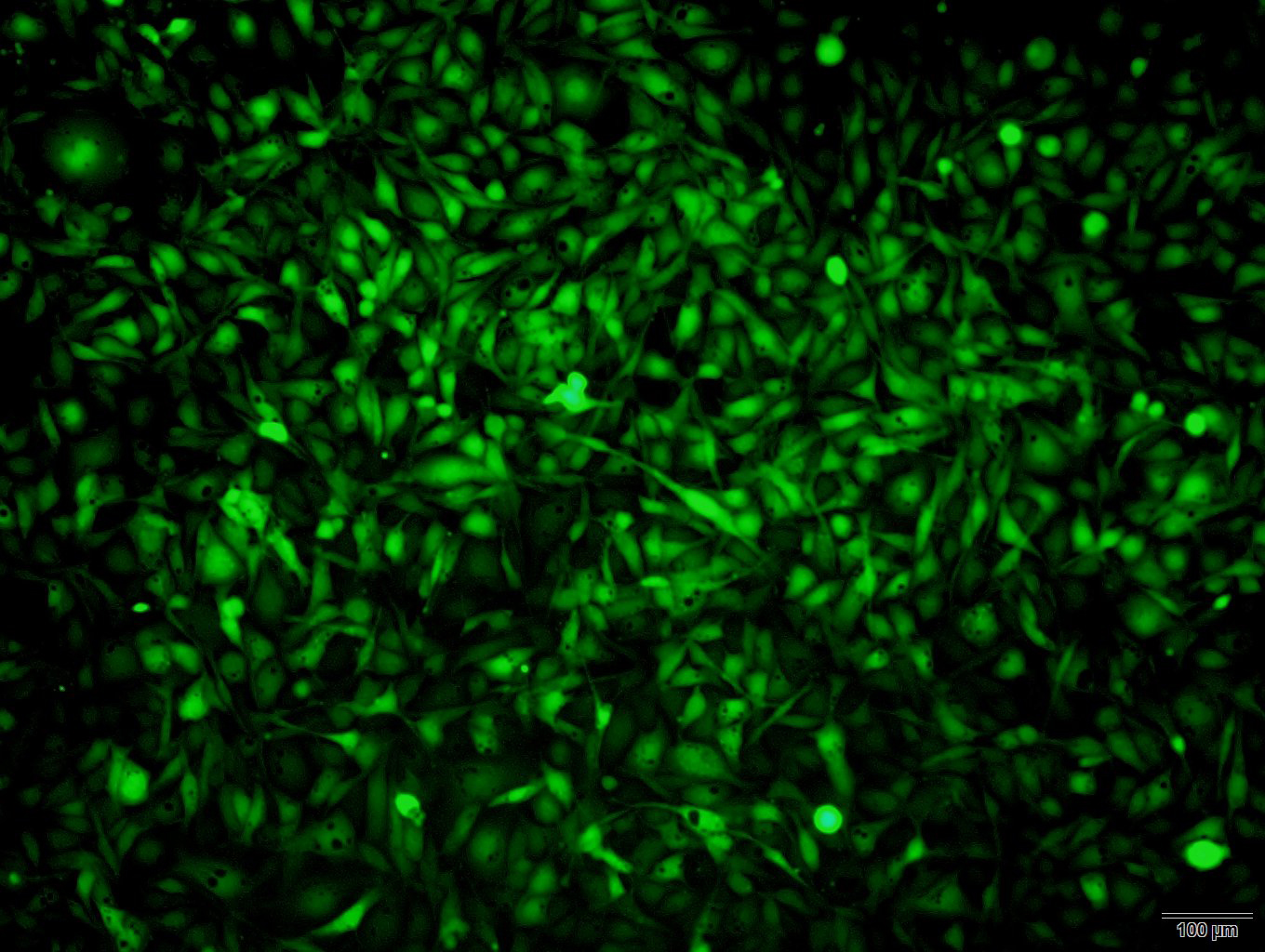

Supplement: Supplementary file 3 [file DataSheet2.ZIP › Cell experiment-RAW DATA/Fig 5/Fig5B-ROS-Original Figures/TM.jpg]

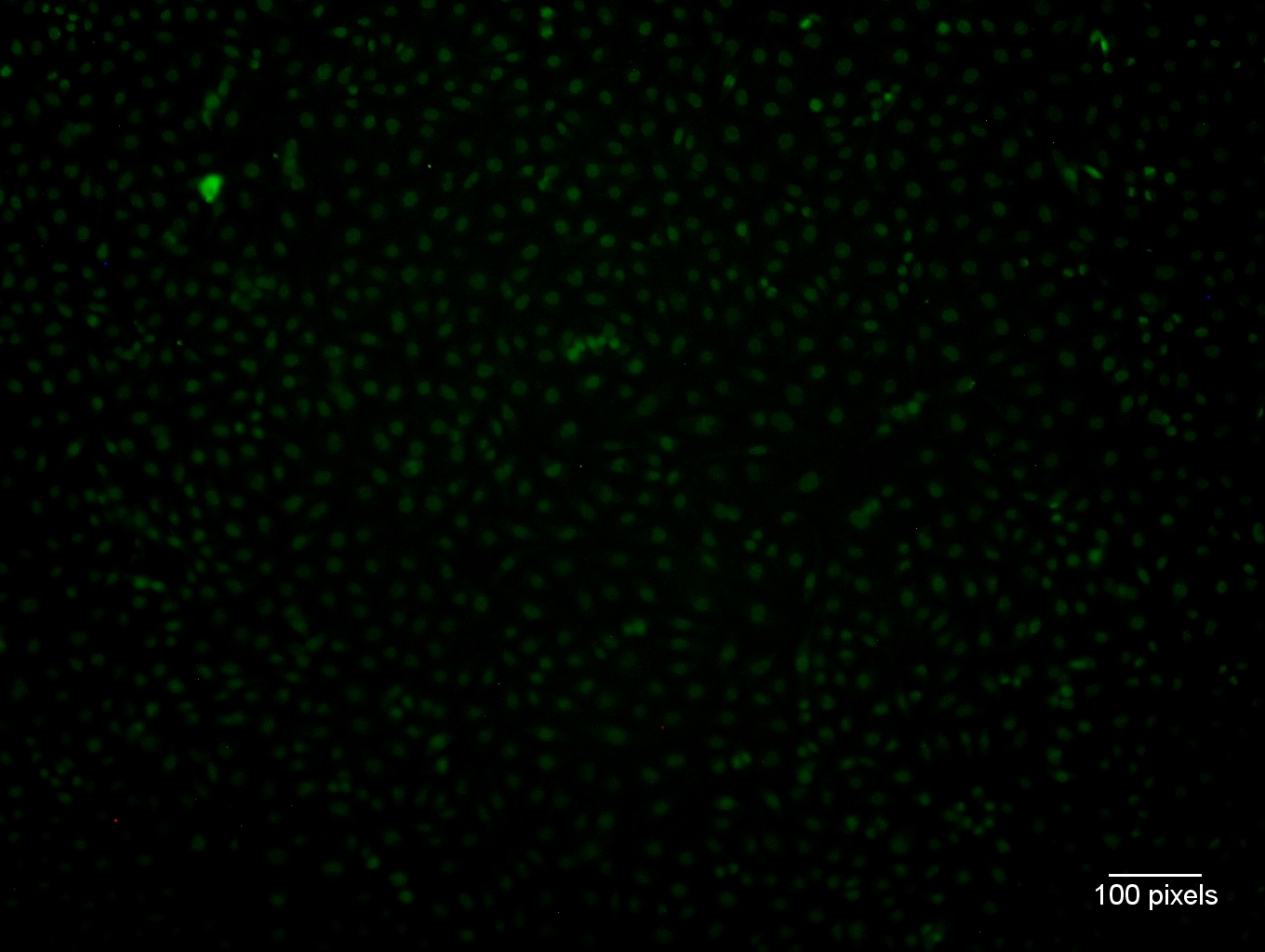

Supplement: Supplementary file 3 [file DataSheet2.ZIP › Cell experiment-RAW DATA/Fig 6/CON-CHOP.jpg]

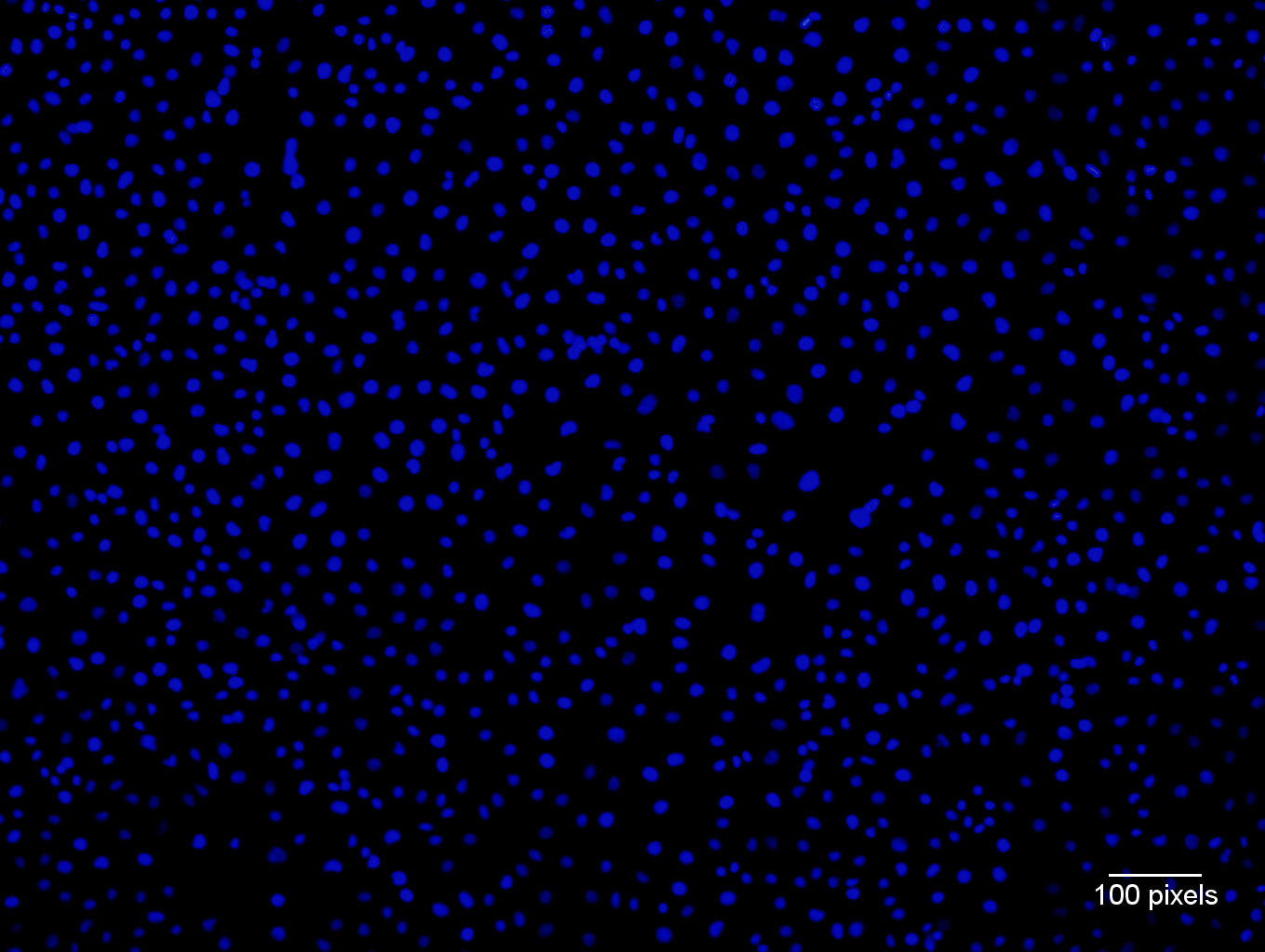

Supplement: Supplementary file 3 [file DataSheet2.ZIP › Cell experiment-RAW DATA/Fig 6/CON-DAPI.jpg]

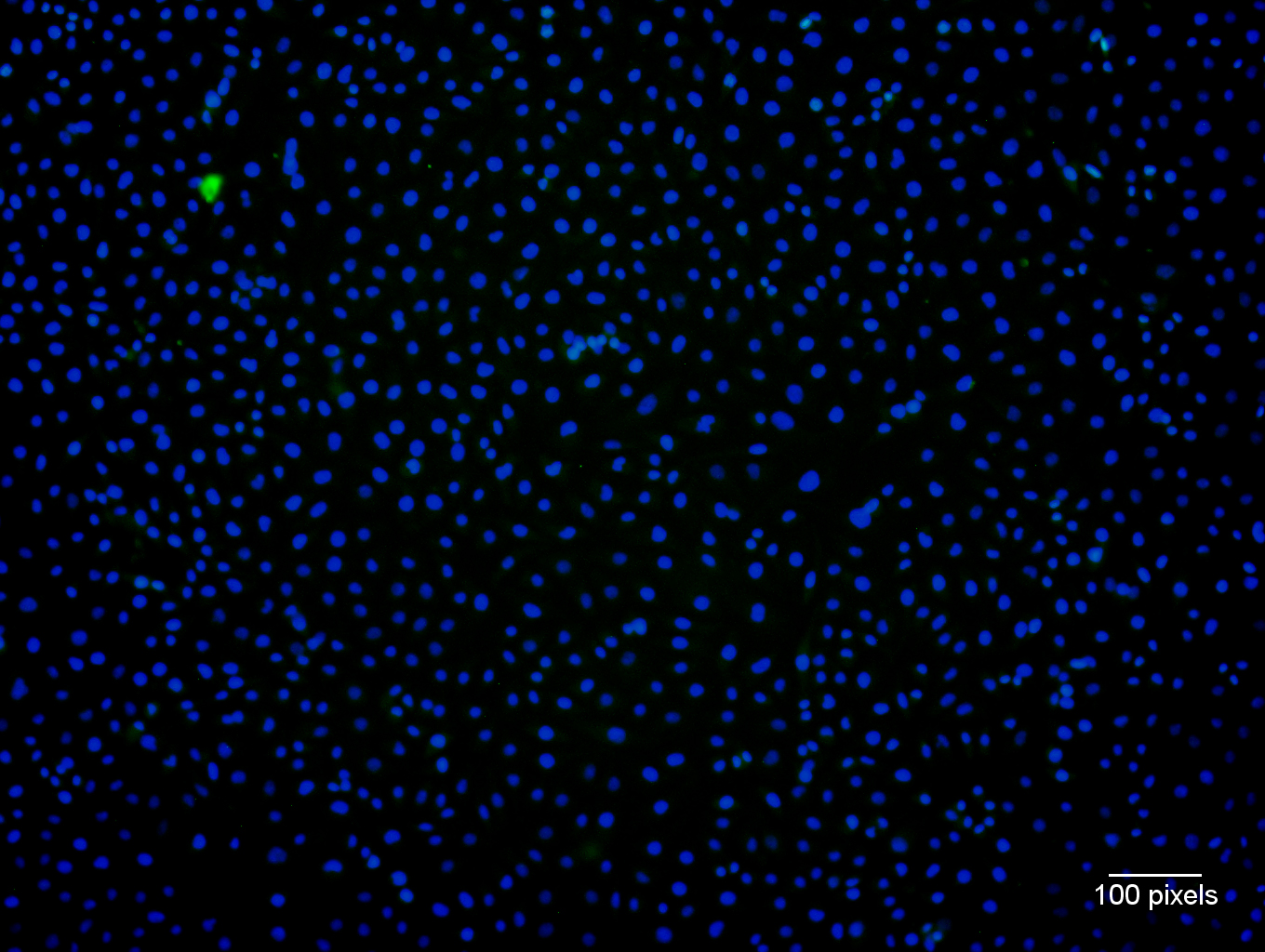

Supplement: Supplementary file 3 [file DataSheet2.ZIP › Cell experiment-RAW DATA/Fig 6/CON-Merge.jpg]

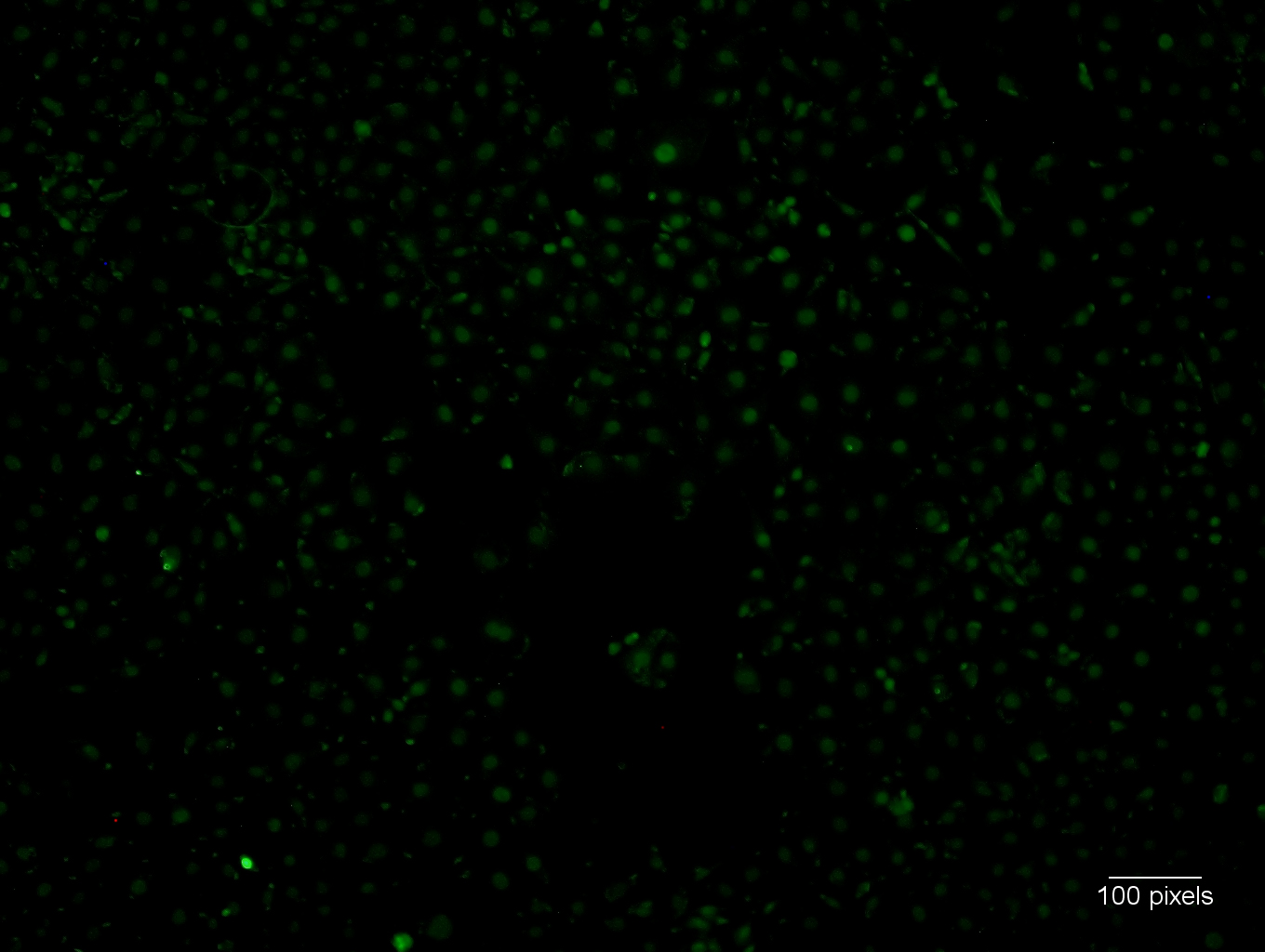

Supplement: Supplementary file 3 [file DataSheet2.ZIP › Cell experiment-RAW DATA/Fig 6/PA+SIT-H-CHOP.jpg]

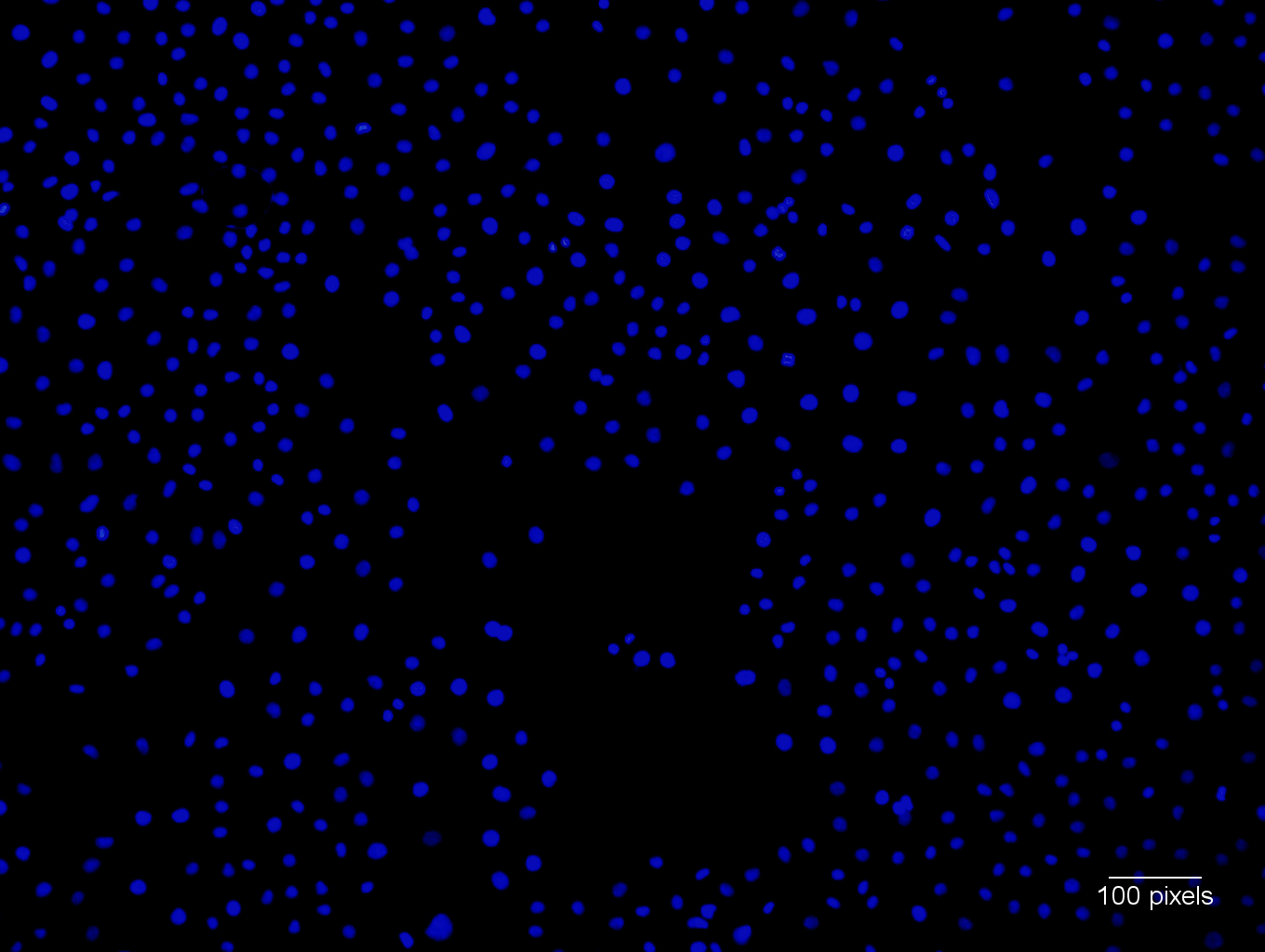

Supplement: Supplementary file 3 [file DataSheet2.ZIP › Cell experiment-RAW DATA/Fig 6/PA+SIT-H-DAPI.jpg]

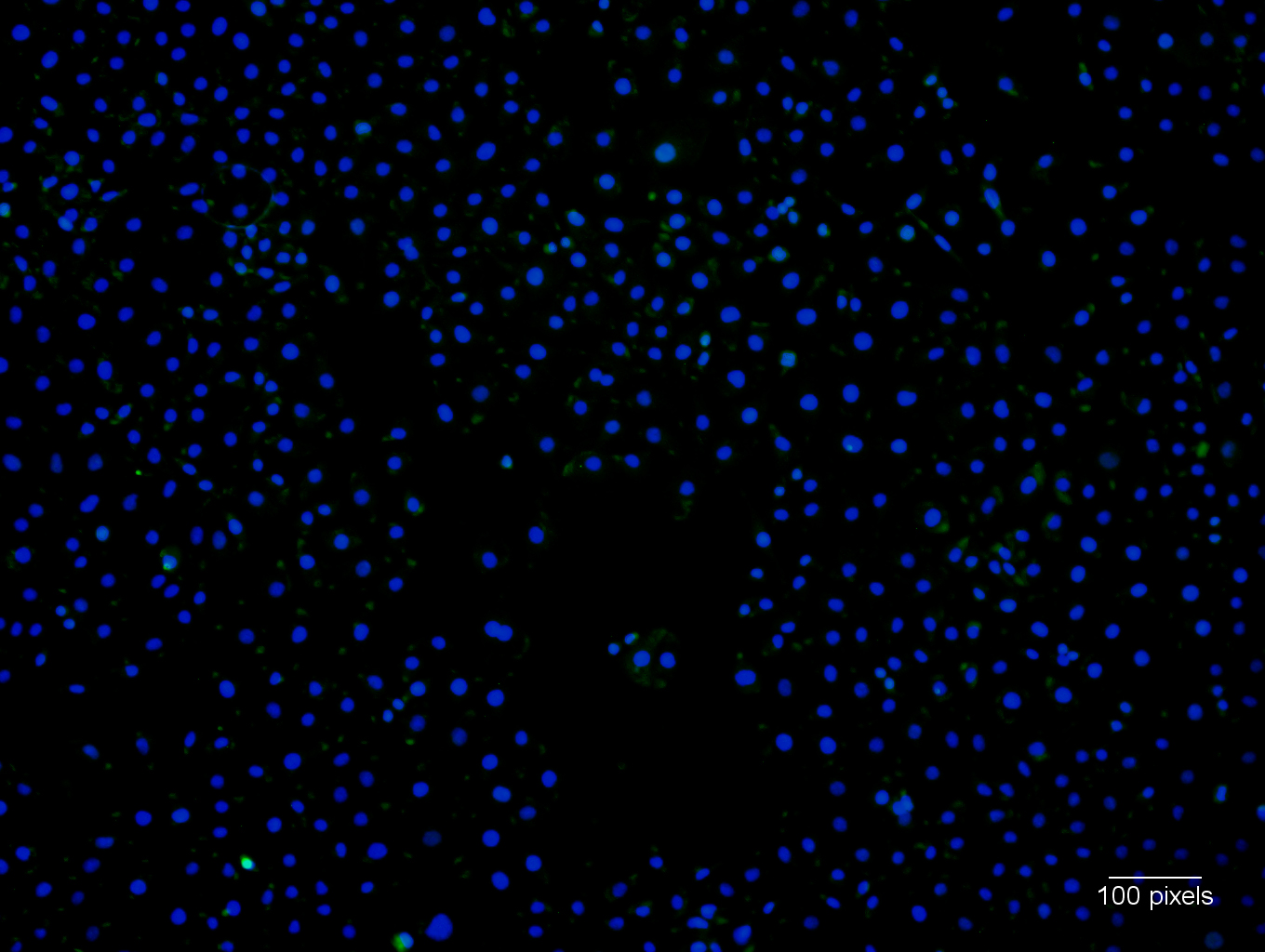

Supplement: Supplementary file 3 [file DataSheet2.ZIP › Cell experiment-RAW DATA/Fig 6/PA+SIT-H-Merge.jpg]

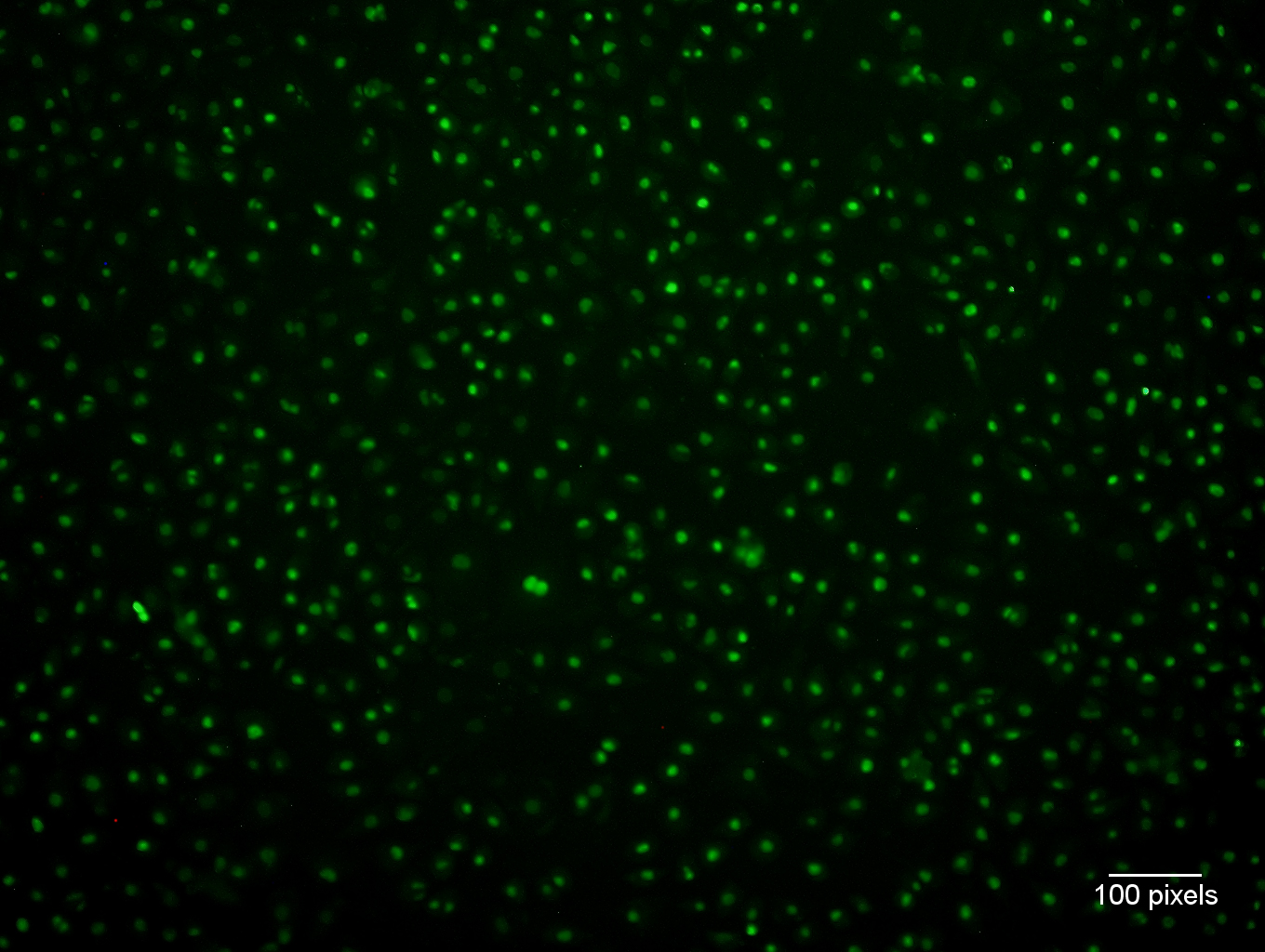

Supplement: Supplementary file 3 [file DataSheet2.ZIP › Cell experiment-RAW DATA/Fig 6/PA+SIT-L-CHOP.jpg]

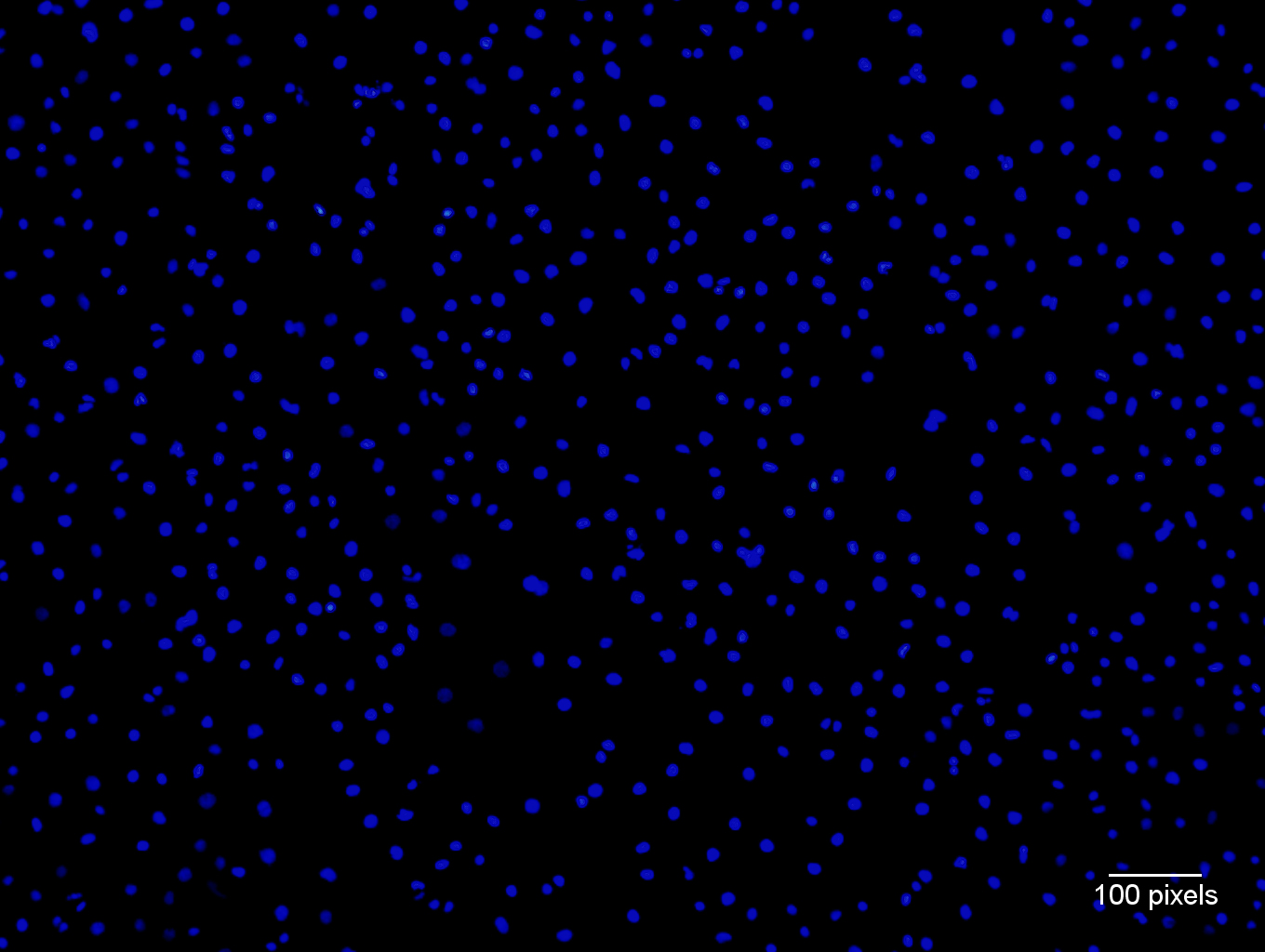

Supplement: Supplementary file 3 [file DataSheet2.ZIP › Cell experiment-RAW DATA/Fig 6/PA+SIT-L-DAPI.jpg]

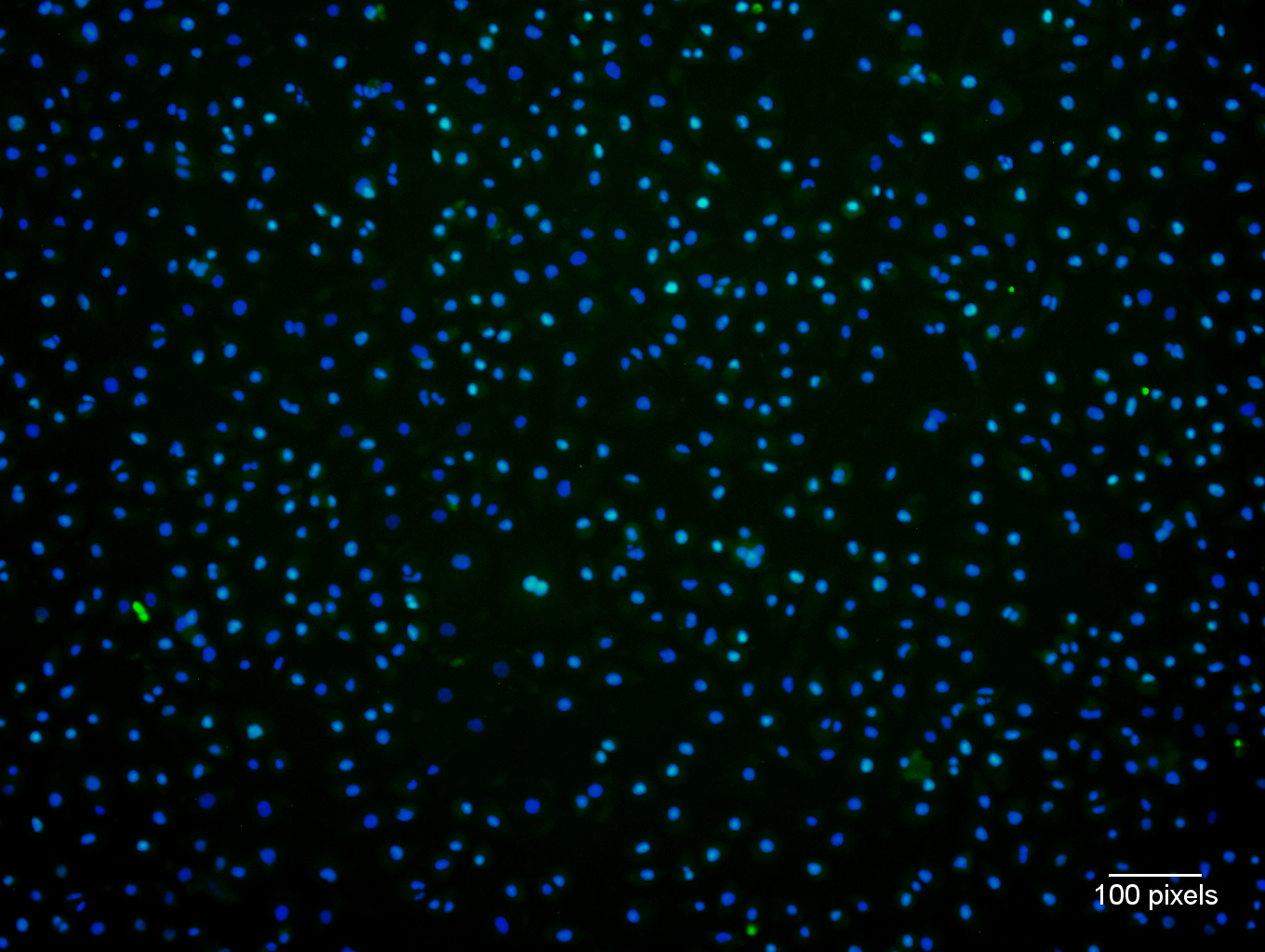

Supplement: Supplementary file 3 [file DataSheet2.ZIP › Cell experiment-RAW DATA/Fig 6/PA+SIT-L-Merge.jpg]

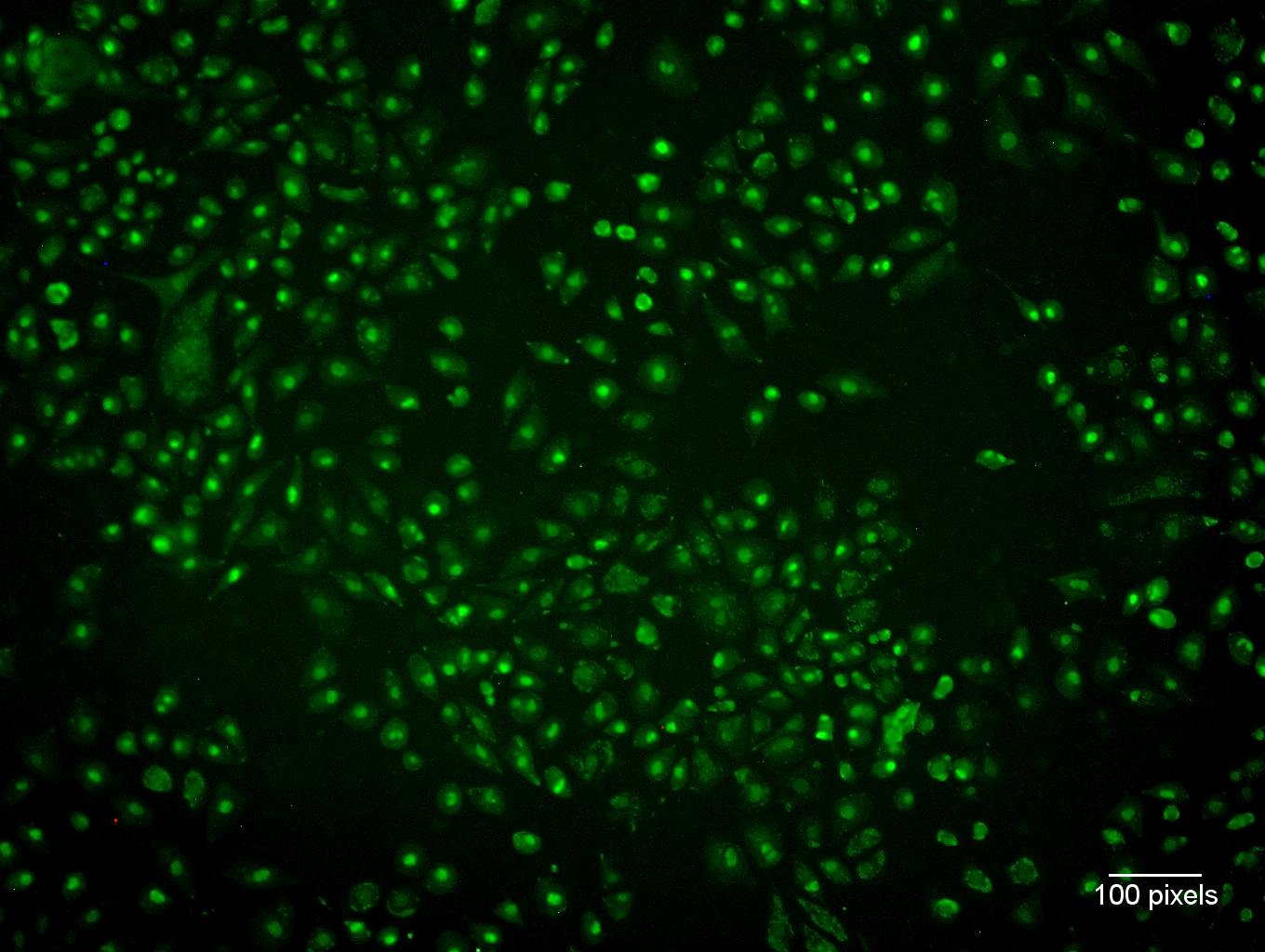

Supplement: Supplementary file 3 [file DataSheet2.ZIP › Cell experiment-RAW DATA/Fig 6/PA-CHOP.jpg]

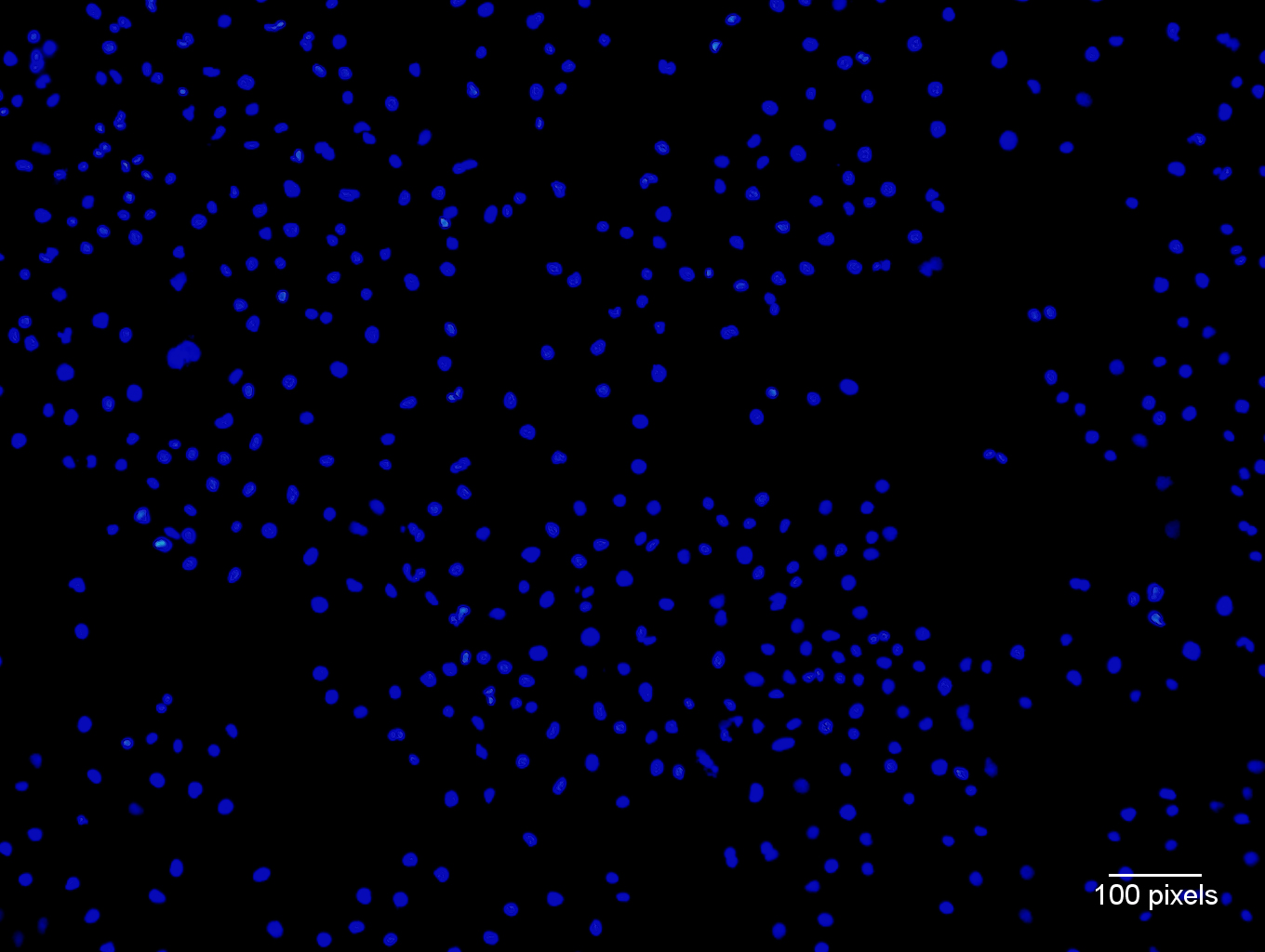

Supplement: Supplementary file 3 [file DataSheet2.ZIP › Cell experiment-RAW DATA/Fig 6/PA-DAPI.jpg]

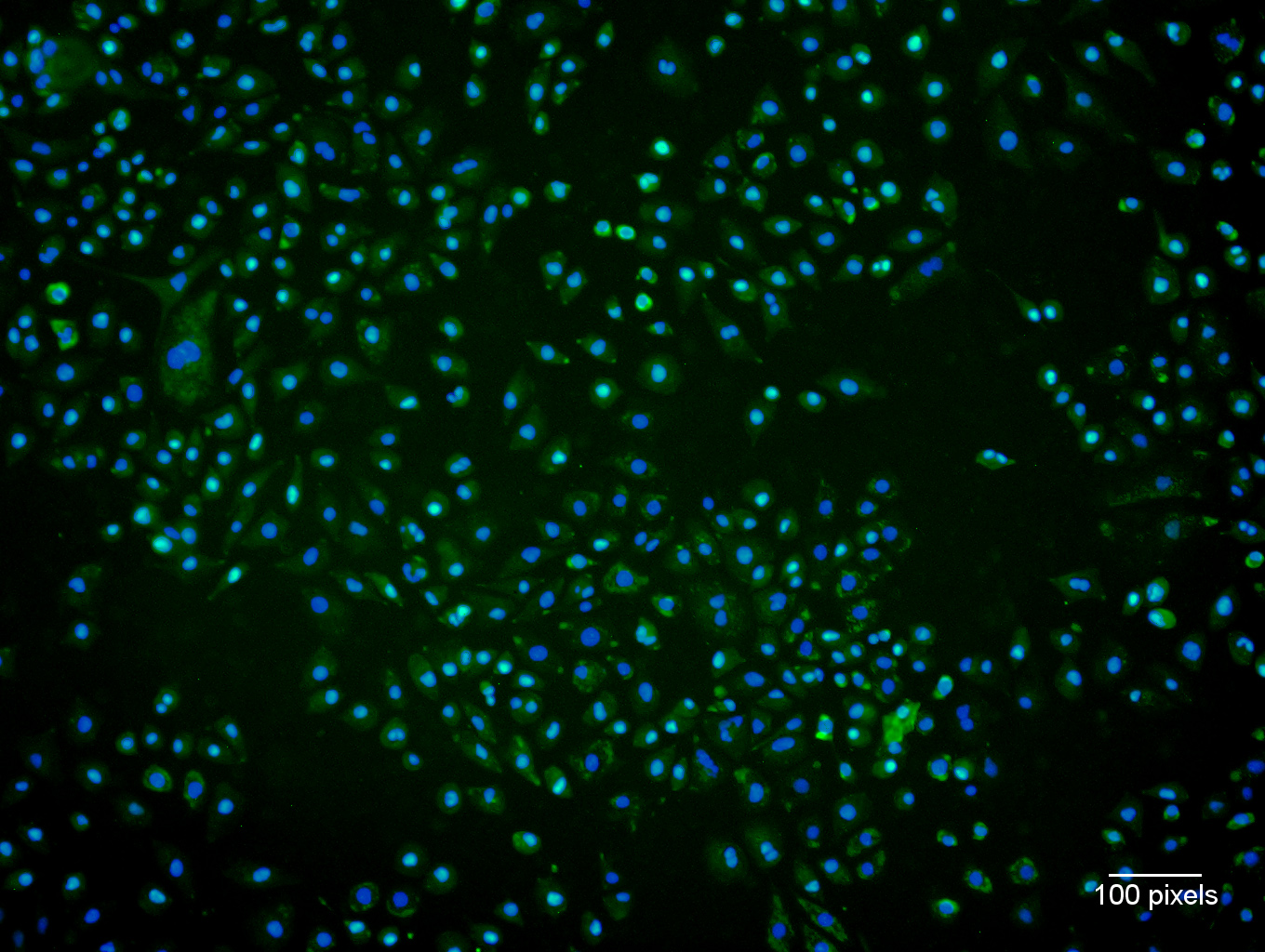

Supplement: Supplementary file 3 [file DataSheet2.ZIP › Cell experiment-RAW DATA/Fig 6/PA-Merge.jpg]

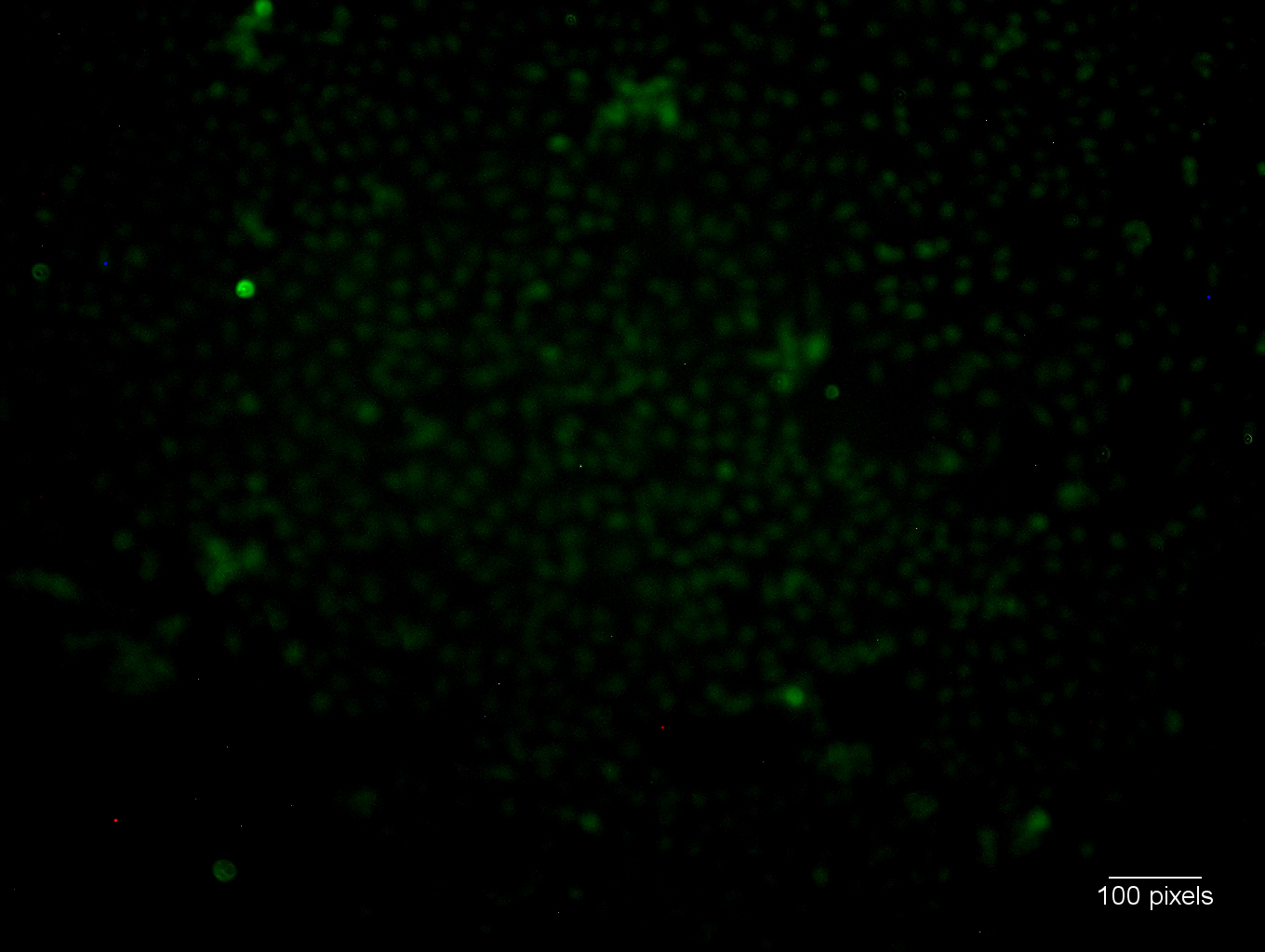

Supplement: Supplementary file 3 [file DataSheet2.ZIP › Cell experiment-RAW DATA/Fig 6/PBA-CHOP.jpg]

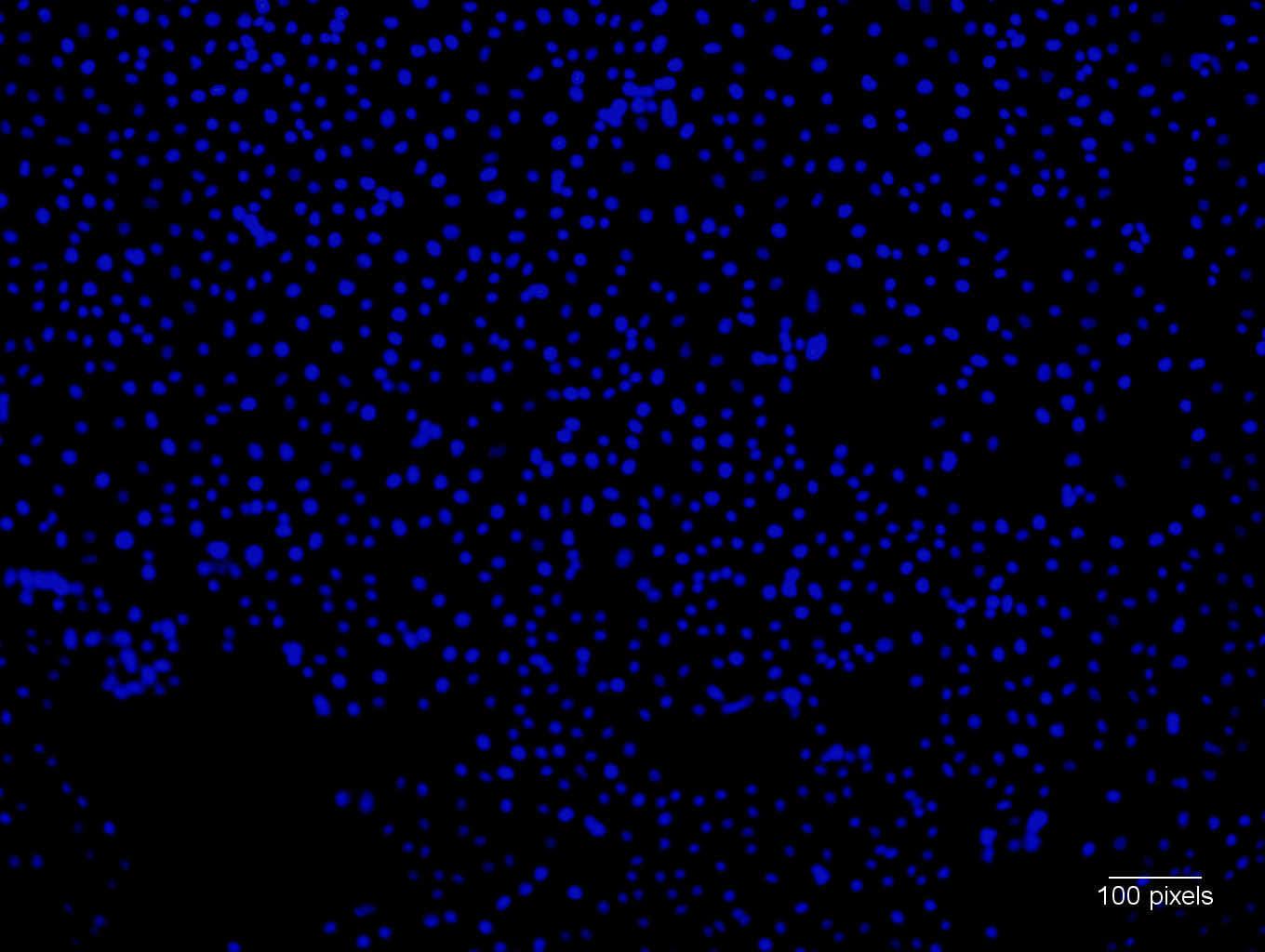

Supplement: Supplementary file 3 [file DataSheet2.ZIP › Cell experiment-RAW DATA/Fig 6/PBA-DAPI.jpg]

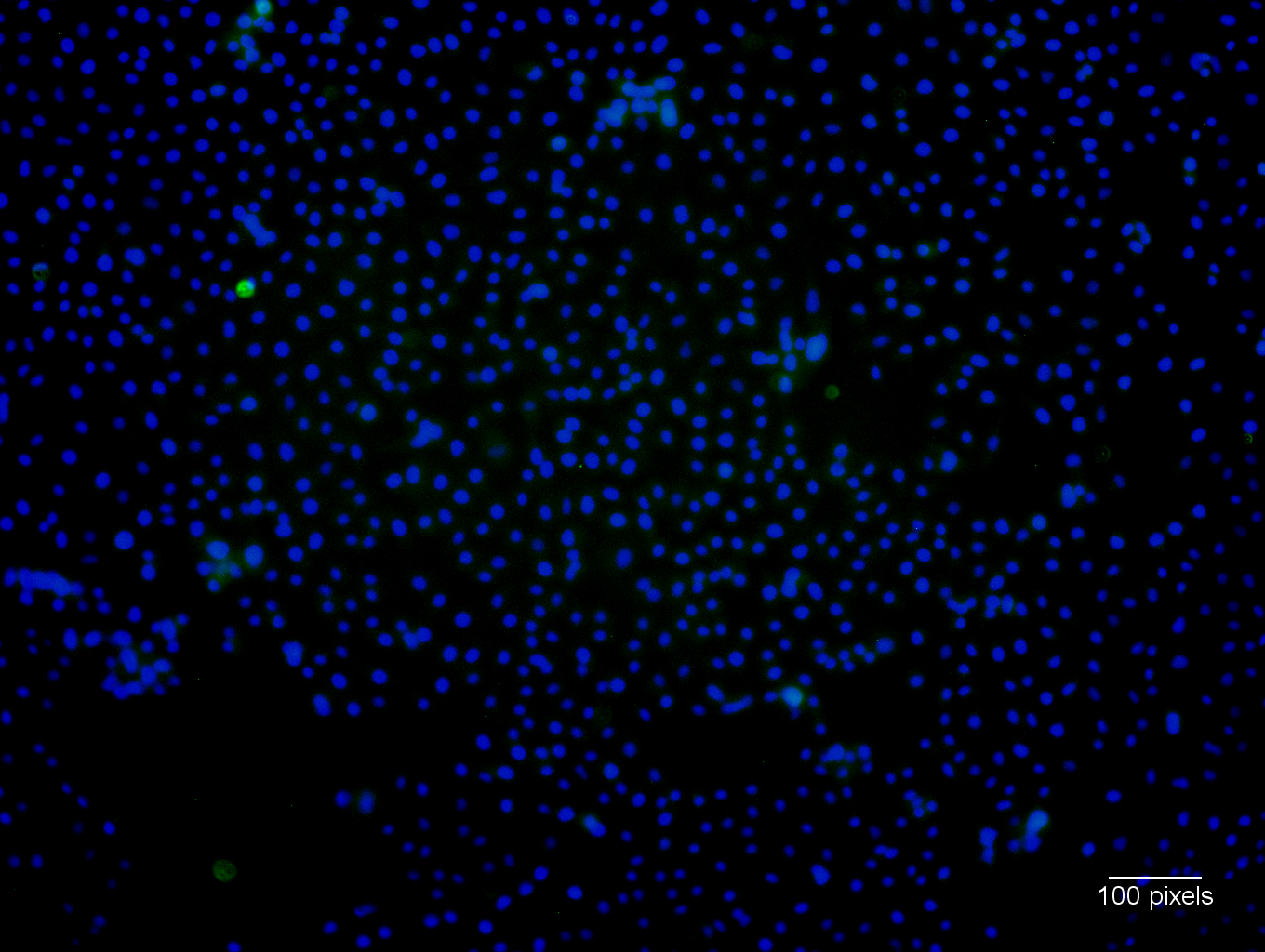

Supplement: Supplementary file 3 [file DataSheet2.ZIP › Cell experiment-RAW DATA/Fig 6/PBA-Merge.jpg]

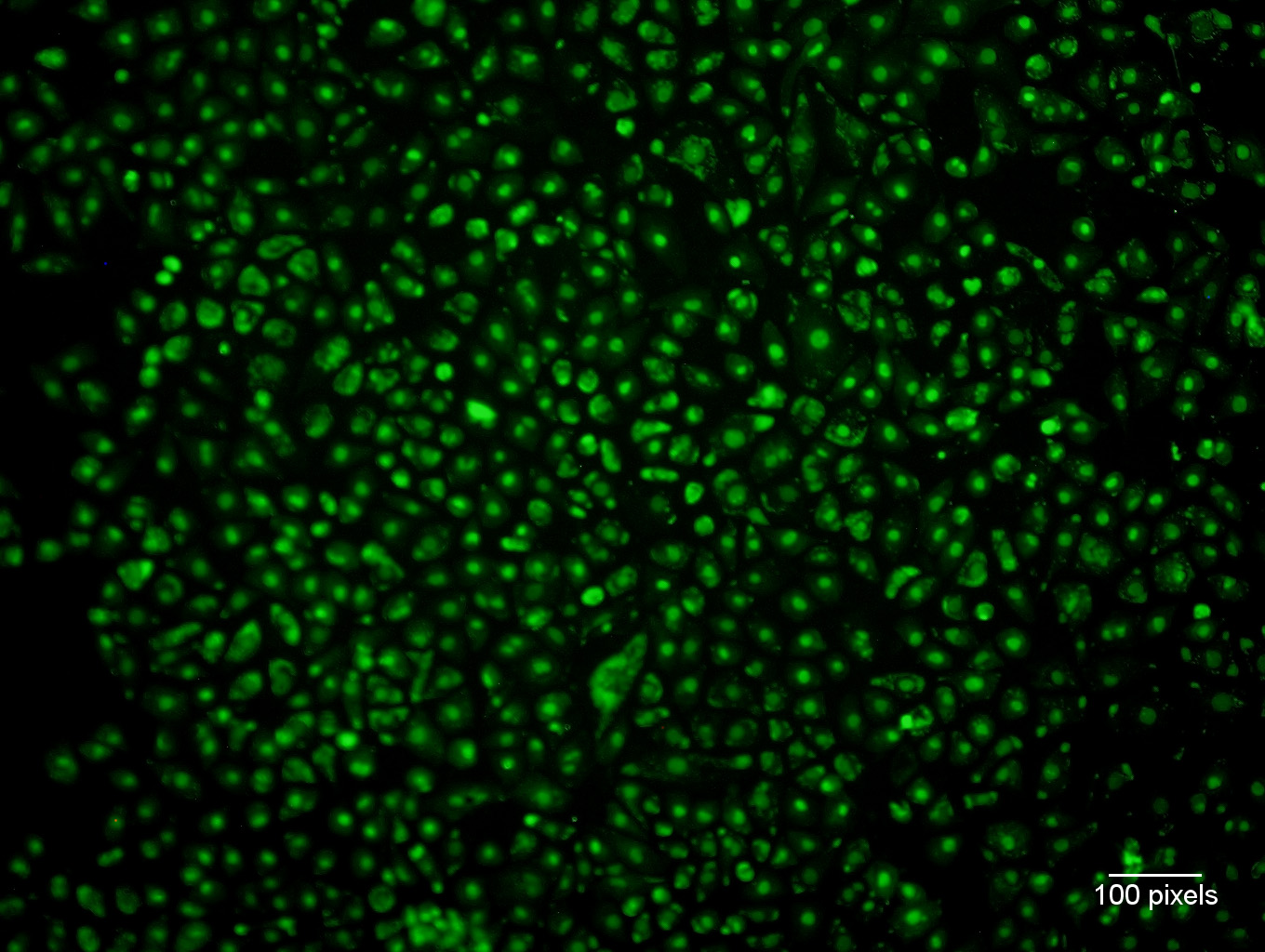

Supplement: Supplementary file 3 [file DataSheet2.ZIP › Cell experiment-RAW DATA/Fig 6/TM-CHOP.jpg]

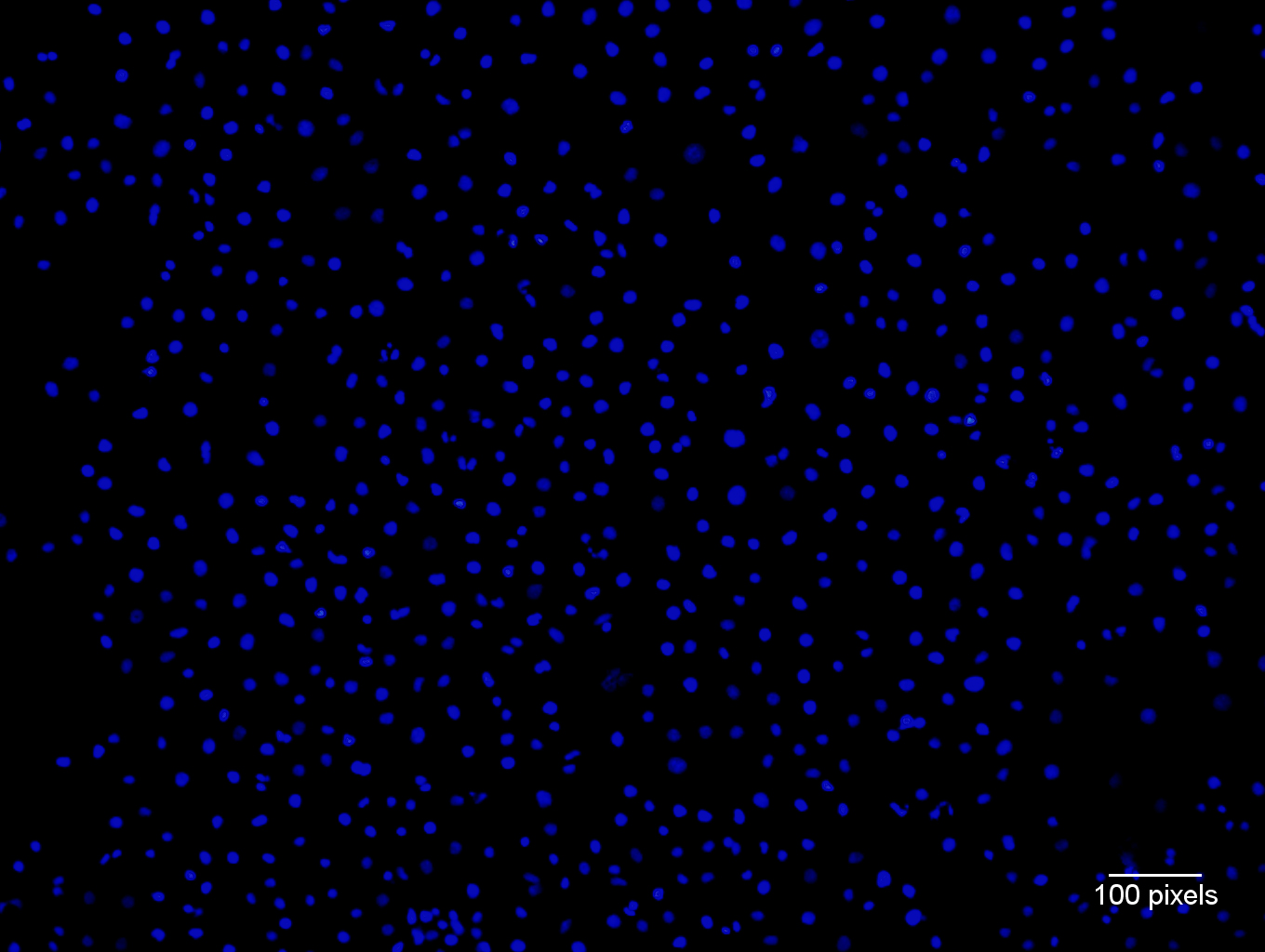

Supplement: Supplementary file 3 [file DataSheet2.ZIP › Cell experiment-RAW DATA/Fig 6/TM-DAPI.jpg]

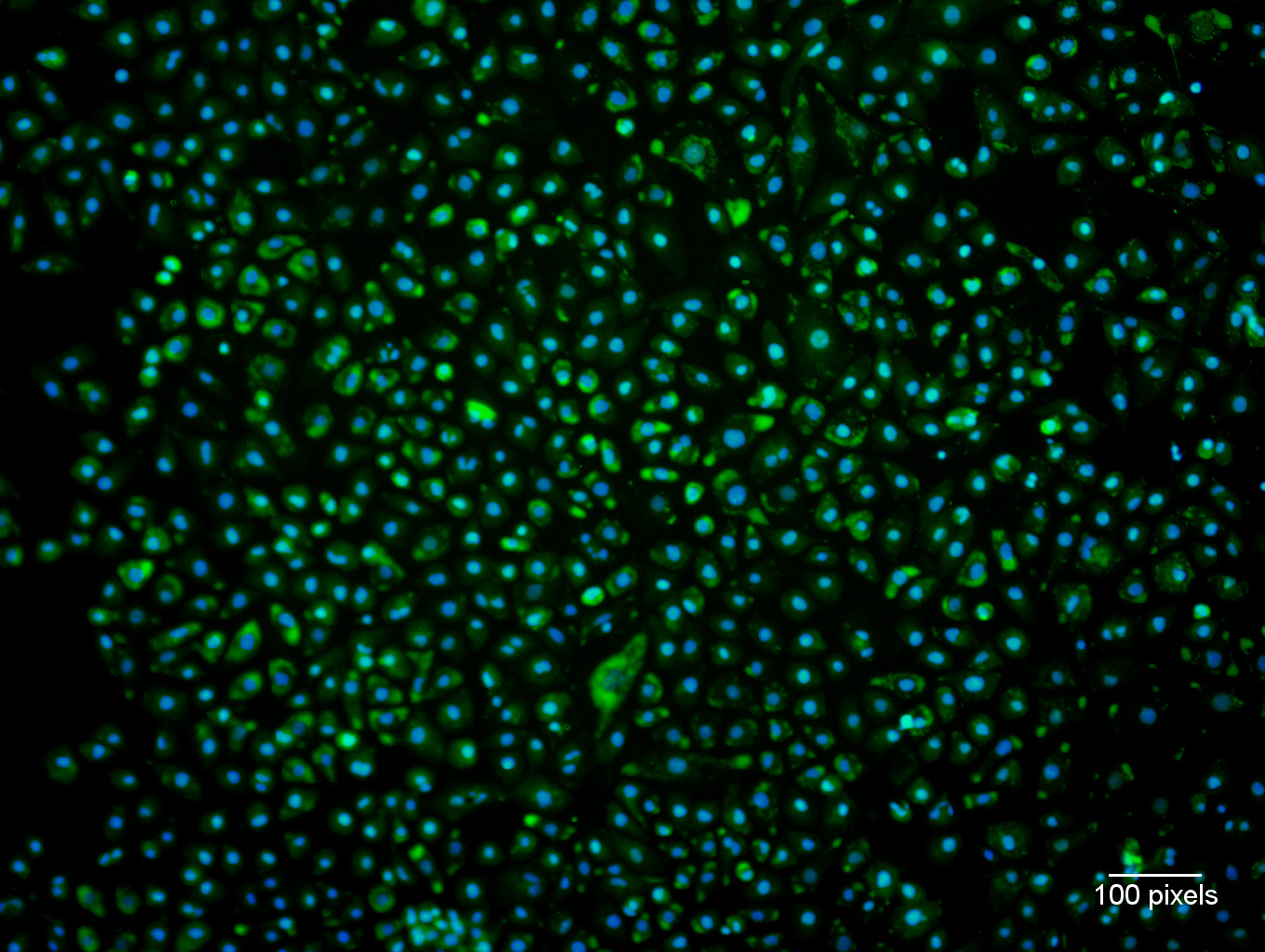

Supplement: Supplementary file 3 [file DataSheet2.ZIP › Cell experiment-RAW DATA/Fig 6/TM-Merge.jpg]

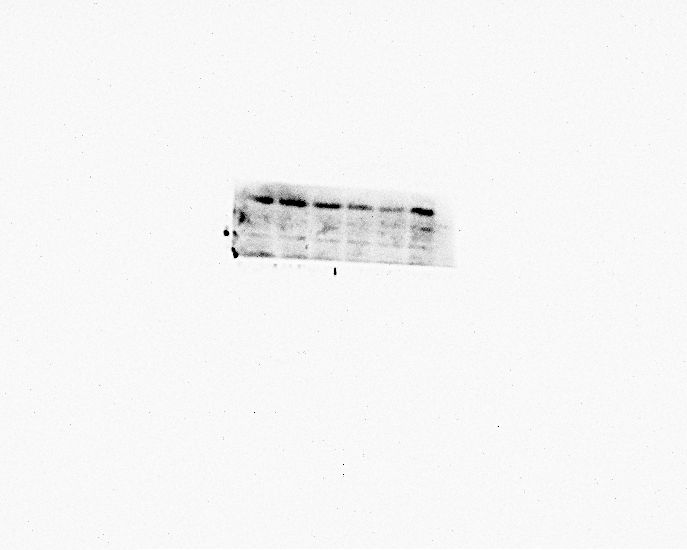

Supplement: Supplementary file 3 [file DataSheet2.ZIP › Cell experiment-RAW DATA/Fig 7/Original WB figures/BAX.jpg]

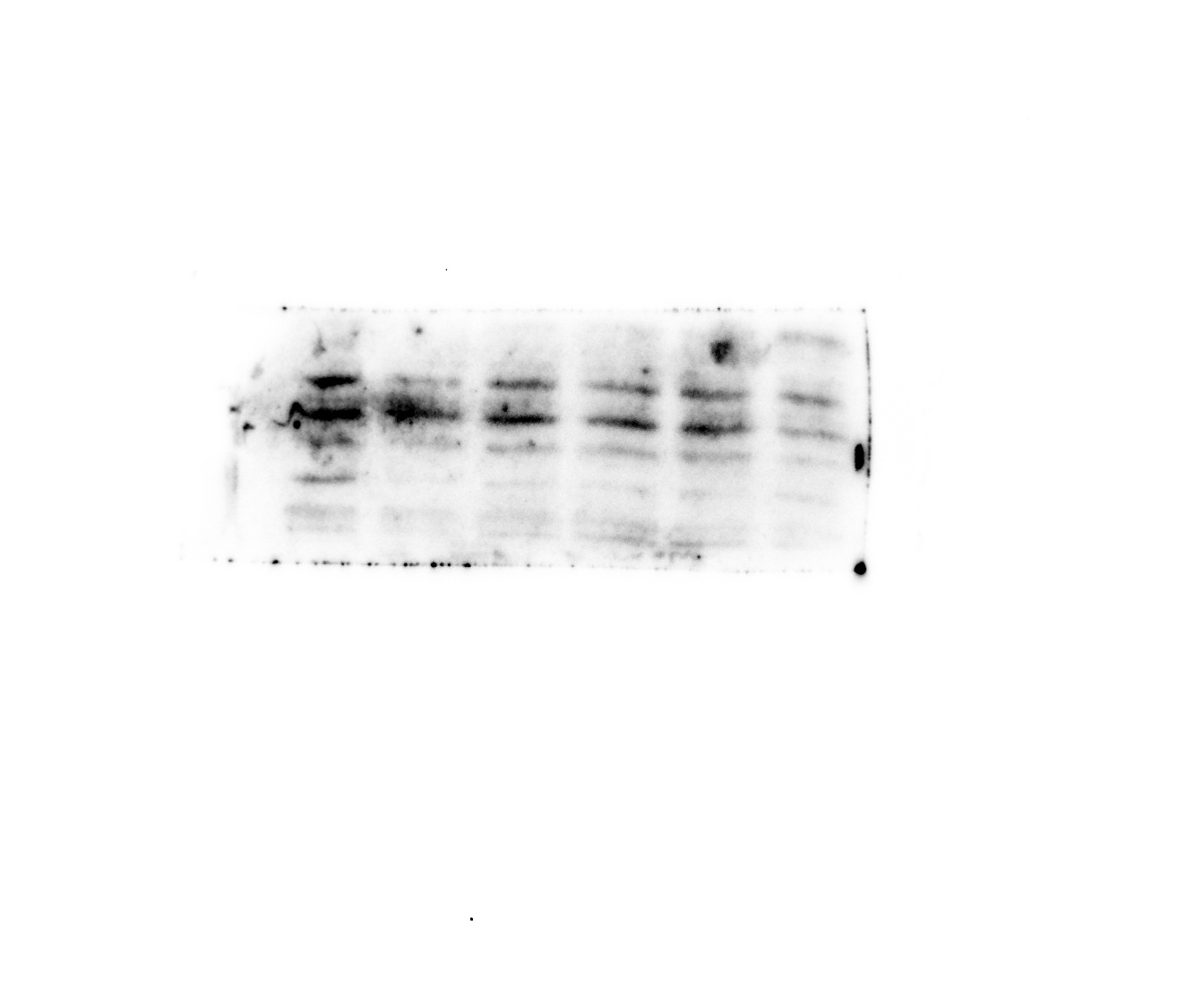

Supplement: Supplementary file 3 [file DataSheet2.ZIP › Cell experiment-RAW DATA/Fig 7/Original WB figures/Bcl-2.jpg]

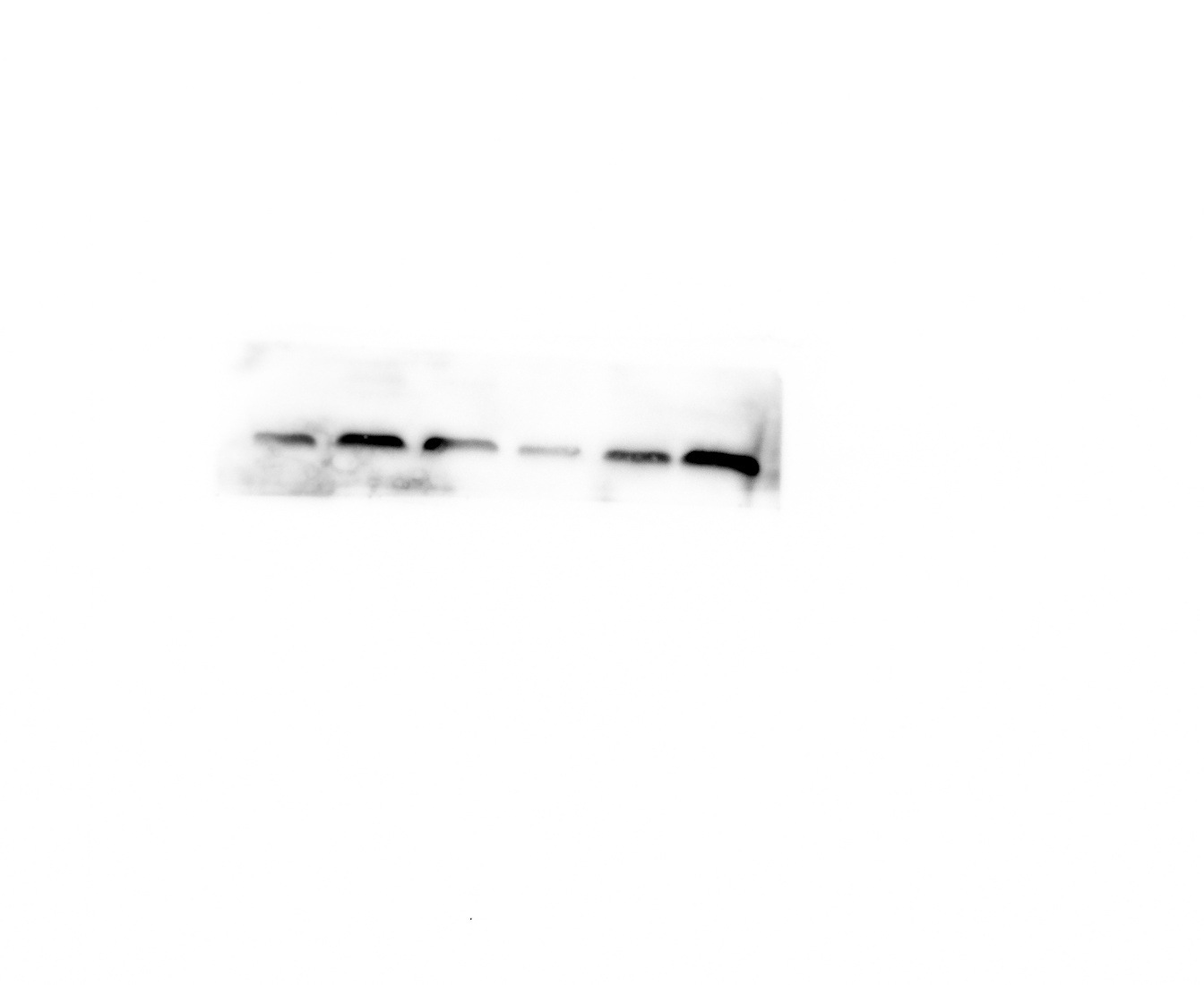

Supplement: Supplementary file 3 [file DataSheet2.ZIP › Cell experiment-RAW DATA/Fig 7/Original WB figures/CHOP.jpg]

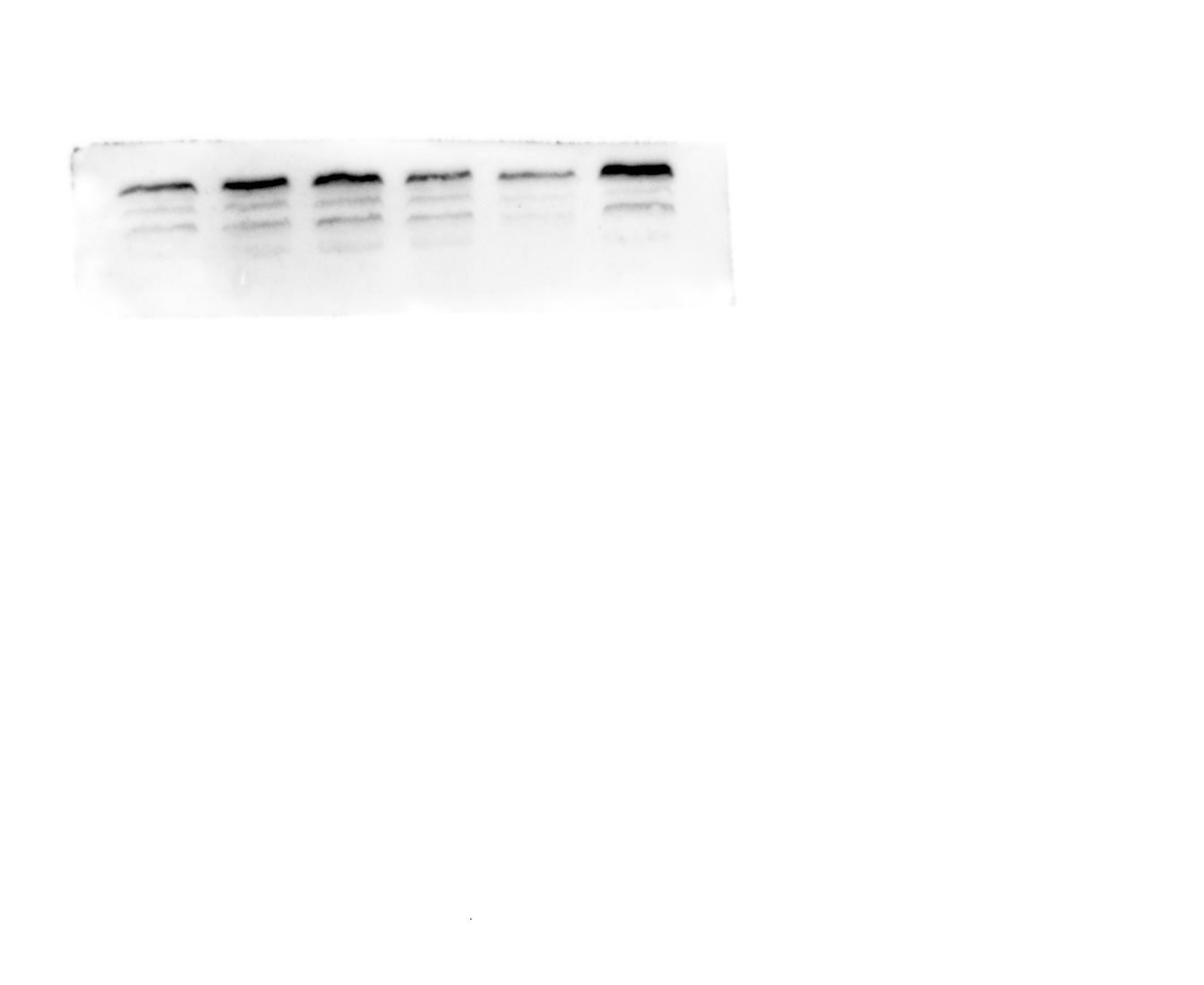

Supplement: Supplementary file 3 [file DataSheet2.ZIP › Cell experiment-RAW DATA/Fig 7/Original WB figures/GRP78.jpg]

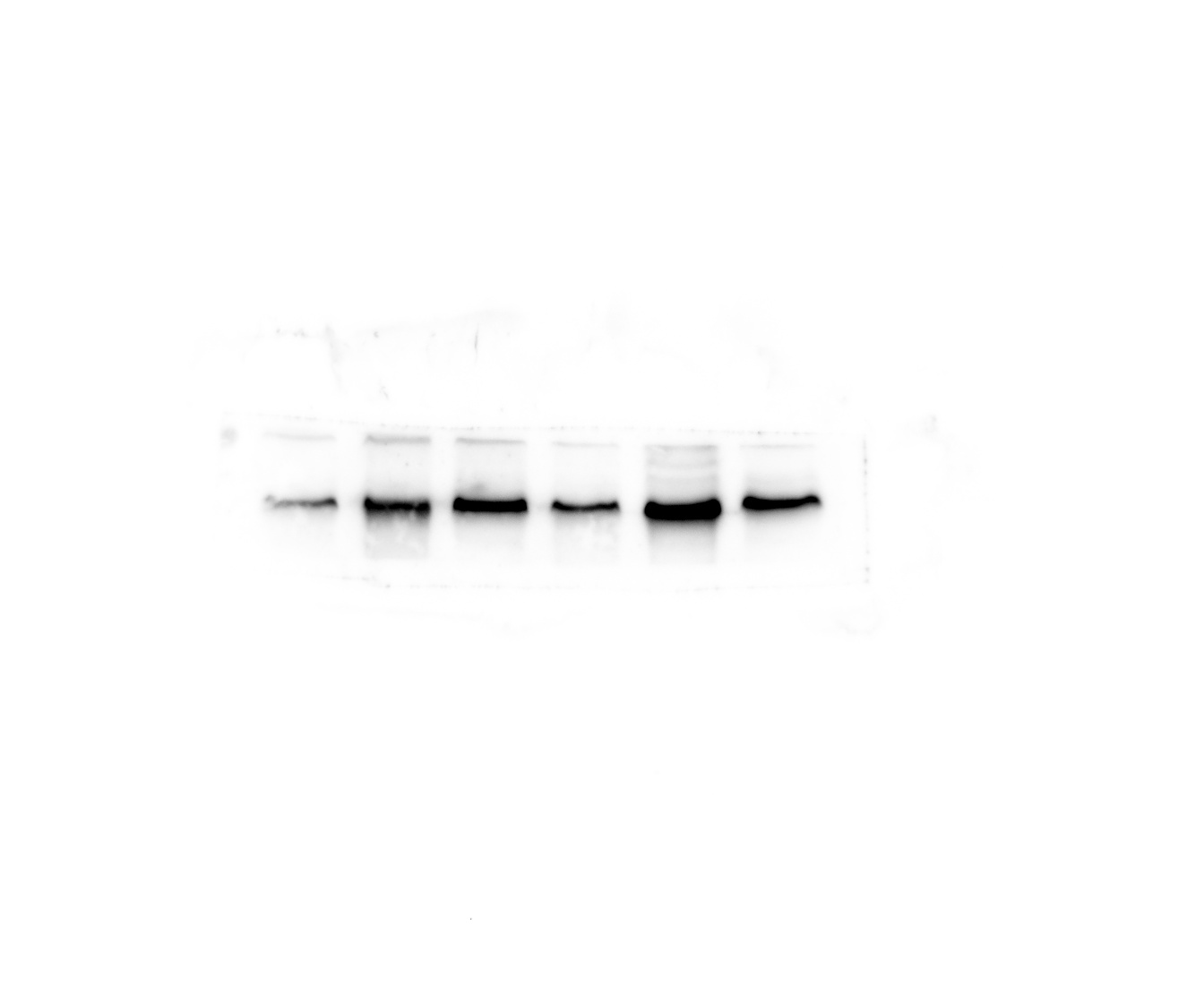

Supplement: Supplementary file 3 [file DataSheet2.ZIP › Cell experiment-RAW DATA/Fig 7/Original WB figures/IRE-1a┴.jpg]

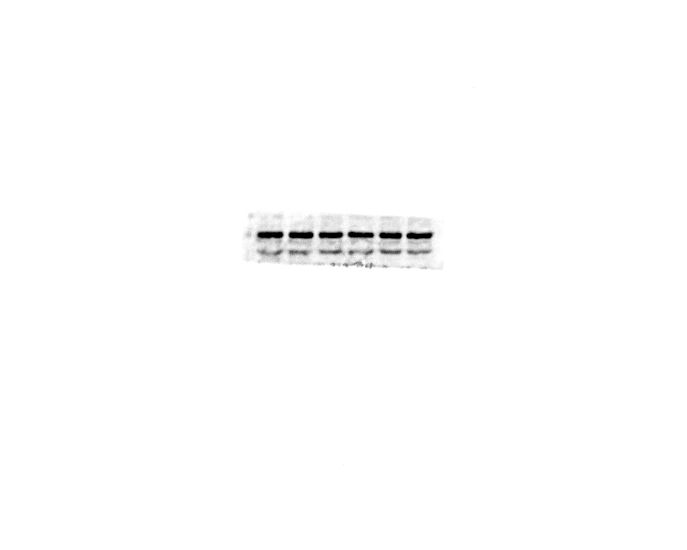

Supplement: Supplementary file 3 [file DataSheet2.ZIP › Cell experiment-RAW DATA/Fig 7/Original WB figures/JNK.jpg]

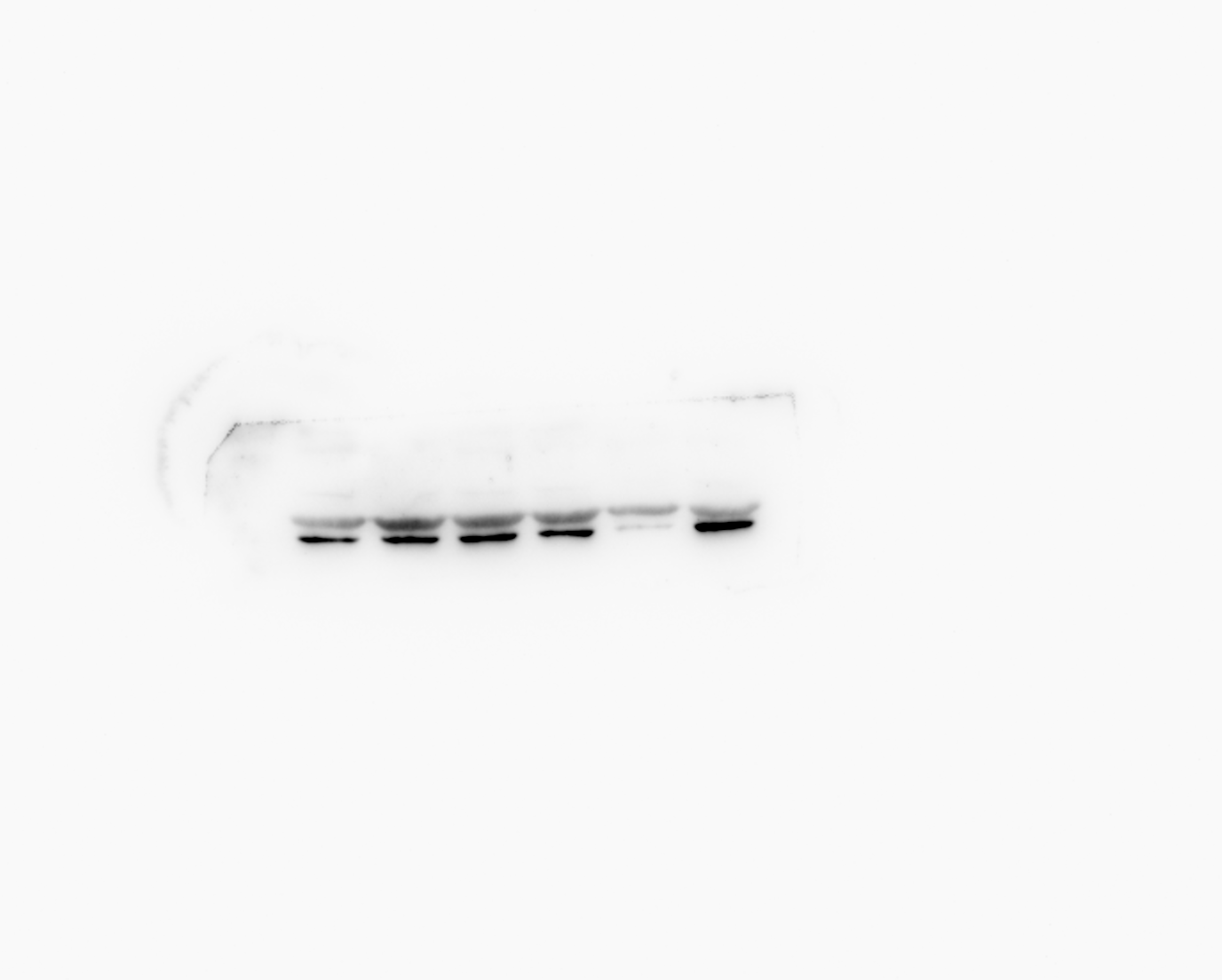

Supplement: Supplementary file 3 [file DataSheet2.ZIP › Cell experiment-RAW DATA/Fig 7/Original WB figures/P-JNK.tif]

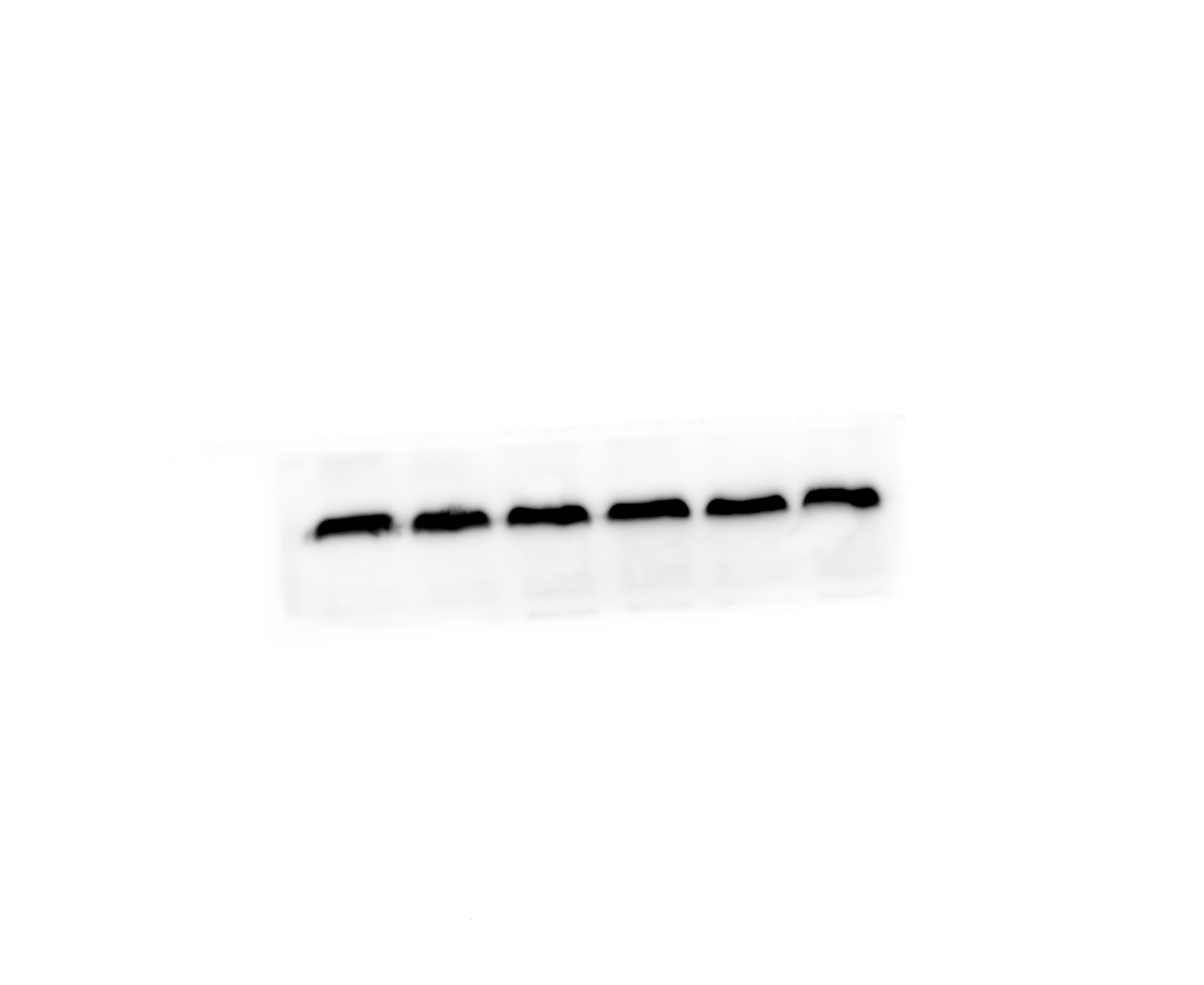

Supplement: Supplementary file 3 [file DataSheet2.ZIP › Cell experiment-RAW DATA/Fig 7/Original WB figures/actin.jpg]

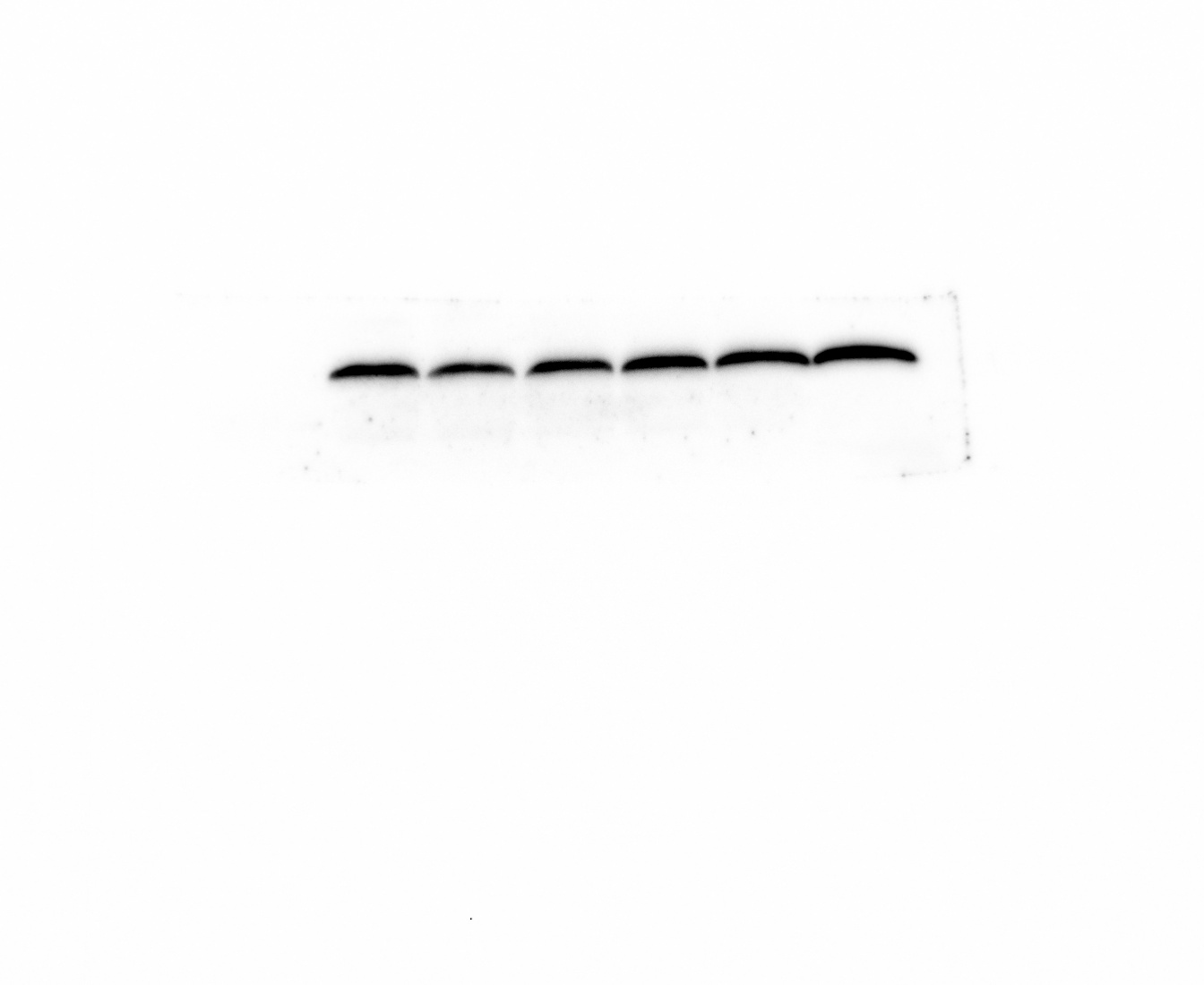

Supplement: Supplementary file 3 [file DataSheet2.ZIP › Cell experiment-RAW DATA/Fig 7/Original WB figures/eNOS.jpg]

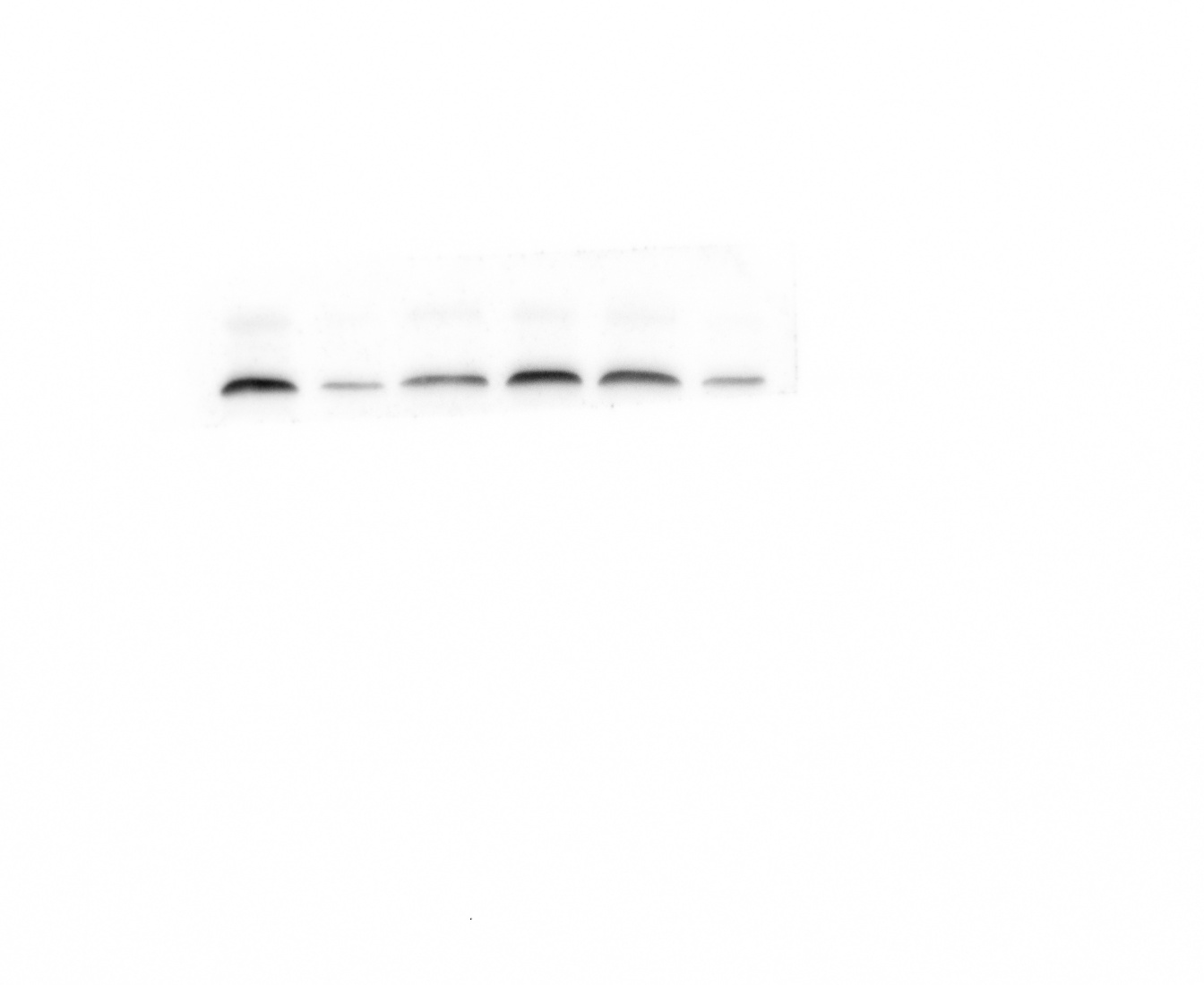

Supplement: Supplementary file 3 [file DataSheet2.ZIP › Cell experiment-RAW DATA/Fig 7/Original WB figures/p-eNOS.jpg]
